# Supplementary material for: FLUid management and InDividualized resuscitation in Sepsis (FLUIDS)—A Study protocol for a single-centre, open-label, randomized clinical trial
Source: PLoS One. 2025 Dec 19;20(12):e0338504. doi: 10.1371/journal.pone.0338504 (PMC12716701; doi:10.1371/journal.pone.0338504)
Supplement: S1 File — Supplementary material detailing the technical protocol for performing point-of-care ultrasound assessments of the inferior vena cava (IVC), venous congestion (VExUS), and bedside lung ultrasound in emergency (BLUE) in the FLUIDS trial. (DOCX) [file pone.0338504.s001.docx]

**Supplementary A: Point-of-Care Ultrasound Technical Performance in the FLUIDS Study**

***Inferior Vena Cava (IVC), Venous Excess Ultrasound Score (VExUS), Renal Resistive Index (RRI), Venous Impedance Index (VII), and Bedside Lung Ultrasound in Emergency (BLUE) Protocol***

**Pre-examination guideline:**

1. Start GE Venue Apparatus or Sonosite Apparatus, log in if required
2. Note FLUIDS ID number
3. Ensure the following items are present: echo gel, towels, cleaning wipes, disinfection wipes for ultrasound (US) machine
4. Stand or sit on the right side of the patient
5. Position patient in supine position (if possible)
6. Dim the light
7. Collect data in the following order: inferior vena cava (IVC), VExUS (hepatic vein (HV), portal vein (PV), and interlobar renal vein (IRV)) with RRI and VII calculated from renal flow patterns, and BLUE points

**Inferior Vena Cava (IVC) Image Acquisition and Measurement**

| Frequency | 1-5 MHz |
| --- | --- |
| Depth | 15-20 cm (80/20 rule) |
| Probe | Curvilinear probe |
| Location | Right of and caudal to xiphoidal process, lightly aimed cranial (12 o’clock) |
| Patient Position | Supine |

**Image Acquisition**

1. Position the transducer in a subcostal view, just to the right of the xiphoid process, with the probe marker oriented cranially.
2. First identify the abdominal aorta, recognized by its pulsatile nature and hyperechoic walls. Then, tilt the probe to the patient’s right until the inferior vena cava (IVC) comes into view.
3. Confirm correct identification of the IVC using the following anatomical landmarks: entry into the right atrium, confluence with the hepatic vein, and its intrahepatic location.
4. Optimize the longitudinal view of the IVC at its maximum diameter, ensuring sharply defined, hyperechoic vessel walls.
5. Write “IVC” in the apparatus to indicate the visualized structure.

**Measurement Protocol**

1. Measure the IVC diameter during both end-inspiration and end-expiration at a standardized distance of 2–3 cm (preferably 2 cm) caudal to its junction with the right atrium (**see Figure A1**).
2. Alternatively, utilize the device’s automated IVC measurement feature, which calculates maximum and minimum diameters as well as collapsibility. Ensure that measurements are taken during a complete and deep respiratory cycle and at the right distance of right atrium.

**Image Documentation**

1. Save one video showing at least one respiratory cycle and two images displaying both the maximum (**see Figure A2**) and minimum diameter (**see Figure A3**) of the IVC.
2. If not using automated measurements, calculate IVC collapsibility manually using the following formula:
     *Collapsibility Index (%) = [(IVC_max − IVC_min) / IVC_max] × 100*


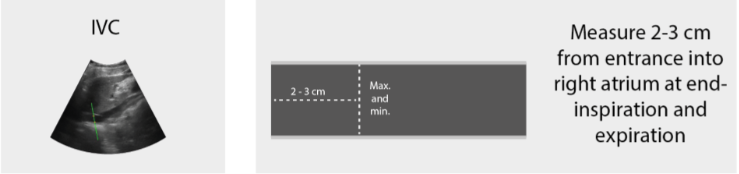

**Figure A1. Imaging and schematic outline of IVC measurement including instructions.**


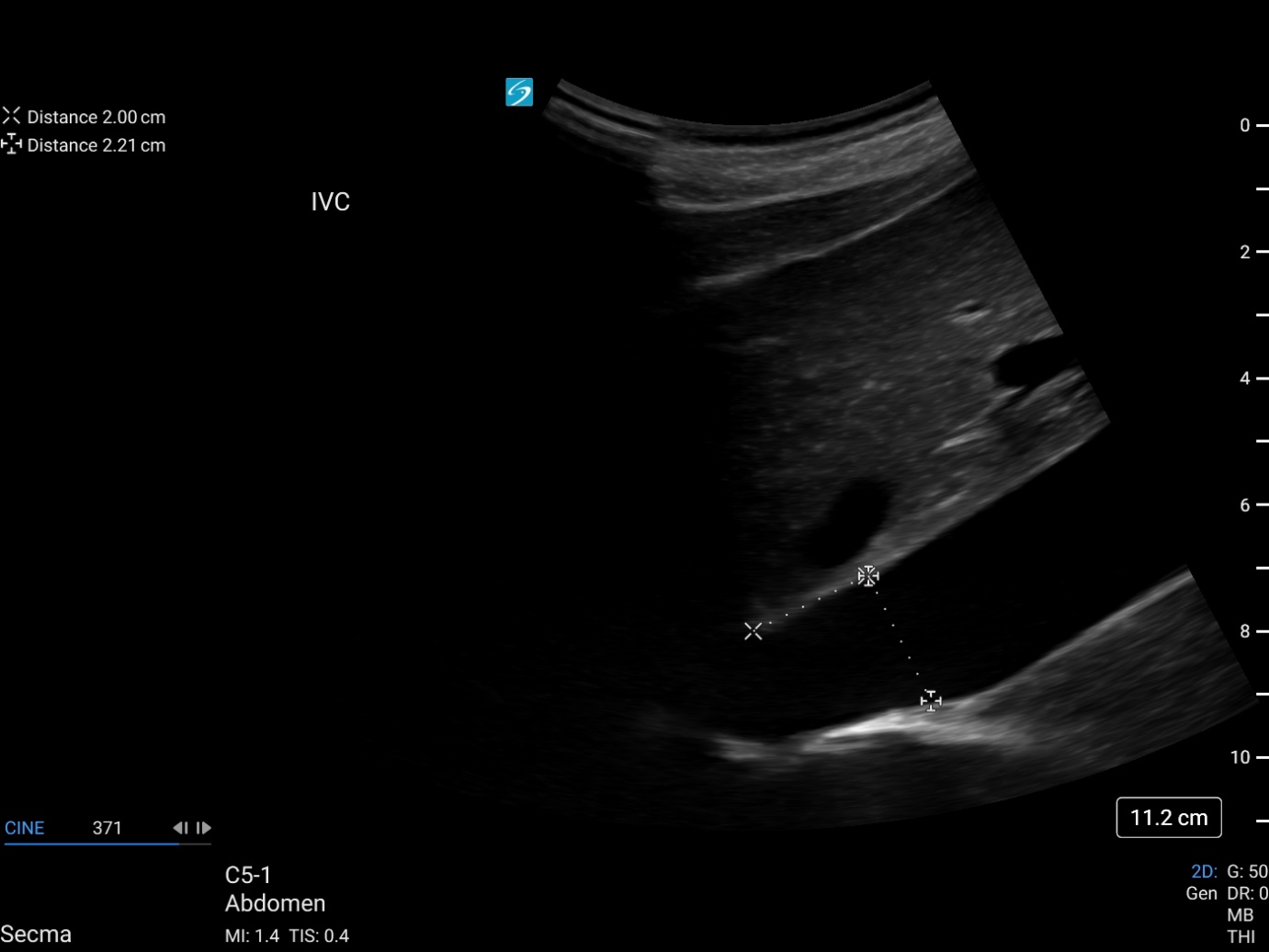


**Figure A2. Example of maximum IVC diameter measurement 2 cm from right atrial inlet.**


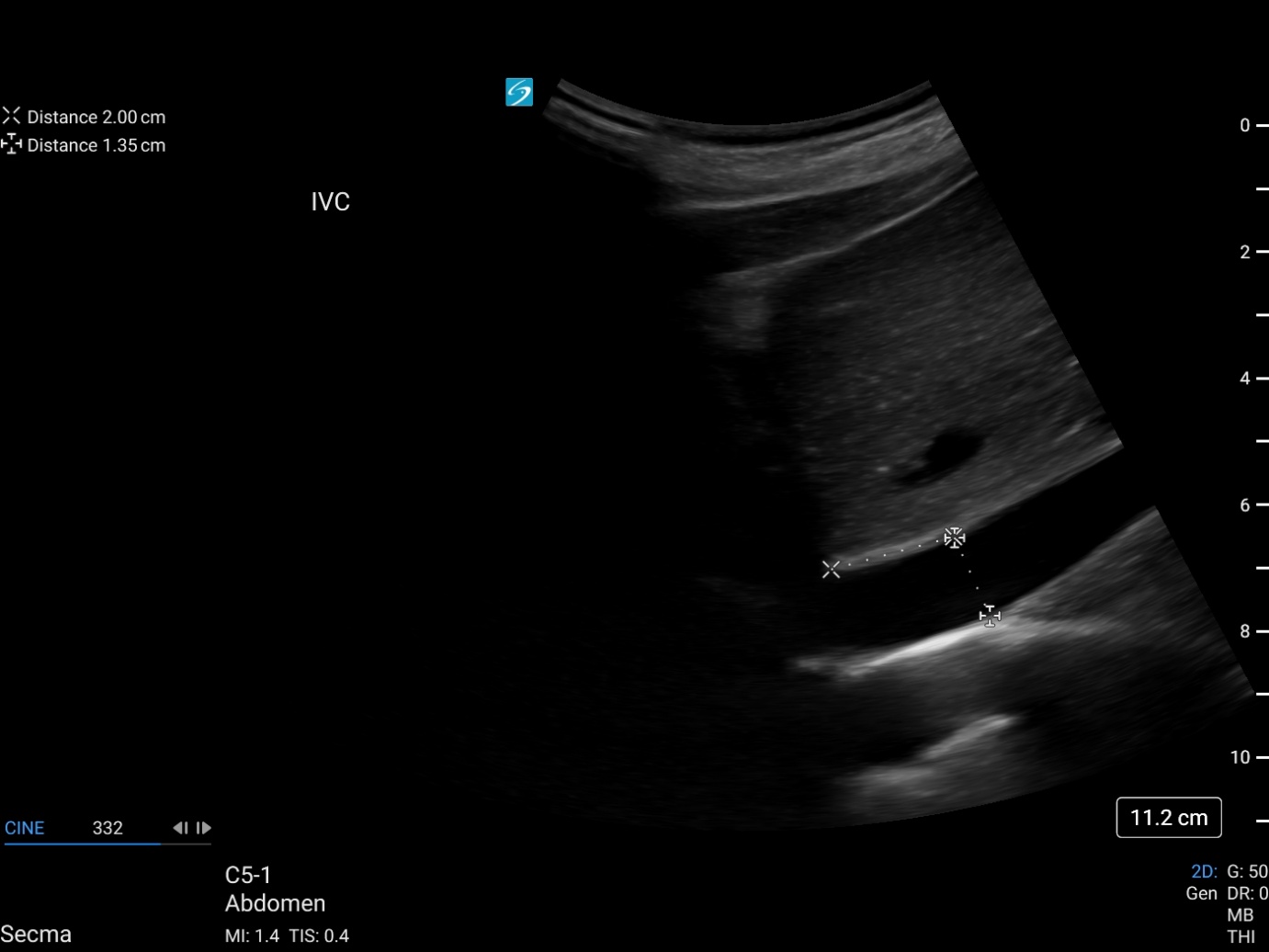


**Figure A3. Example of minimum IVC diameter measurement 2 cm from right atrial inlet.**

**VExUS: Hepatic Vein (HV) Pulsed Wave Doppler (PWD) Assessment**

| Frequency | 1-5 MHz |
| --- | --- |
| Depth | 15-20 cm (80/20 rule) |
| Probe | Curvilinear probe |
| Location | Right midaxillary line |
| Patient Position | Supine |

**Image acquisition**

1. Position the ultrasound probe in an oblique orientation within the lower intercostal space on the posterior right side of the patient, close to the bed surface.
2. Identify the inferior vena cava (IVC) and visualize the hepatic vein entering the IVC (**See** **Figure B1**).
3. Write “HV” in the apparatus to indicate the visualized structure.

**Doppler measurement**

1. Activate color Doppler mode; under normal conditions, the hepatic vein (HV) should display predominantly blue flow toward the heart (**See** **Figure B2**).
2. Position the pulsed-wave Doppler (PWD) sample volume in the hepatic vein 1–2 cm proximal to the junction of the HV with the IVC (**See** **Figure B3**).
3. Initiate PWD. Ensure the Doppler angle of insonation is <60° by adjusting the transducer position and beam steering as needed.

**Image documentation**

1. Save an image displaying a clear HV Doppler waveform for analysis (**See** **Figure B4**).
2. **Interpretation**: Interpret the waveform based on the reference image provided, which illustrates examples of normal and abnormal flow patterns (**See** **Figure B5**).


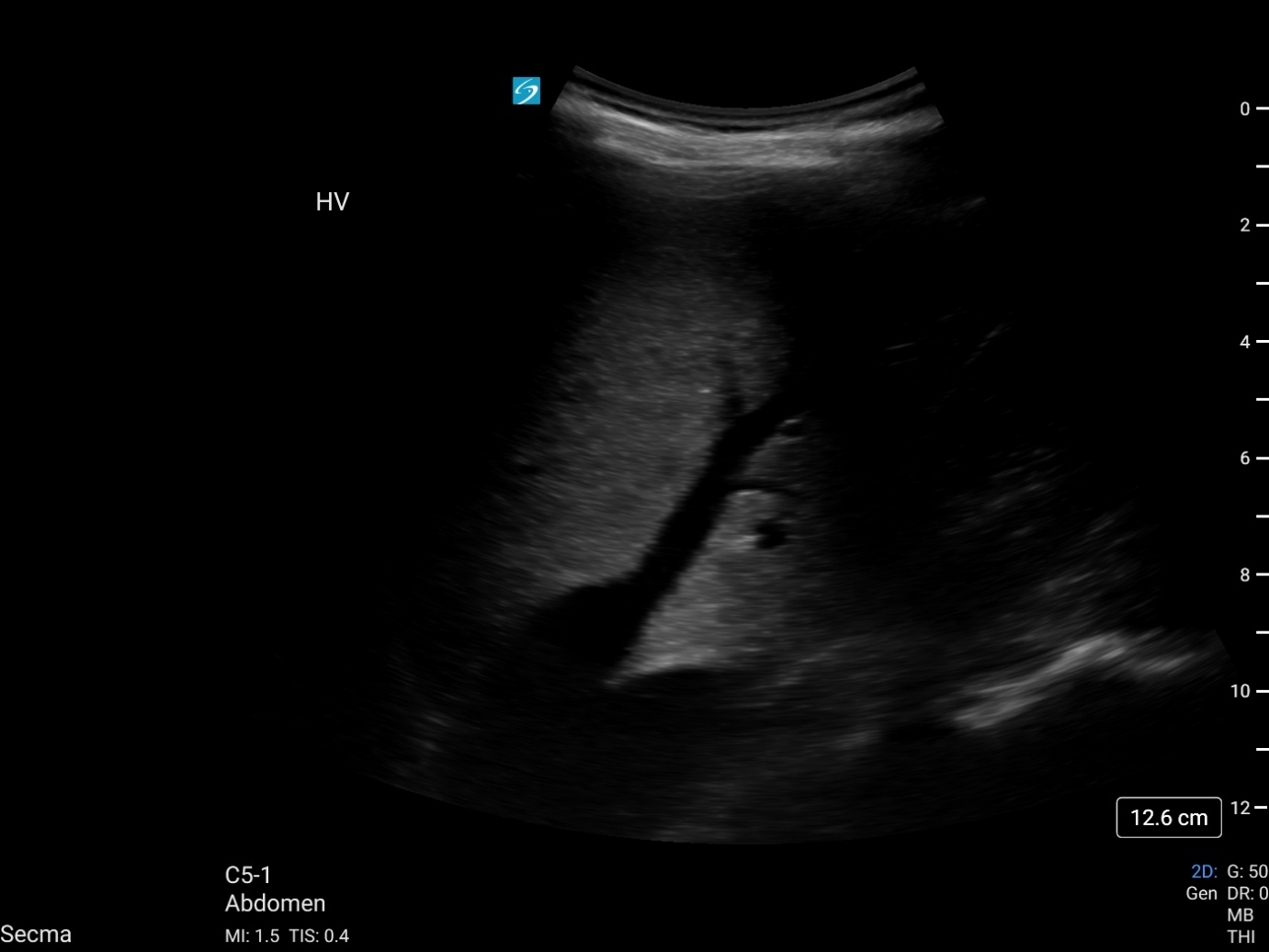


**Figure B1. Example of ultrasound image showing the hepatic vein entering the IVC.**


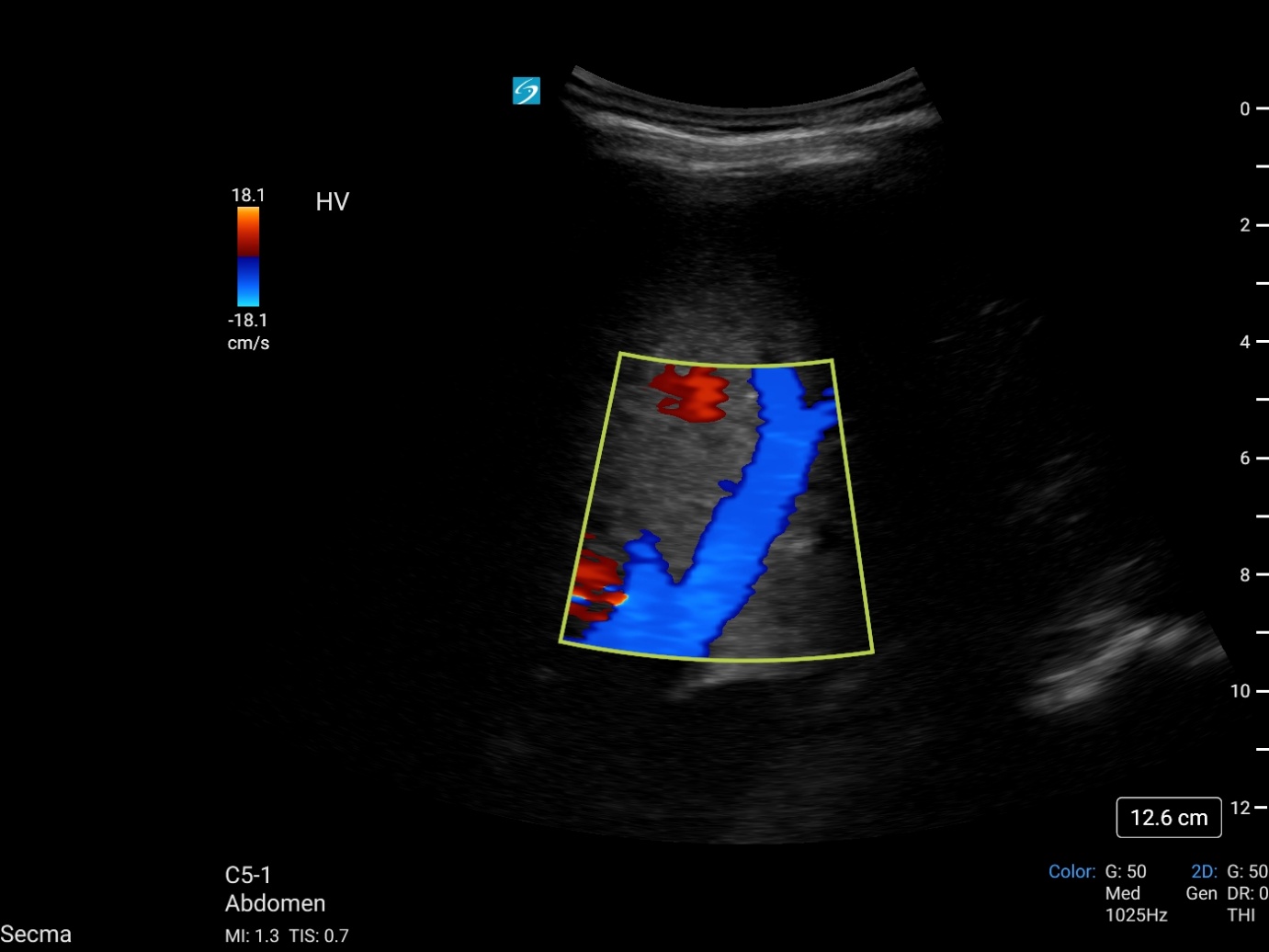


**Figure B2. Example of color Doppler showing the hepatic vein with blue colored flow.**


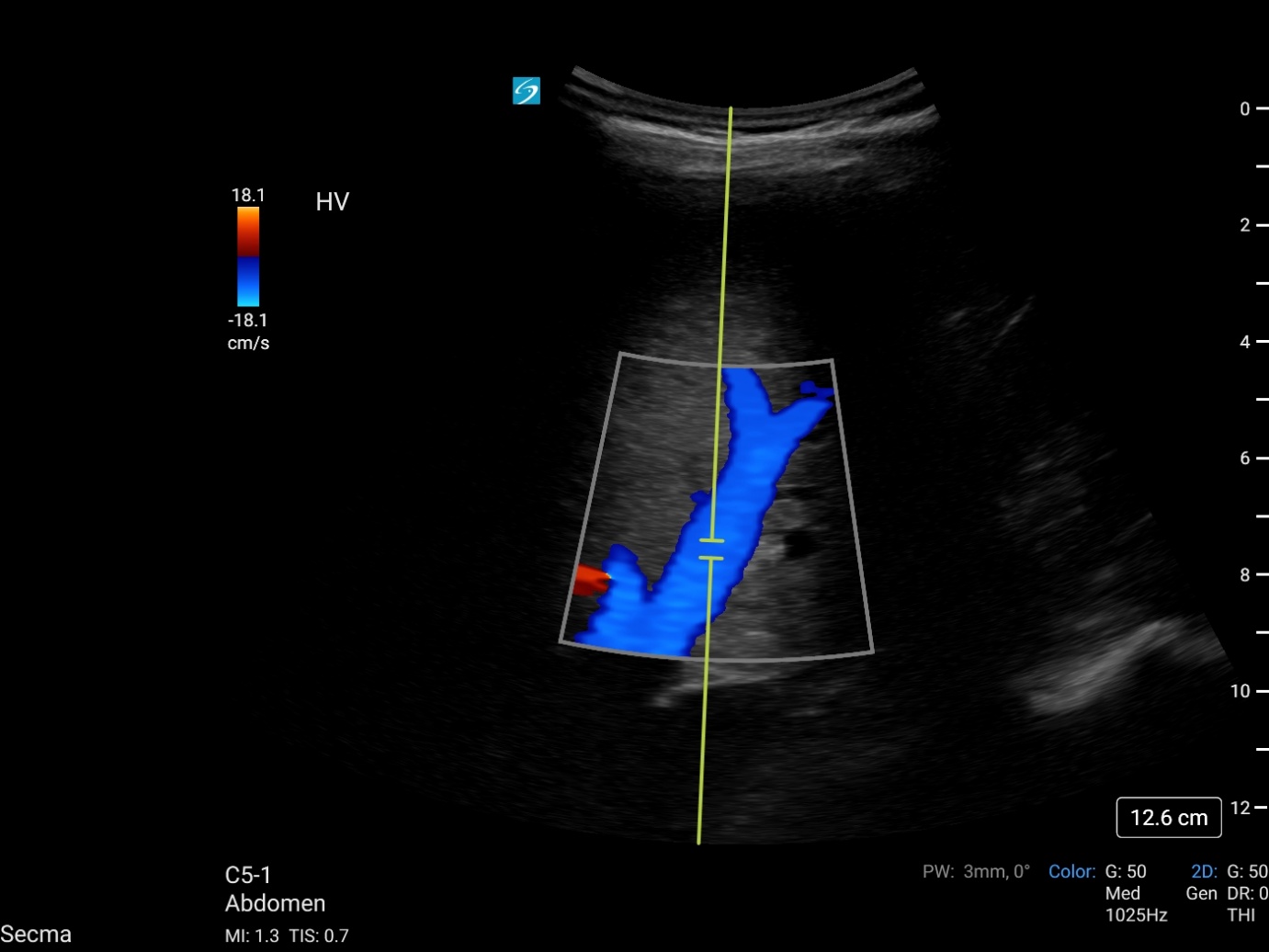


**Figure B3. Placement of Pulsed Wave Doppler pointer in the hepatic vein.**


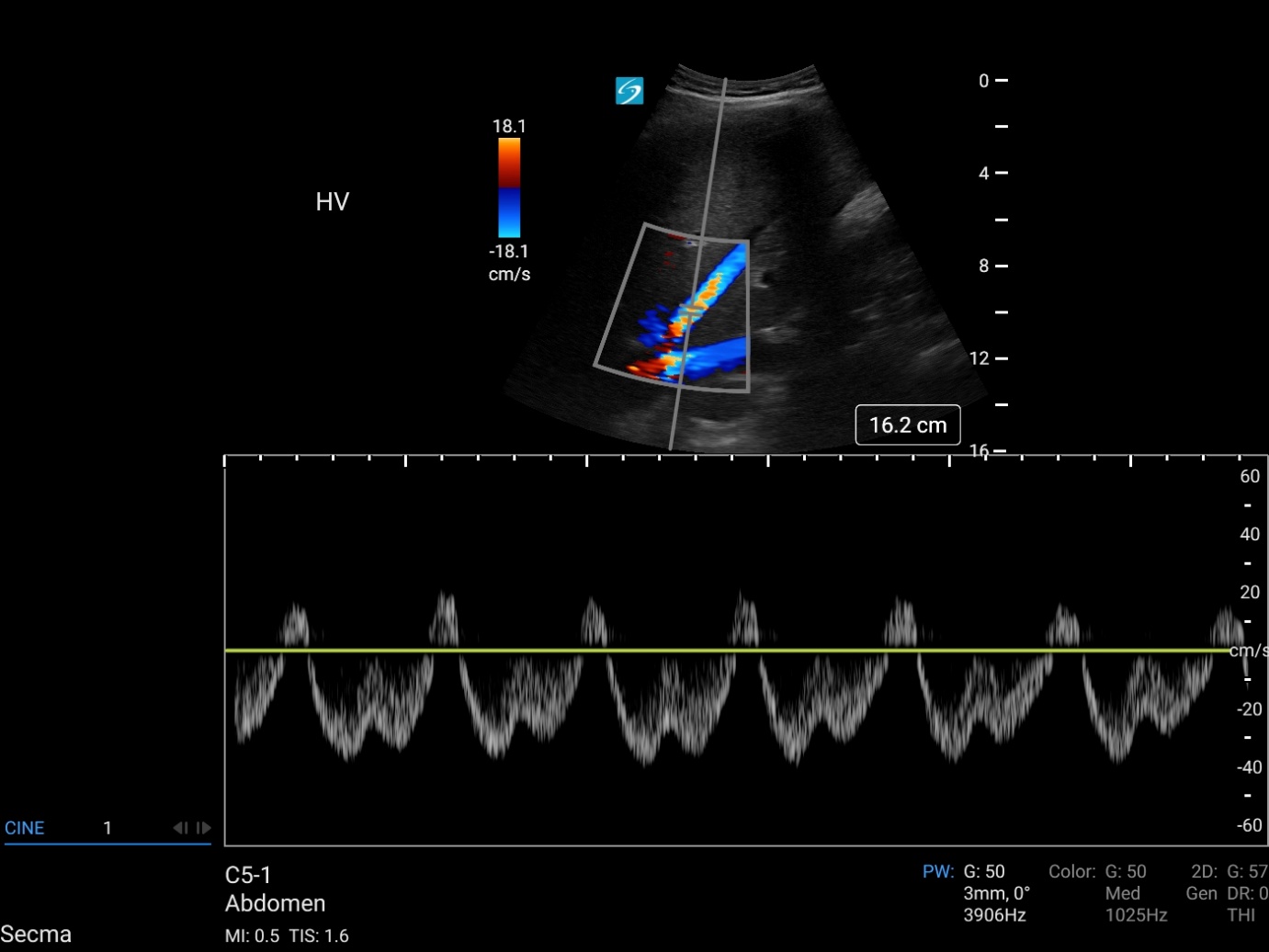


**Figure B4. Example of Pulsed Wave Doppler showing the hepatic vein flow pattern.**


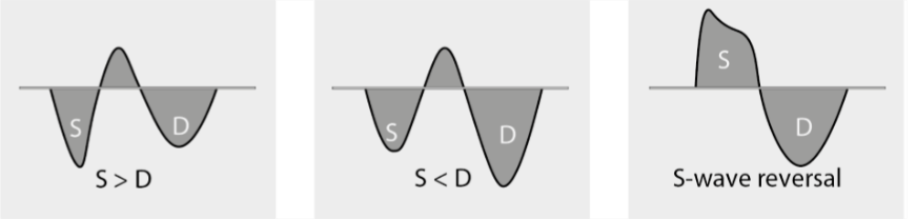
**Figure B5. Interpretation of HV PWD waveform. From left to right: normal, mildly abnormal and severely abnormal.**

**VExUS: Portal Vein (PV) PWD Assessment**

| Frequency | 1-5 MHz |
| --- | --- |
| Depth | 15-20 cm (80/20 rule) |
| Probe | Curvilinear probe |
| Location | Right midaxillary line, towards anterior axillary line |
| Patient Position | Supine |

**Image Acquisition**

1. Position the probe at the right midaxillary line with the marker oriented cranially. Adjust the probe angle as needed to avoid costal shadowing.
2. Identify the portal vein (PV) by its hyperechogenic walls, red flow on color Doppler, and monophasic waveform on pulsed-wave Doppler (PWD) (**see Figure C1**).
3. Write “PV” in the apparatus to indicate the visualized structure.

**Measurement**

1. Activate color Doppler mode; under normal conditions, the portal vein (PV) should display predominantly red flow (**see Figure C2**).
2. Place the PWD cursor within the PV, ensuring it is positioned away from the IVC and hepatic arteries. Maintain an angle of insonation less than 60 degrees(**see Figure C3**).
3. Adjust the Doppler scale as needed and freeze the image when a clear waveform is visualized (**see Figure C4**).
4. Measure the peak and trough velocities of the waveform (**see** **Figure C4**).

**Image Documentation and Interpretation**

1. Save an image that includes both velocity measurements.
2. Interpretation: Calculate the Pulsatility Index. Refer to the accompanying image for examples of normal and abnormal portal vein waveforms (**see** **Figure C5**).

*Pulsatility Index (%) = [(Peak velocity − Trough velocity) / Peak velocity] × 100*


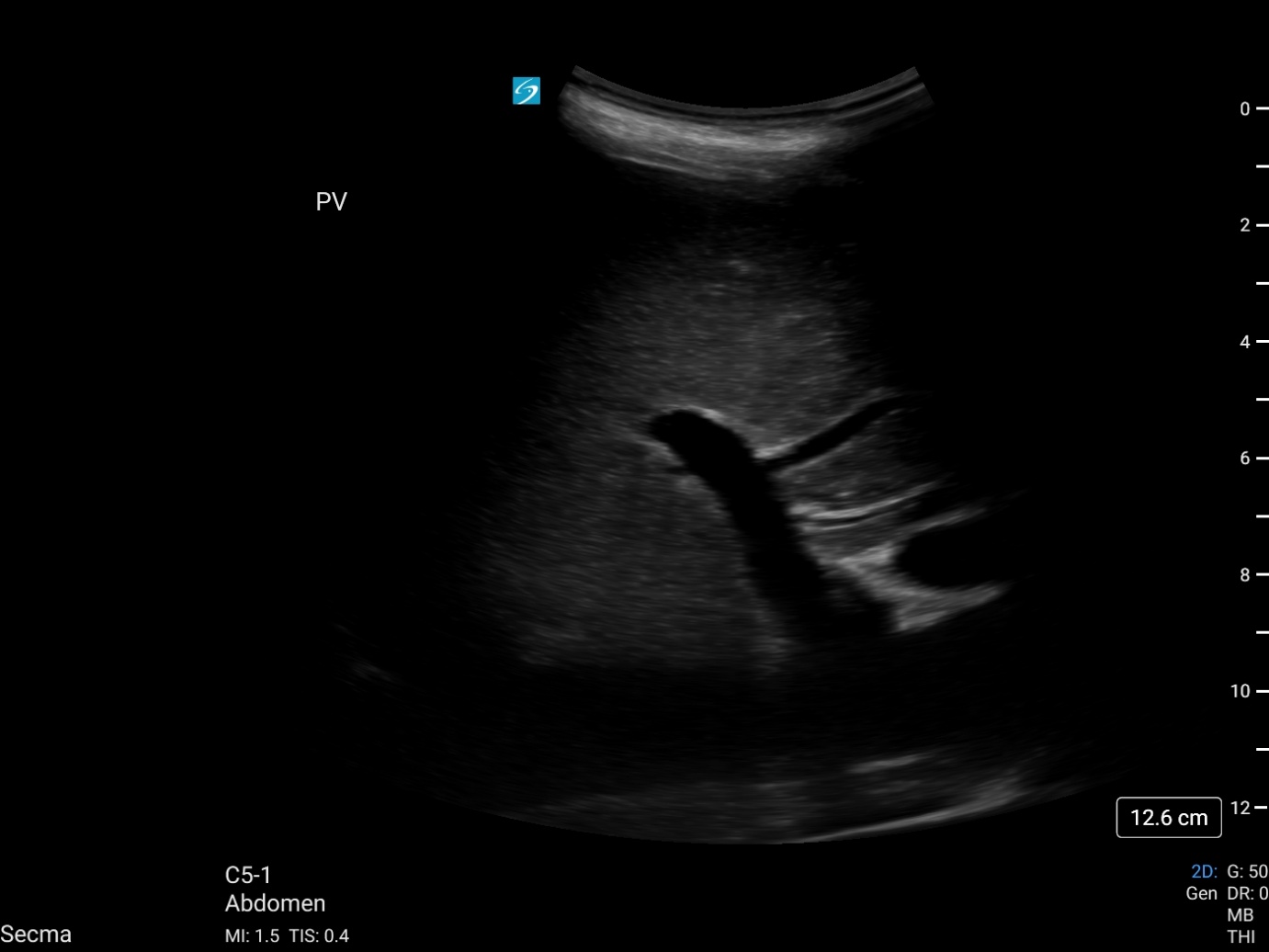


**Figure C1. Example of ultrasound image showing the portal vein.**


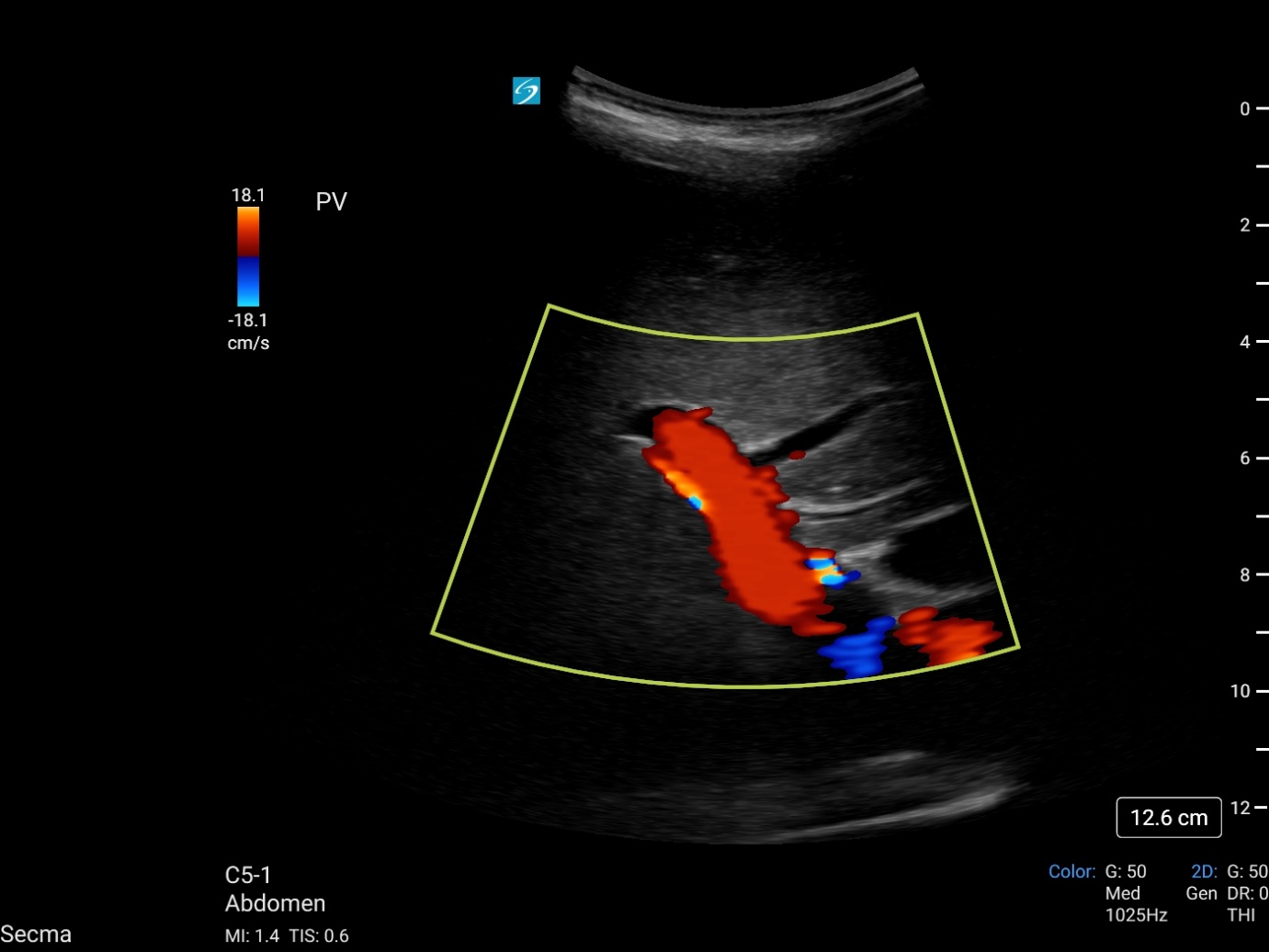


**Figure C2. Example of color Doppler showing the portal vein with red colored flow.**


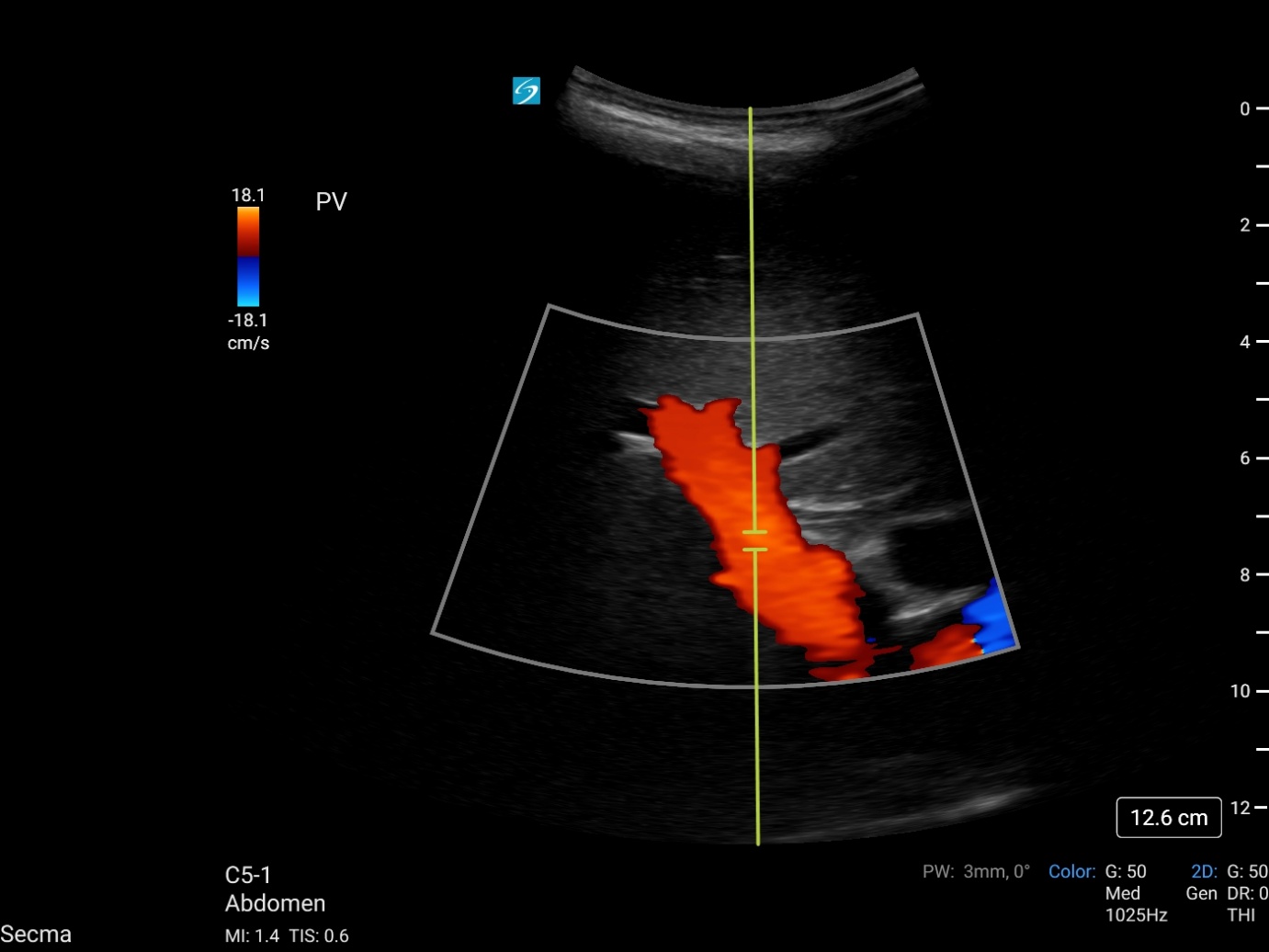


**Figure C3. Placement of Pulsed Wave Doppler pointer in the portal vein.**


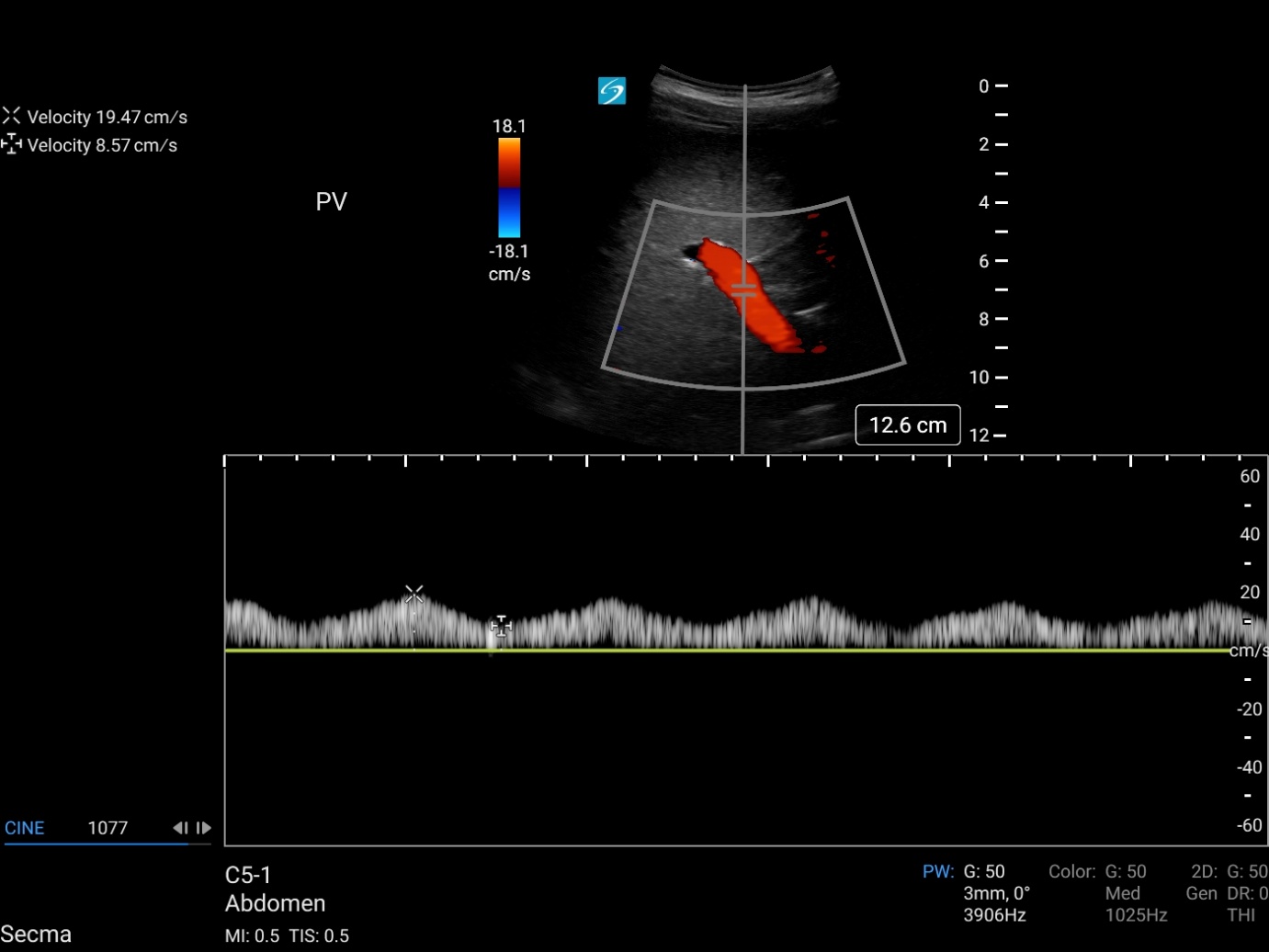


**Figure C4. Example of Pulsed Wave Doppler showing the portal vein flow pattern. Measurements of peak and trough velocities are shown in the upper left.**


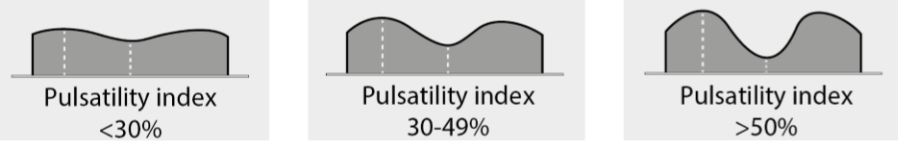
**Figure C5.  Interpretation of PV PWD waveform. From left to right: normal, mildly abnormal and severely abnormal. Pulsatility index: (Vmax − Vmin) / Vmax.**

**VExUS: Intrarenal (IRV) PWD Assessment**

| Frequency | 1-5 MHz |
| --- | --- |
| Depth | 15-20 cm (80/20 rule) |
| Probe | Curvilinear probe |
| Location | Posterior axillary line (right or left) |
| Patient Position | Supine |

**Image Acquisition**

1. Position the transducer over the posterior axillary line with the marker oriented cranially. This location is caudal to the previously assessed VExUS sites. Identify the kidney (**see Figure D1**).
2. Activate color Doppler to visualize the interlobar vessels, where both arterial and venous flow should be evident (**see Figure D2**).
3. Write “RV” in the apparatus to indicate the visualized structure.

**Measurement**

1. Position the pulsed-wave Doppler (PWD) cursor over an interlobar vessel and initiate the Doppler flow assessment. (**see Figure D3**).
2. A waveform displaying both arterial and venous components should be visible (**see** **Figure D4**).
3. Perform measurements on the arterial component of the flow pattern to measure the renal resistive index (RRI): peak systolic flow velocity and end-diastolic flow velocity (**see Figure D4**).
4. Perform measurements on the venous component of the flow pattern to measure the venous impedance index (VII): maximal flow velocity and minimal flow velocity within one cardiac cycle (**see Figure D4**).

**Image Documentation and Interpretation**

1. Save an image containing the PWD waveform of the intrarenal vein (IRV).
2. Interpretation: Refer to the different venous flow patterns **in Figure D5** for distinguishing features of normal and abnormal renal venous Doppler flow.
3. Calculate RRI: (peak systolic flow velocity – end-diastolic flow velocity) / peak systolic flow velocity. *Normal range = 0.5-0.7.*
4. Calculate VII: (maximal flow velocity – minimal flow velocity) / maximal flow velocity. *Lower VII indicates less congestion.*


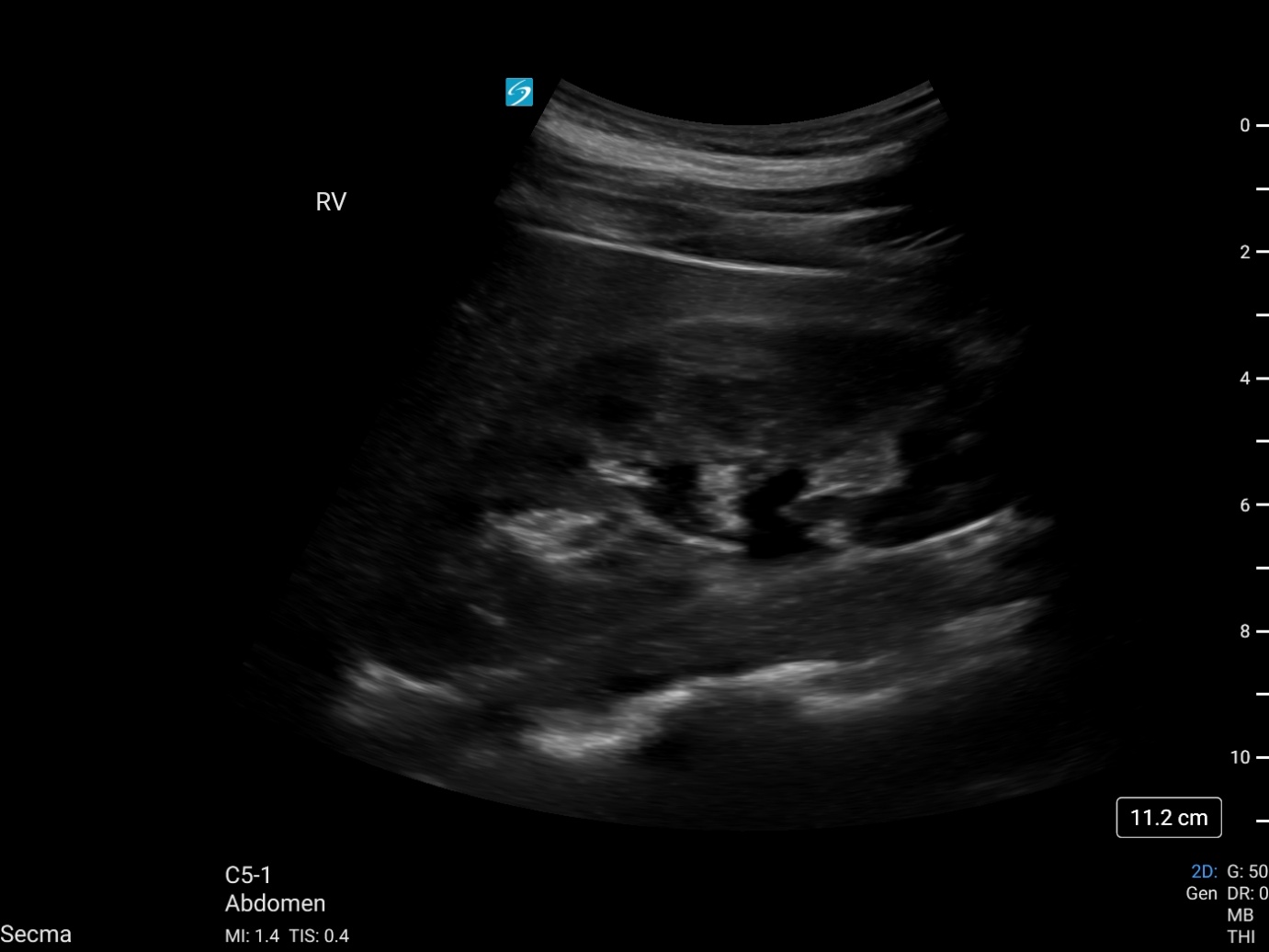


**Figure D1. Example of ultrasound image showing the right kidney.**


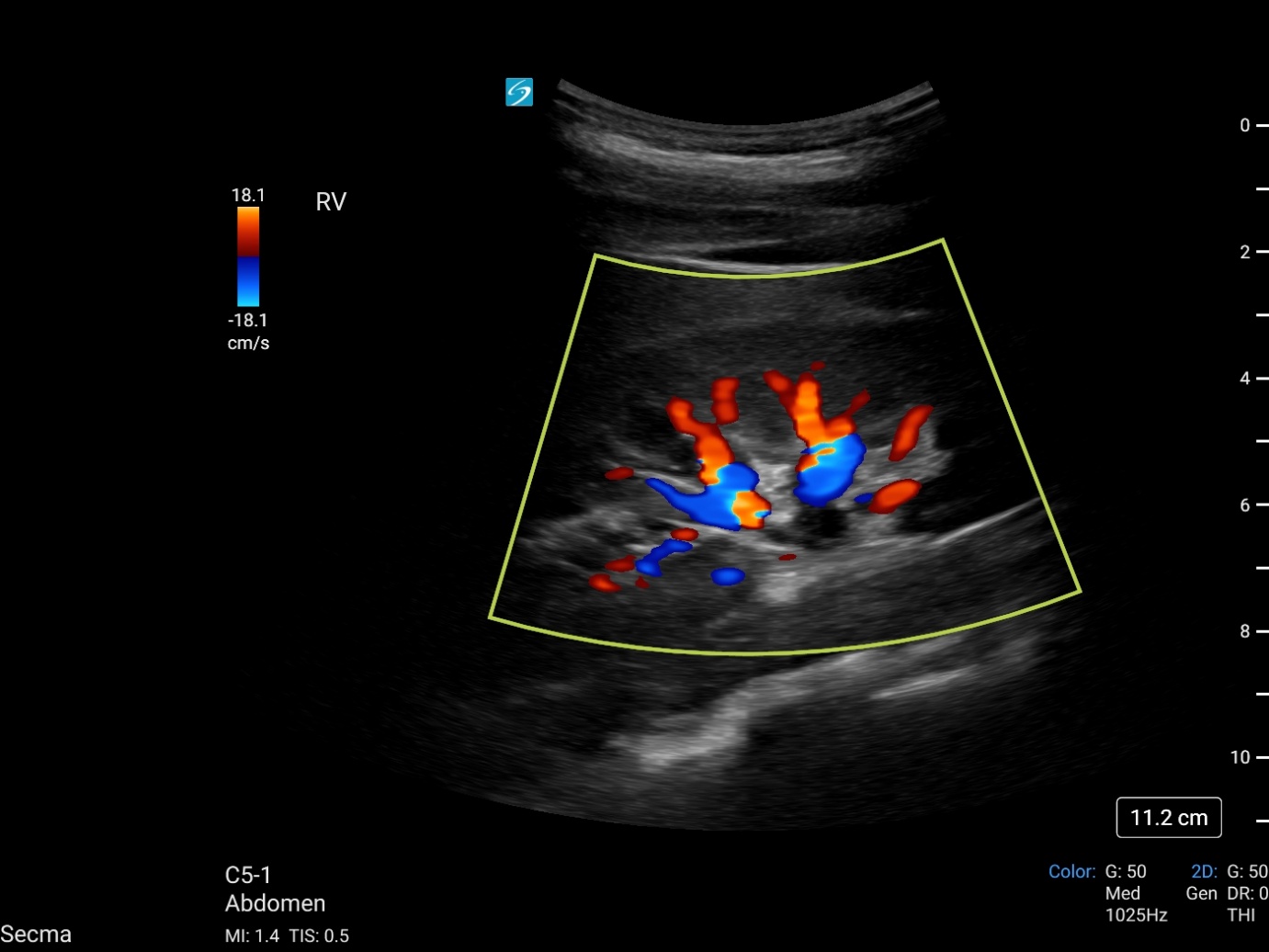


**Figure D2. Example of color Doppler showing the veins and arteries within the kidney.**


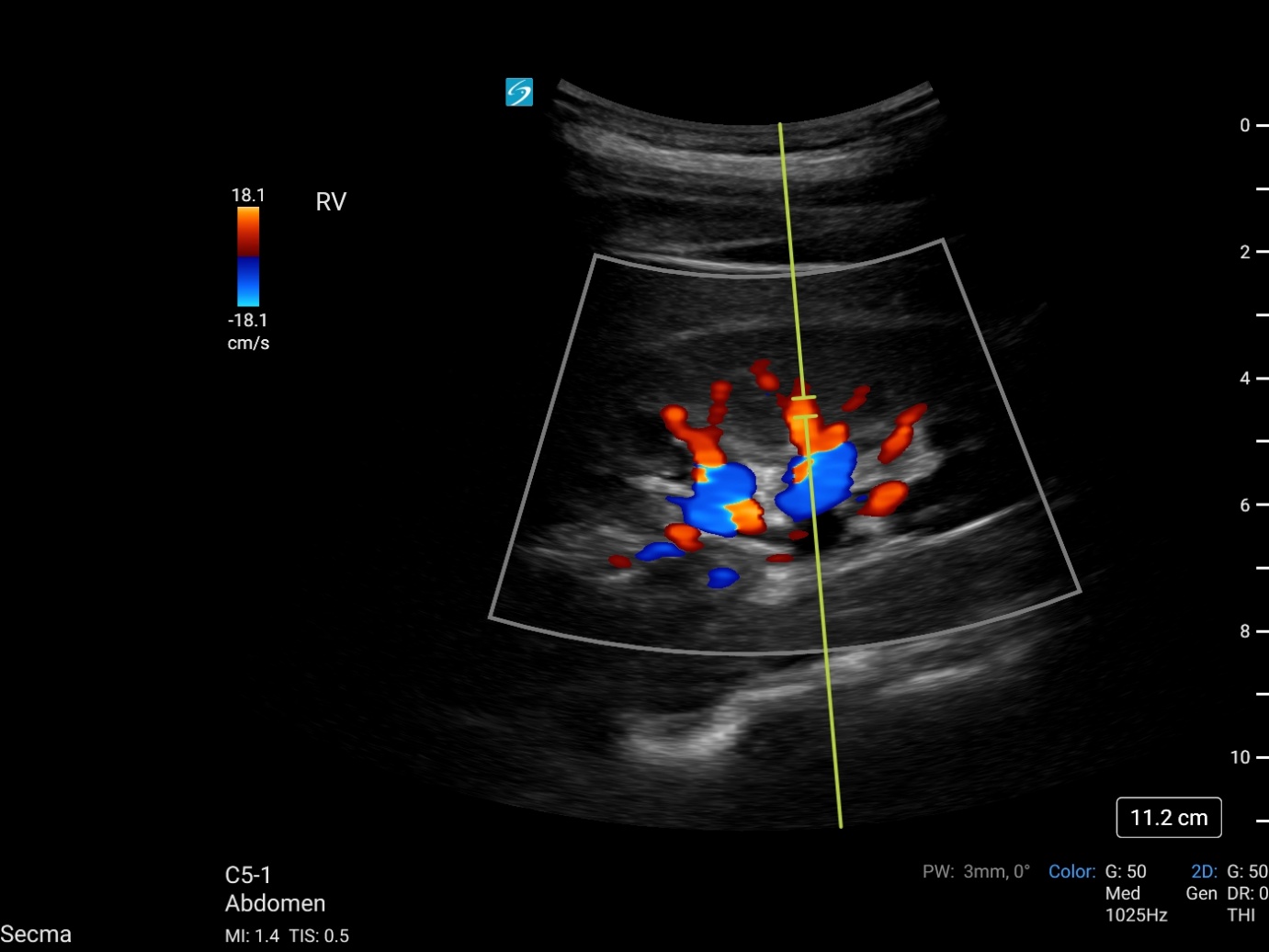


**Figure D3. Placement of Pulsed Wave Doppler pointer at the height of the interlobular arteries and veins of the kidney.**


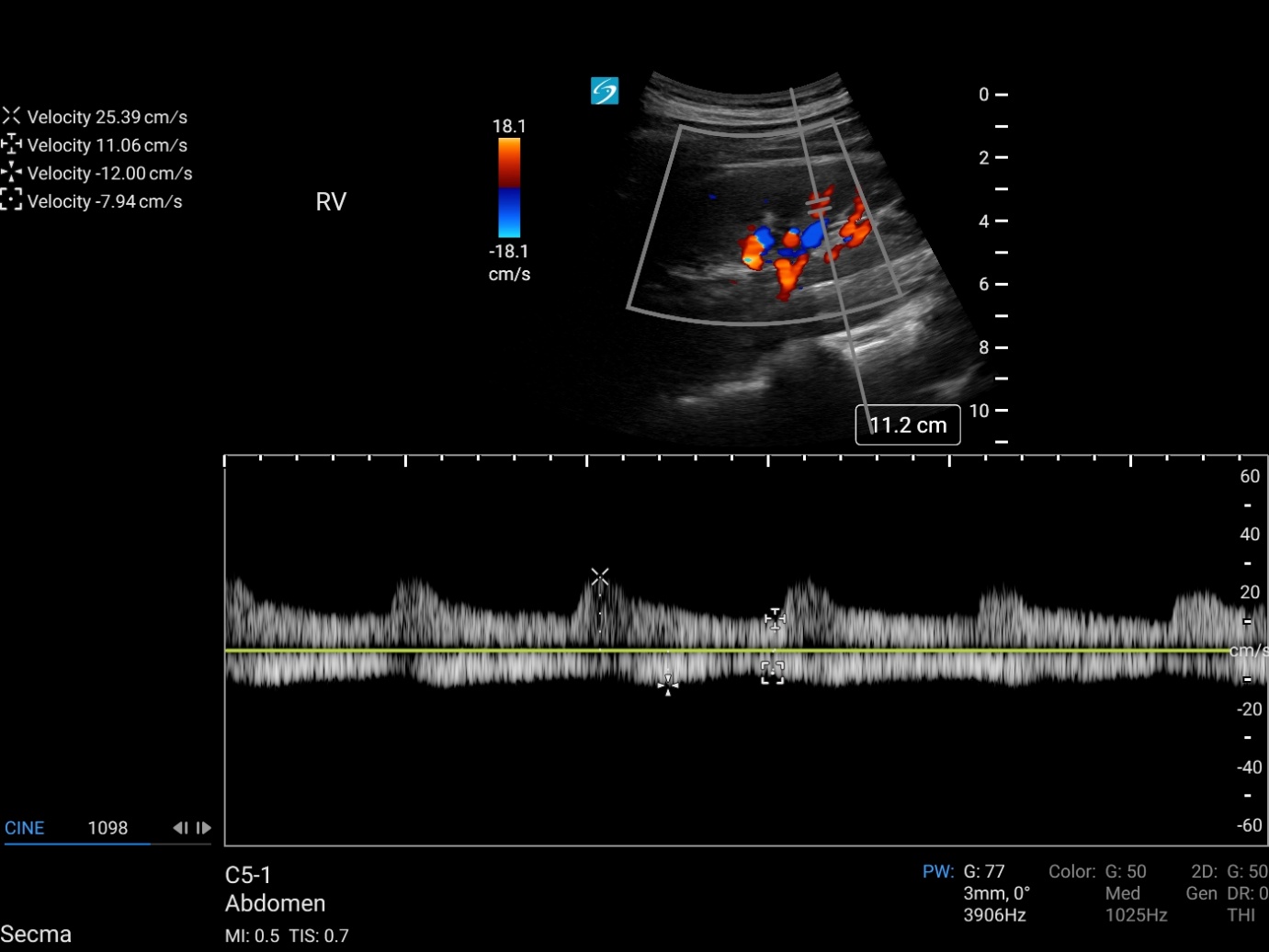


**Figure D4. Example of Pulsed Wave Doppler showing the renal arterial and venous flow pattern. Measurements of the arterial waveform (peak systolic flow velocity and end-diastolic flow velocity) and the venous waveform (maximal and minimal flow velocity) are shown in the upper left.**


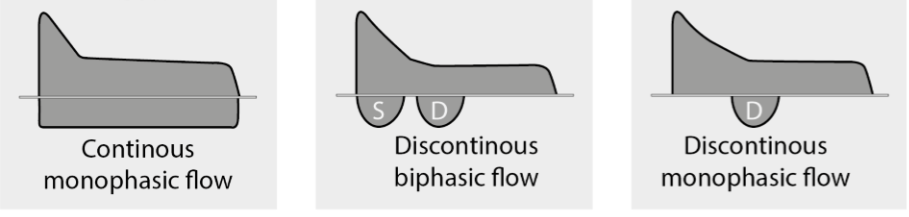


**Figure D5. Interpretation of intrarenal vein (IRV) pulsed-wave Doppler (PWD) waveforms. From left to right: normal pattern, mildly abnormal pattern, and severely abnormal pattern.**

**VExUS interpretation:**

Grade 0: IVC <2cm = NO Congestion

Grade 1: IVC >2cm with any combo of Normal or Mildly Abnormal Patterns = MILD Congestion

Grade 2: IVC >2cm and ONE severely Abnormal Pattern = MODERATE Congestion

Grade 3: IVC >2cm and >2 Severely Abnormal Patterns = SEVERE Congestion

**Bedside Lung Ultrasound in Emergencies (BLUE): Assessment**

| Frequency | C1-5 |
| --- | --- |
| Depth | 16cm or more |
| Probe | Curvilinear |
| Location | BLUE points |
| Patient Position | Supine |

**Image Acquisition:**

1. Image the lung at the six standardized points shown **in Figure E1**.
2. Write “L1”-“L3” or “R1” - “R3” in the apparatus to indicate the visualized BLUE point.
3. Assess lung sliding to evaluate pleural movement and detect pleural edema.

**Measurement:**

1. Position the probe perpendicular to the pleura to visualize sharp, thin, hyperechogenic pleural lines between two rib spaces.
2. Identify A-lines (horizontal reverberation artifacts indicating normal aeration) and B-lines (vertical, hyperechogenic lines extending ≥15 cm to the bottom of the screen, indicating increased lung density) (**see Figure E2 and E3**).

**Image Documentation and Interpretation:**

1. Record and save a labeled video clip of each lung scanning point.
2. Assess for B-lines:
   1. No B-lines: no pulmonary edema.
   2. Unilateral B-lines: pulmonary edema unlikely.
   3. Bilateral B-lines: pulmonary edema likely.


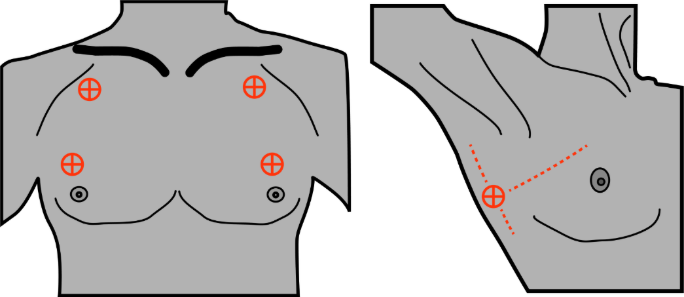


**Figure E1. Locations of Bedside Lung Ultrasound in Emergencies (BLUE) protocol scanning points:**
***Right side — Subclavicular (R1), Supra-areolar (R2), and Posterolateral (R3);***
***Left side — Corresponding points labeled L1, L2, and L3.***


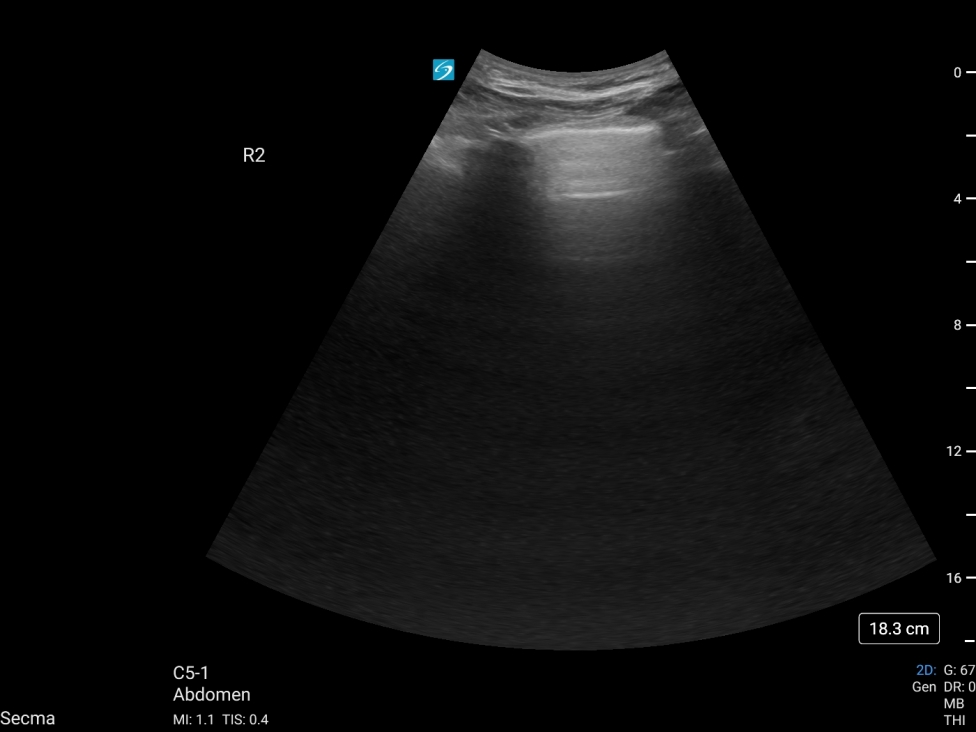


**Figure E2. Ultrasound image of one of the BLUE locations (R2), showcasing the hyperechoic pleura with horizontal A-lines.**

**
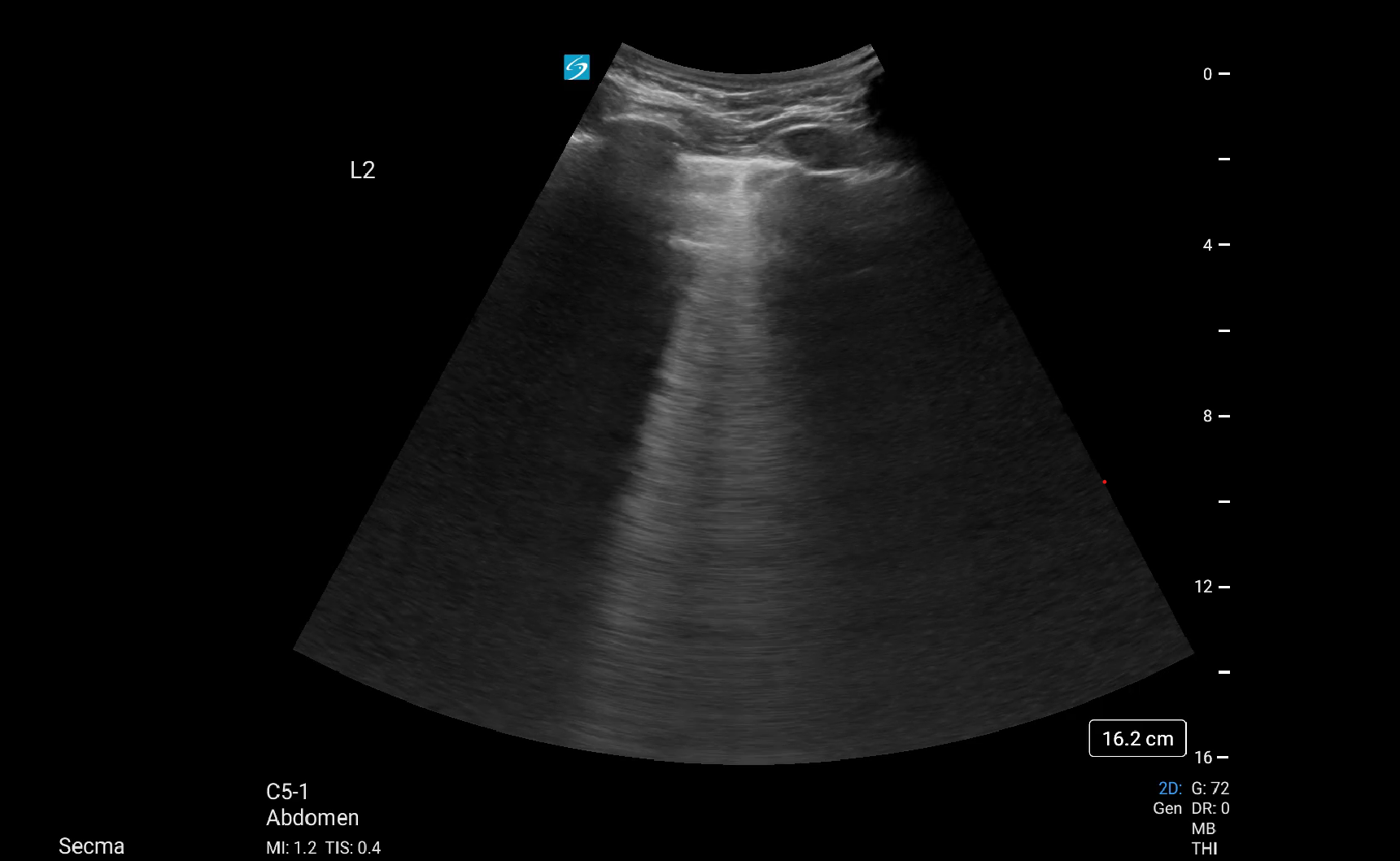
**

**Figure E3. Ultrasound image of one of the BLUE locations (L2), showcasing vertical B-lines.**

**End of Examination:**

1. Save all imaging to the research drive
2. Document all imaging interpretation in REDCap FLUIDS database, including:
    a. Patient position
    b. Mechanical ventilation status (yes/no)
    c. Any technical difficulties encountered for feasibility analysis
