# Supplementary material for: FLUid management and InDividualized resuscitation in Sepsis (FLUIDS)—A Study protocol for a single-centre, open-label, randomized clinical trial
Source: PLoS One. 2025 Dec 19;20(12):e0338504. doi: 10.1371/journal.pone.0338504 (PMC12716701; doi:10.1371/journal.pone.0338504)
Supplement: S2 File — Supplementary material containing the template participant information sheet and consent form in the study, provided in Dutch. (PDF) [file pone.0338504.s002.pdf]

Proefpersoneninformatie voor deelname aan medisch-wetenschappelijk onderzoek

FLUIDS – Gepersonaliseerde behandeling voor patiënten met sepsis op de spoedeisende hulp (SEH)

Officiële titel: Vocht management en geïndividualiseerde resuscitatie in sepsis

Inleiding

Geachte heer/mevrouw,

U ontvangt deze brief omdat u behandeld bent met een infuusvloeistof en mogelijk bloeddruk-verhogende medicatie voor sepsis. *In de volksmond wordt dit bloedvergiftiging genoemd, in deze informatiebrief spreken wij van sepsis*. Met deze informatiebrief willen we u op de hoogte brengen over het medisch-wetenschappelijk onderzoek waar u op dit moment aan mee doet. We hebben u eerder niet zelf om toestemming kunnen vragen omdat u gedurende deze behandeling ernstig ziek was. Daarom hebben wij, indien mogelijk, uw naaste destijds een samenvattende brief gegeven op de Spoedeisende Hulp, waarin zij toestemming hebben gegeven om het onderzoek voort te zetten. Wij willen u nu middels deze brief alsnog om toestemming vragen om mee te blijven doen aan dit onderzoek. Meedoen is vrijwillig. Voordat u beslist of u mee wilt blijven doen met dit onderzoek, zullen wij u eerst meer uitleg geven over dit onderzoek, wat het voor u betekent en wat de voor- en nadelen zijn als u mee doet met dit onderzoek. Wilt u de informatie doorlezen en beslissen of u wilt meedoen? Als u wilt meedoen, kunt u het formulier invullen dat u vindt in **bijlage D**.

Copy

Stel uw vragen

U kunt uw beslissing nemen aan de hand van de informatie die u in deze informatiebrief vindt. Daarnaast raden we u aan om dit te doen:
- Stel vragen aan de onderzoeker die u deze informatie geeft.
- Praat met uw partner, familie of vrienden over dit onderzoek.
- Stel vragen aan de onafhankelijk deskundige, dr. B.D. Westenbrink.
- Lees de informatie op www.rijksoverheid.nl/mensenonderzoek.

1. Algemene informatie

Het Universitair Medisch Centrum Groningen heeft dit onderzoek opgezet. Hierna noemen we het Universitair Medisch Centrum Groningen steeds de ‘opdrachtgever’. Onderzoekers voeren het onderzoek uit in het Universitair Medisch Centrum Groningen. Dit kunnen artsen, onderzoekers en onderzoeksverpleegkundigen zijn.

Voor dit onderzoek zijn in totaal 174 proefpersonen nodig. Het onderzoek wordt mede mogelijk gemaakt en betaald door het Universitair Medisch Centrum Groningen. De medisch-ethische toetsingscommissie Regionale Toetsingscie Patientgebonden Onderzoek (RTPO) Leeuwarden heeft dit onderzoek goedgekeurd.

2. Wat is het doel van het onderzoek?

Sepsis is een ontstekingsreactie van het lichaam op een infectie, waarbij vaak sprake is van een te lage bloeddruk. Deze lage bloeddruk wordt in eerste instantie behandeld met infuusvloeistof waarbij het soms nodig is om aanvullend bloeddruk verhogende medicatie te starten. Het is echter niet duidelijk hoeveel infuusvloeistof gegeven moet worden, voordat gestart moet worden met bloeddruk verhogende medicatie.
Voor dit onderzoek worden deelnemers door loting ingedeeld in de onderzoeksgroep die een gepersonaliseerde behandelplan ontvangt of in de groep die standaardzorg voor de lage bloeddruk door sepsis ontvangt. We vergelijken de werking het gepersonaliseerde behandelplan met de standaardzorg voor sepsis, zoals dat nu al gebruikt wordt.
In het gepersonaliseerde behandelplan meten wij het bloedvolume dat uw hart rondpompt. Dit doen wij met de monitor naast uw bed. Dit is de Baxter Starling monitor welke via elektroden, die op uw borst geplakt worden zoals bij het maken van een hartfilmpje, is verbonden met u.
Aan de hand van deze meting bepalen wij of- en hoeveel infuusvloeistof u krijgt of dat u bloeddruk- verhogende medicijnen krijgt.
Dit onderzoek heeft geen invloed op de rest van zorg die gegeven wordt voor sepsis, zoals antibiotica of bijvoorbeeld een opname op de Intensive Care (IC) als dan nodig mocht zijn.

Copy

3. Wat is de achtergrond van het onderzoek?

Sepsis is de meest voorkomende doodsoorzaak door infecties wereldwijd. Het is een ernstige aandoening waarbij de infectie resulteert in een algemene ontstekingsreactie van het gehele lichaam. Hierdoor kunnen organen minder gaan werken of helemaal uitvallen. Dit kan in sommige gevallen resulteren in overlijden. In het begin van sepsis verandert er veel in de bloedvaten, wat kan leiden tot een te lage bloeddruk. Als er hierdoor te weinig doorbloeding van de organen is, noemen we dit een shock.

De behandeling voor sepsis bestaat uit het geven van vocht via het infuus (infusievloeistof) en medicijnen om de bloeddruk te verhogen. Verder wordt er antibiotica gegeven en kan het nodig zijn om op de intensive care (IC) opgenomen te worden. De huidige aanpak past niet altijd goed bij elke patiënt, omdat sepsis bij iedereen anders kan verlopen. Het gevolg kan zijn dat de shock te lang duurt of dat het lichaam te veel vocht krijgt. We denken dat een meer persoonlijke aanpak betere resultaten kan geven.

Met behulp van de Starling monitor kunnen artsen het gepersonaliseerd behandelplan voor elke patiënt maken. Dit apparaat kan namelijk op elk moment informatie geven over de hoeveelheid bloed die door het hart pompt van patiënten. Om dit te doen, wordt het apparaat verbonden met de patiënt door vier plakkers op de borst te plaatsen. Het apparaat gebruikt

elektrische golven om de effecten in het hart en de bloedvaten te meten. Op basis van deze metingen kunnen artsen de vochttoediening en bloeddrukverhogende medicatie bijstellen.

We willen onderzoeken of de Starling monitor ons kan helpen om te bepalen hoeveel infuusvloeistof we sepsispatiënten moeten geven in de eerste drie uur nadat ze op de Spoedeisende Hulp zijn opgenomen. Verder onderzoeken wij ook wanneer het verstandig is om te starten met bloeddruk verhogende medicijnen, mocht dat nodig zijn. Wij verwachten dat dit de behandeling verbetert en ervoor zorgt dat patiënten sneller beter worden, terwijl het risico op te veel vocht beperkt blijft.

4. Hoe verliep het onderzoek?

Hoelang duurde het onderzoek?
Deed u mee met het onderzoek? Dan heeft in totaal twee dagen geduurd.

Stap 1: was u geschikt om mee te doen?
We hebben eerst uitgezocht of u geschikt bent om mee te doen. Daarom heeft de onderzoeker een aantal gegevens verzameld vóórdat u op de Spoedeisende Hulp arriveerde:

- Gegevens van de huisarts en/of ambulance zoals lichamelijk onderzoek. De huisarts of ambulance medewerker luistert bijvoorbeeld naar uw hart en longen en meet uw bloeddruk en hartslag.
- Of er een verdenking op een infectie en/of sepsis was door uw behandelend arts.
- Onderzoek naar uw algemene en medische geschiedenis.

Copy

Stap 2: de behandeling
We behandelden u voor drie uur gedurende uw SEH-verblijf, met het gepersonaliseerde of het standaard behandelplan. We verzamelden daarnaast nog enkele standaard zorggegevens en metingen tot 48 uur (twee dagen) na uw SEH-bezoek. Deze zorggegevens en metingen houden onder andere de vochtbalans, echo-metingen en lichamelijk onderzoek in. Daarna hebben we enkel op dag 7 en dag 30 gegevens uit uw medisch dossier opgevraagd.

Na de eerste drie uur van het onderzoek heeft de arts naar eigen inzicht verdere vochttoediening toegediend in beide groepen. De overige behandeling, zoals antibiotica of opname op de Intensive Care (IC), is tijdens dit onderzoek niet beïnvloed geweest.

Voor dit onderzoek hebben wij twee groepen gemaakt:

- Groep 1. De mensen in deze groep kregen het gepersonaliseerde behandelplan.
- Groep 2. De mensen in deze groep kregen het standaardbehandelplan.

Loting heeft bepaald welke behandeling u heeft gekregen. Dit is bekend gemaakt aan zowel u, de onderzoekers als het behandelteam ten tijde van aankomst op de Spoedeisende Hulp.

Stap 3: onderzoeken en metingen

Voor het onderzoek was het nodig dat we u behandelden volgens het gepersonaliseerde of standaard behandelplan direct bij binnenkomst op de Spoedeisende Hulp (SEH). Uw actieve deelname duurde maximaal 48 uur na aankomst op de SEH. U hoeft niet extra op bezoek te komen voor de studie. We volgden u daarna via dossieronderzoek op tot 30 dagen na uw SEH bezoek.

Wat is er anders dan bij gewone zorg?

Enkel als u in groep 1 (*gepersonaliseerd behandelplan*) bent ingedeeld:

- De Starling monitor: De onderzoeker heeft u bij binnenkomst op de SEH op deze monitor aangesloten. Hierbij werden er vier plakkers op uw borst geplaatst. Uw hart- en vaatfunctie werden steeds gemeten voor drie uur lang. Dit doen we om het effect van de vochttherapie op uw hartfunctie doorgaans te meten. Hiermee bepalen we ter plekke wat de volgende stap in het behandelplan is. U heeft de plakkers niet mogen verwijderen. De onderzoeker heeft de plakkers na drie uur verwijderd.

Als u zowel in groep 1 als groep 2 (*gepersonaliseerd en standaard behandelplan*) bent ingedeeld:

- Echo (echografische) metingen: De onderzoeker heeft echo metingen verricht om het vocht in uw lichaam te meten. De onderzoeker deed deze metingen op twee momenten: drie uur nadat u op de SEH was aangekomen, en nog een keer na 24 uur. Met deze onderzoeken hebben we het effect van de behandelingen op de SEH gemeten.

Copy

5. Welke afspraken maken we met u?

Voor deelname aan het onderzoek onderging u alleen de metingen tijdens de eerste 48 uur (2 dagen). Welke metingen voor uw groep werden uitgevoerd, staan benoemd in paragraaf 4. U hoeft niet extra terug te komen naar het ziekenhuis. Alle verdere handelingen vallen onder de standaardzorg voor sepsis.

Het is belangrijk dat u contact opneemt met de onderzoeker als u niet meer mee wilt doen aan het onderzoek of als contactgegevens gewijzigd zijn.

6. Van welke nadelige effecten of ongemakken kunt u last krijgen?

Groep 1: Het gepersonaliseerde behandelplan kan nadelige effecten geven. De risico's van het gebruik van de Starling monitor zijn minimaal. Een mogelijk nadelig effect is lichte huidirritatie door de kleefstof. Op basis van metingen door het apparaat, krijgt de behandelaar advies over de hoeveelheid infuusvloeistof die gegeven moet worden. Tijdens dit onderzoek bent u voor drie uur aangesloten geweest aan de Starling monitor. De behandelaar blijft altijd eindverantwoordelijk.

Groep 2: Het standaard behandelplan kan nadelige effecten geven. Tijdens het standaard behandelplan zijn er geen metingen verricht van het bloedvolume dat uw hart rondpompt. De infuusvloeistof en mogelijk bloeddruk verhogende middelen zijn bepaald volgens de huidige

richtlijnen en de expertise van uw behandeld arts. We hebben dan geen direct zicht op de effecten van de infuustoediening. De behandelaar blijft altijd eindverantwoordelijk.

Om de risico's bij de behandelplannen te verlagen wordt u goed gemonitord en er kan een minimale en maximale hoeveelheid infuusvloeistof worden gegeven. Verder heeft de onderzoeker gedurende twee dagen nog extra metingen gedaan. Deze metingen/handelingen zullen u enkel wat extra tijd kosten.

Meer informatie over het gepersonaliseerde behandelplan en extra onderzoeken staat in de bijsluiter, zie **bijlage C**.

7. Wat zijn de voordelen en de nadelen als u meedoet aan het onderzoek?

Meedoen aan het onderzoek kan voordelen en nadelen hebben. Hieronder zetten we ze op een rij. Denk hier goed over na, en praat erover met anderen.

Voordelen:
Groep 1: Het gepersonaliseerd behandelplan heeft gezorgd voor een gerichte toediening van infuusvloeistof en eventueel bloeddruk verhogende medicatie voor sepsis. Het heeft mogelijk de kans verlaagd op aanhoudende lage bloeddruk of te veel vocht, maar zeker is dat niet.
Op elk moment tijdens dit onderzoek kan de (mogelijke) sepsis verslechteren. U wordt optimaal behandeld volgens het gepersonaliseerde behandelplan.

Copy

Groep 2: Tijdens het standaard behandelplan hoeft u geen extra Starling metingen te ondergaan. Uw behandeld arts zal op basis van de huidige richtlijnen infuusvloeistof geven en bloeddruk verhogende medicatie geven wanneer nodig. Op elk moment tijdens dit onderzoek kan de (mogelijke) sepsis verslechteren. U wordt optimaal behandeld volgens het standaard behandelplan.

Nadelen:
U heeft last kunnen krijgen van de metingen tijdens het onderzoek of nadelige effecten van het gepersonaliseerde behandelplan, zoals beschreven in paragraaf 6.

Wilt u niet meedoen?
U beslist zelf of u meedoet aan het onderzoek. Wilt u niet meedoen? Dan krijgt u de standaardbehandeling voor uw (mogelijke) sepsis. Uw arts kan u meer vertellen over de behandelingsmogelijkheden die er zijn. En over de voor- en nadelen daarvan.

8. Wanneer stopt het onderzoek?

De onderzoeker laat het u weten als er nieuwe informatie over het onderzoek komt die belangrijk voor u is. De onderzoeker vraagt u daarna of u blijft meedoen.

In deze situaties stopt voor u het onderzoek:

- Alle onderzoeken volgens het schema zijn voorbij.
- Het einde van het onderzoek is bereikt.
- U wilt zelf stoppen met het onderzoek. Dat mag op ieder moment. Meld dit dan meteen bij de onderzoeker. U hoeft er niet bij te vertellen waarom u stopt. U krijgt dan weer de gewone behandeling voor uw (mogelijke) sepsis.
- De onderzoeker vindt het beter voor u om te stoppen.
- Een van de volgende instanties besluit dat het onderzoek moet stoppen:
    - het Universitair Medisch Centrum Groningen
    - de overheid, of
    - de medisch-ethische commissie die het onderzoek beoordeelt.

Wat gebeurt er als u stopt met het onderzoek?
De onderzoekers gebruiken de gegevens die tot het moment van stoppen zijn verzameld.

Het hele onderzoek is afgelopen als alle deelnemers klaar zijn.

9. Wat gebeurt er na het onderzoek?

Krijgt u de resultaten van het onderzoek?
Ongeveer twee jaar na uw deelname laat de onderzoeker u weten wat de belangrijkste uitkomsten zijn van het onderzoek.

Copy

10. Wat doen we met uw gegevens?

Doet u mee met het onderzoek? Dan geeft u ook toestemming om uw gegevens te verzamelen, gebruiken en bewaren.

Welke gegevens bewaren we?
We bewaren deze gegevens
- uw naam
- uw geslacht
- gegevens over uw gezondheid
- (medische) gegevens die we tijdens het onderzoek verzamelen

Waarom verzamelen, gebruiken en bewaren we uw gegevens?
We verzamelen, gebruiken en bewaren uw gegevens om de vragen van dit onderzoek te kunnen beantwoorden. En om de resultaten te kunnen publiceren.

Hoe beschermen we uw privacy?
Om uw privacy te beschermen geven wij uw gegevens een code. Op al uw gegevens zetten we alleen deze code. De sleutel van de code bewaren we op een beveiligde plek in het Universitair Medisch Centrum Groningen (UMCG). Als we uw gegevens en lichaamsmateriaal

verwerken, gebruiken we steeds alleen die code. Ook in rapporten en publicaties over het onderzoek kan niemand terughalen dat het over u ging.

Wie kunnen uw gegevens zien?
Sommige personen kunnen wel uw naam en andere persoonlijke gegevens zonder code inzien. Dit zijn mensen die controleren of de onderzoekers het onderzoek goed en betrouwbaar uitvoeren. Deze personen kunnen bij uw gegevens komen:

- Leden van de commissie die de veiligheid van het onderzoek in de gaten houdt.
- Een controleur die voor het Universitair Medisch Centrum werkt.
- Nationale en internationale toezichthoudende autoriteiten. Bijvoorbeeld de Inspectie Gezondheidszorg en Jeugd.

Deze personen houden uw gegevens geheim. Wij vragen u voor deze inzage toestemming te geven.

Hoelang bewaren we uw gegevens?
We bewaren uw gegevens 15 jaar in het Universitair Medisch Centrum Groningen (UMCG).

Mogen we uw gegevens gebruiken voor ander onderzoek?
Uw gegevens kunnen na afloop van dit onderzoek ook nog van belang zijn voor ander wetenschappelijk onderzoek op het gebied van sepsis en/of van de verdere ontwikkeling van het gepersonaliseerde behandelplan met de Starling monitor. Daarvoor zullen uw gegevens 15 jaar worden bewaard in het UMCG. In het toestemmingformulier geeft u aan of u dit goed vindt. Geeft u geen toestemming? Dan kunt u nog steeds meedoen met dit onderzoek. U krijgt dezelfde zorg.

Copy

Wat gebeurt er bij onverwachte ontdekkingen?
Tijdens het onderzoek kunnen we toevallig iets vinden dat belangrijk is voor uw gezondheid. De onderzoeker neemt dan contact op met uw behandeld arts. U bespreekt dan met uw huisarts of specialist wat er moet gebeuren. U geeft met het formulier toestemming voor het informeren van uw huisarts of specialist.

Kunt u uw toestemming voor het gebruik van uw gegevens weer intrekken?
U kunt uw toestemming voor het gebruik van uw gegevens op ieder moment intrekken. Maar let op: trekt u uw toestemming in, en hebben onderzoekers dan al gegevens verzameld voor een onderzoek? Dan mogen zij deze gegevens nog wel gebruiken.

Wilt u meer weten over uw privacy?

- Wilt u meer weten over uw rechten bij de verwerking van persoonsgegevens? Kijk dan op www.autoriteitpersoonsgegevens.nl.
- Heeft u vragen over uw rechten? Of heeft u een klacht over de verwerking van uw persoonsgegevens? Neem dan contact op met degene die verantwoordelijk is voor de verwerking van uw persoonsgegevens. Voor uw onderzoek is dat:

  - Het Universitair Medisch Centrum Groningen. Zie **bijlage A** voor contactgegevens, en website.
  - Meer informatie over uw privacy rechten kunt u vinden in het UMCG Privacy statement uwprivacy.umcg.nl.
- Als u klachten heeft over de verwerking van uw persoonsgegevens, raden we u aan om deze eerst te bespreken met het onderzoeksteam. Bij vragen of klachten over de verwerking van uw persoonsgegevens raden we u aan contact op te nemen met de Functionaris voor de Gegevensbescherming van het UMCG (050-361 61 61; privacy@umcg.nl). Of u dient een klacht in bij de Autoriteit Persoonsgegevens.

Waar vindt u meer informatie over het onderzoek?
Op de volgende website(s) vindt u meer informatie over het onderzoek: www.ClinicalTrials.gov. Na het onderzoek kan de website een samenvatting van de resultaten van dit onderzoek tonen. U vindt het onderzoek ook door te zoeken op www.acutelines.nl.

11. Krijgt u een vergoeding als u meedoet aan het onderzoek?

De extra onderzoeken voor het onderzoek kosten u niets. U krijgt ook geen vergoeding als u meedoet aan dit onderzoek.

12. Bent u verzekerd tijdens het onderzoek?

Copy

Voor iedereen die meedoet aan dit onderzoek is een verzekering afgesloten. De verzekering betaalt voor schade door het onderzoek. Maar niet voor alle schade. In **bijlage B** vindt u meer informatie over de verzekering en de uitzonderingen. Daar staat ook aan wie u schade kunt melden.

13. We informeren uw behandelend arts

De onderzoeker informeert uw behandelend arts mondeling en middels een notitie in uw dossier om te laten weten dat u meedoet aan het onderzoek. Dit is voor uw eigen veiligheid.

14. Heeft u vragen?

Vragen over het onderzoek kunt u stellen aan het FLUIDS-team of de projectleider van dit onderzoek. Wilt u advies van iemand die er geen belang bij heeft? Ga dan naar dr. B.D. Westenbrink. Hij weet veel over het onderzoek, maar werkt niet mee aan dit onderzoek.
Heeft u een klacht? Bespreek dit dan met de onderzoeker of de arts die u behandelt. Wilt u dit liever niet? Ga dan naar Functionaris Gegevensbescherming van het Universitair Medisch Centrum Groningen. In **bijlage A** staat waar u die kunt vinden.

15. Hoe geeft u toestemming voor het onderzoek?

Omdat u zich in een ernstige situatie bevond op de Spoedeisende Hulp, hebben we direct moeten starten met de noodzakelijke behandeling. Daarbij is besloten direct te starten met de behandeling van uw lage bloeddruk door middel van de onderzoeksmethode. Indien de situatie dat toeliet, hebben we u (of uw naaste) op de Spoedeisende Hulp mondeling geïnformeerd

over het onderzoek. Er zijn gegevens verzameld ten behoeve van het onderzoek die niet op een later moment verzameld kunnen worden. Om u voldoende bedenktijd te geven, kunt u op een later moment toestemming geven, zodat we de verzamelde gegevens mogen gebruiken voor het onderzoek. U heeft tot 30 dagen na uw deelname aan de studie de tijd om uw toestemming te geven. Dit geeft u de gelegenheid om rustig na te denken of we de verzamelde gegevens mogen gebruiken voor het onderzoek.

- Wilt u meedoen? Dan vult u het toestemmingsformulier in dat u bij deze informatiebrief vindt (**bijlage D of E**). U en de onderzoeker krijgen allebei een getekende versie van deze toestemmingsverklaring.
- Wilt u niet meedoen? Of hebben wij geen contact met u kunnen leggen na 30 dagen? Dan wordt aangenomen dat u geen toestemming geeft. De tot dan toe verzamelde gegevens zullen vernietigd worden.
- Heeft u toestemming gegeven, maar wilt u toch niet meer meedoen? U kunt uw toestemming intrekken, echter blijven de tot dan toe verzamelde gegevens bewaard.

Dank voor uw tijd.

Copy

16. Bijlagen bij deze informatie

A. Contactgegevens Universitair Medisch Centrum Groningen

B. Informatie over de verzekering

C. Omschrijving stappenplan van de FLUIDS-studie

D. Toestemmingsformulier proefpersoon

E. Toestemmingsformulier wettelijk vertegenwoordiger

Copy

Bijlage A: contactgegevens voor het Universitair Medisch Centrum Groningen

Het **FLUIDS-team** is bereikbaar voor dagelijkse vragen en opmerkingen over de FLUIDS-studie.
Contactgegevens **FLUIDS-team**:
E-mail: fluids@seh.umcg.nl
Telefoonnummer: Telefonisch via +31 (0)50-3616161, vraag naar FLUIDS (piepernummer *69443*)
Website: acutelines.umcg.nl
Postadres: Acutelines/FLUIDS UMCG, Huispostcode TA10, Antwoordnummer 299, 9700 RB Groningen
Bereikbaar alle dagen van de week, van 9 uur tot 21 uur.

De **coördinerende onderzoeker** is bereikbaar voor de algemene zaken van de FLUIDS-studie.
Naam: S. Ter Horst, arts-onderzoeker
E-mail: s.ter.horst@umcg.nl
Telefoonnummer: +31 6 16684988
Bereikbaar tijdens werkdagen van 9 uur tot 17 uur.

Copy

De **hoofdonderzoeker** van het Universitair Medisch Centrum Groningen is betrokken bij de algemene zaken van de FLUIDS-studie.
Naam: H.R. Bouma, stafarts Interne Acute Geneeskunde, MD PhD
E-mail: h.r.bouma@umcg.nl
Telefoonnummer: +31 (0)50 3616161 (piepernummer 69649)

Onafhankelijk arts: B.D. Westenbrink, stafarts Cardiologie, MD PhD
E-mail: b.d.westenbrink@umcg.nl
Telefoonnummer: +31 (0)50 3616161 (piepernummer 12207)

Klachten:
Mocht u klachten hebben over de FLUIDS-studie, dan kunt u zich wenden tot uw behandelend arts of bij ondergetekenden. Als u dit niet wilt, kunt u ook contact opnemen met de afdeling ‘Patiënteninformatie en Klachtopvang’ van het UMCG (050-3613300). Bij vragen of klachten over de verwerking van uw persoonsgegevens raden we u aan om contact op te nemen met de Functionaris voor de Gegevensbescherming van het UMCG (050-3616161), email: privacy@umcg.nl).

Copy

Bijlage B: informatie over de verzekering

Het Universitair Medisch Centrum Groningen heeft een verzekering afgesloten voor iedereen die meedoet aan het onderzoek. De verzekering betaalt de schade die u heeft doordat u aan het onderzoek meedeed. Het gaat om schade die u krijgt tijdens het onderzoek, of binnen 4 jaar na het onderzoek. U moet schade binnen 4 jaar melden bij de verzekeraar.

Heeft u schade door het onderzoek? Meld dit dan bij deze verzekeraar:

De verzekeraar van het onderzoek is:	
Naam:	Centramed
Adres:	Maria Montessorilaan 9, 2719 DB Zoetermeer
Telefoonnummer:	070 301 70 70
E-mail:	info@centramed.nl

De verzekering betaalt maximaal € *650.000* per persoon en € *5.000.000* zijn voor het hele onderzoek en € *7.500.000* per jaar voor alle onderzoeken van dezelfde opdrachtgever.

Let op: de verzekering dekt de volgende schade **niet**:

- Schade door een risico waarover we u informatie hebben gegeven in deze brief. Maar dit geldt niet als het risico groter bleek te zijn dan we van tevoren dachten. Of als het risico heel onwaarschijnlijk was.
- Schade aan uw gezondheid die ook zou zijn ontstaan als u niet aan het onderzoek had meegedaan.
- Schade die ontstaat doordat u aanwijzingen of instructies niet of niet goed opvolgde.
- Schade aan de gezondheid van uw kinderen of kleinkinderen.
- Schade door een behandelmethode die al bestaat. Of door onderzoek naar een behandelmethode die al bestaat.

Deze bepalingen staan in het 'Besluit verplichte verzekering bij medisch-wetenschappelijk onderzoek met mensen 2015'. Dit besluit staat in de Wettenbank van de overheid (https://wetten.overheid.nl).

Copy

Bijlage C: Omschrijving stappenplan van de FLUIDS-studie

Stappenplan FLUIDS

Uitleg over het gepersonaliseerde behandelplan voor de patiënt

STAP 1 We gaan uw hart en bloedvaten meten met een nieuwe monitor. Dit gebeurt meerdere keren binnen drie uur terwijl u op de Spoedeisende Hulp (SEH) bent.

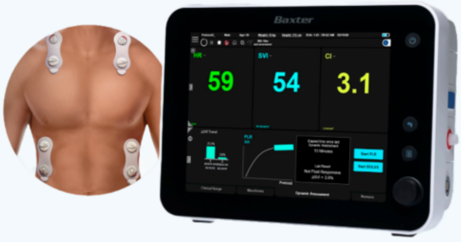

Baxter Starling monitor

Wanneer en wat meten we?

- Telkens wanneer u infuusvloeistof krijgt, meten we met de monitor
- Wij meten het effect van de infuusvloeistof op uw hart en vaten

STAP 2 Uw behandeling wordt bepaald door de metingen van uw hart en vaten. Op basis van deze metingen krijgt u infuusvloeistof of bloeddruk verhogende medicatie.

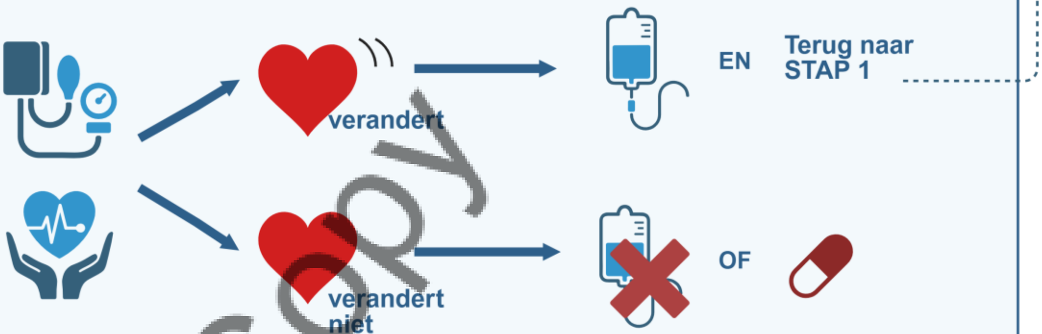

Copy

STAP 3 U krijgt voor drie uur de gepersonaliseerde of standaard behandeling. Vervolgens komt de onderzoeker langs voor extra onderzoeken om het effect te meten. Dit gebeurd tot twee dagen na uw SEH bezoek

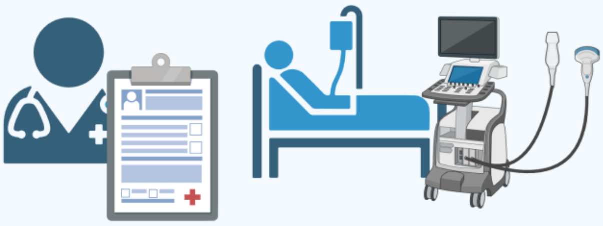

Wat zijn de extra onderzoeken?

- 2 keer echo-metingen voor metingen van vocht in het lichaam
- 4 keer lichamelijk onderzoek (bijvoorbeeld het luisteren naar hart en longen)

Copy

Bijlage D: toestemmingsformulier proefpersoon

Behorende bij FLUIDS

Vocht management en geïndividualiseerde resuscitatie in sepsis

- Ik heb de informatiebrief gelezen. Ook kon ik vragen stellen. Mijn vragen zijn goed genoeg beantwoord. Ik had genoeg tijd om te beslissen of ik meedoe.
- Ik weet dat meedoen vrijwillig is. Ook weet ik dat ik op ieder moment kan beslissen om toch niet mee te doen met het onderzoek. Of om ermee te stoppen. Ik hoef dan niet te zeggen waarom ik wil stoppen.
- Ik geef de onderzoeker toestemming om mijn huisarts en behandelend arts te laten weten dat ik meedoe aan dit onderzoek.
- Ik geef de onderzoeker toestemming om huidige en toekomstige informatie op te vragen uit mijn medisch dossier over mijn algemene en medische gegevens.
- Ik geef de onderzoeker toestemming om mijn huisarts of specialist informatie te geven over onverwachte bevindingen uit het onderzoek die van belang zijn voor mijn gezondheid.
- Ik geef de onderzoekers toestemming om mijn gegevens te verzamelen en gebruiken. De onderzoekers doen dit alleen om de onderzoeksvraag van dit onderzoek te beantwoorden.
- Ik weet dat voor de controle van het onderzoek sommige mensen al mijn gegevens kunnen inzien. Die mensen staan in deze informatiebrief. Ik geef deze mensen toestemming om mijn gegevens in te zien voor deze controle.

Copy

- Wilt u in de tabel hieronder ja of nee aankruisen?

Ik geef toestemming om mijn gegevens te bewaren om dit te gebruiken voor ander onderzoek, zoals in de informatiebrief staat.	Ja ☐	Nee☐

- Ik wil meedoen aan dit onderzoek.

**FLUIDS-ID:**	

Mijn naam is (proefpersoon): ………………………………..
Handtekening: ……………………… Datum : __ / __ / __

Ik verklaar dat ik deze proefpersoon volledig heb geïnformeerd over het genoemde onderzoek.

Wordt er tijdens het onderzoek informatie bekend die die de toestemming van de proefpersoon kan beïnvloeden? Dan laat ik dit op tijd weten aan deze proefpersoon.

Naam onderzoeker (of diens vertegenwoordiger):……………………………….
Handtekening:……………………… Datum: __ / __ / __

De proefpersoon krijgt een volledige informatiebrief mee, samen met een getekende versie van het toestemmingsformulier.

Copy

Bijlage E: Toestemmingsformulier vertegenwoordiger

Behorende bij FLUIDS
Vocht management en geïndividualiseerde resuscitatie in sepsis

Ik ben gevraagd om toestemming te geven voor deelname deze persoon aan dit medisch-wetenschappelijke onderzoek:

Naam proefpersoon: …………………….. Geboortedatum: __ / __ / __

- Ik heb de informatiebrief voor de proefpersoon/vertegenwoordiger gelezen. Ook kon ik vragen stellen. Mijn vragen zijn goed genoeg beantwoord. Ik had genoeg tijd om te beslissen of ik wil dat deze persoon meedoet.
- Ik weet dat meedoen vrijwillig is. Ook weet ik dat ik op ieder moment kan beslissen dat deze persoon toch niet mee doet. Ik hoef dan niet te zeggen waarom ik dat wil.
- Ik geef de onderzoeker toestemming om de huisarts en behandelend arts te laten weten dat deze persoon meedoet aan dit onderzoek.
- Ik geef de onderzoeker toestemming om huidige en toekomstige informatie op te vragen uit het medisch dossier over de algemene en medische gegevens
- Ik geef de onderzoeker toestemming om de huisarts en/of specialist van deze persoon informatie te geven over onverwachte uitkomsten van het onderzoek die van belang zijn voor de gezondheid van deze persoon.
- Ik geef de onderzoekers toestemming om de gegevens van deze persoon te verzamelen en te gebruiken. De onderzoekers doen dit om alleen de onderzoeksvraag in dit onderzoek te beantwoorden.
- Ik weet dat voor de controle van het onderzoek sommige mensen toegang tot alle gegevens van deze persoon kunnen krijgen. Die mensen staan in deze informatiebrief. Ik geef deze mensen toestemming om de gegevens van deze persoon in te zien voor deze controle.
- Wilt u in de tabel hieronder ja of nee aankruisen?

Ik geef toestemming om mijn gegevens te bewaren om dit te gebruiken voor ander onderzoek, zoals in de informatiebrief staat.	Ja ☐	Nee☐

- Ik ga ermee akkoord dat deze persoon meedoet aan dit onderzoek.

Copy

**FLUIDS-ID:**	

Naam wettelijk vertegenwoordiger:……………………………
Relatie tot de proefpersoon: ……………………………………
Handtekening: ………………………………… Datum: __ / __ / __

Ik verklaar dat ik de persoon/personen hierboven volledig heb geïnformeerd over het genoemde onderzoek.

Wordt er tijden het onderzoek informatie bekend die de toestemming van de vertegenwoordiger kan beïnvloeden? Dan laat ik dit op tijd aan hem/haar weten.

Naam onderzoeker (of diens vertegenwoordiger):………………………
Handtekening: …………………… Datum: __ / __ / __

De vertegenwoordiger krijgt een volledige informatiebrief mee, samen met een getekende versie van het toestemmingsformulier.

Copy
