# Supplementary material for: FLUid management and InDividualized resuscitation in Sepsis (FLUIDS)—A Study protocol for a single-centre, open-label, randomized clinical trial
Source: PLoS One. 2025 Dec 19;20(12):e0338504. doi: 10.1371/journal.pone.0338504 (PMC12716701; doi:10.1371/journal.pone.0338504)
Supplement: S3 File — Supplementary material containing the original study protocol, provided as copy in English. (PDF) [file pone.0338504.s003.pdf]

FLUIDS

NL84833.099.24

Copy

'FLUIDS – Fluid management and Individualized resuscitation in Sepsis'

**Protocol ID**	**FLUIDS**
**Short title**	FLUIDS
**EudraCT number**	Not applicable
**Version**	7
**Date**	October 8, 2025
**Coordinating investigator/project leader**	**S. Ter Horst, MD**<br>MD/PhD candidate Acute Medicine<br>Internal Medicine, University Medical Center Groningen<br>Cell Phone: +31 6 16684988<br>E-mail: s.ter.horst@umcg.nl
**Principal investigator(s) (in Dutch: hoofdonderzoeker/ uitvoerder)**	**H.R. Bouma, MD, PhD, EuCP (PI)**<br>Internist-Acute Medicine/Pharmacologist<br>h.r.bouma@umcg.nl
**Co-investigators**	**J.C. ter Maaten, MD, PhD**<br>Internist-Acute Medicine<br>**T.J. Olgers, MD**<br>Internist-Acute Medicine<br>**E. Ter Avest, MD, PhD**<br>Emergency Medicine Physician<br>**M. van Meurs, MD, PhD**<br>Intensivist<br>**J.J. Vos, MD, PhD**<br>Anesthesiologist<br>**K. Damman, MD, PhD**<br>Cardiologist<br>**J. van Everdink, MD**<br>Fellow Acute Medicine
**Sponsor**	University Medical Center Groningen
**Subsidising party**	University Medical Center Groningen
**Independent expert (s)**	**B.D. Westenbrink, MD, PhD**<br>Cardiologist
**Laboratory sites**	Not applicable
**Pharmacy**	Not applicable

Copy

PROTOCOL SIGNATURE SHEET

Name	Signature	Date
**Sponsor or legal representative:**<br>**Head of Department:**<br>Prof.dr. J.C. ter Maaten		**October 8, 2025**
**Project leader/Principal Investigator:**<br>Prof.dr. H.R. Bouma		**October 8, 2025**

Copy

TABLE OF CONTENTS

1. INTRODUCTION AND RATIONALE ... 9
2. OBJECTIVES ... 11
3. STUDY DESIGN ... 12
4. STUDY POPULATION ... 14
4.1 Population (base) ... 14
4.2 Inclusion criteria ... 14
4.3 Exclusion criteria ... 14
4.4 Sample size calculation ... 15
5. TREATMENT OF SUBJECTS ... 16
5.1 Investigational product/treatment ... 16
5.2 Use of co-intervention (if applicable) ... 17
5.3 Escape medication (if applicable) ... 17
6. INVESTIGATIONAL PRODUCT ... 18
6.1 Name and description of investigational product(s) ... 18
6.2 Summary of findings from non-clinical studies ... 19
6.3 Summary of findings from clinical studies ... 19
6.4 Summary of known and potential risks and benefits ... 19
6.5 Description and justification of route of administration and dosage ... 20
6.6 Dosages, dosage modifications and method of administration ... 20
6.7 Preparation and labelling of Non-Investigational Medicinal Product ... 20
6.8 Drug accountability ... 20
7. NON-INVESTIGATIONAL PRODUCT ... 21
8. METHODS ... 22
8.1 Study parameters/endpoints ... 22
8.1.1 Main study parameter/endpoint ... 22
8.1.2 Secondary study parameters/endpoints ... 22
8.1.3 Other study parameters (if applicable) ... 23
8.2 Randomisation, blinding and treatment allocation ... 24
8.3 Study procedures ... 24
8.4 Withdrawal of individual subjects ... 27
8.4.1 Specific criteria for withdrawal (if applicable) ... 27
8.5 Replacement of individual subjects after withdrawal ... 27
8.6 Follow-up of subjects withdrawn from treatment ... 27
8.7 Premature termination of the study ... 27
9. SAFETY REPORTING ... 28
9.1 Temporary halt for reasons of subject safety ... 28
9.2 AEs, SAEs and SUSARs ... 28
9.2.1 Adverse events (AEs) ... 28
9.2.2 Serious adverse events (SAEs) ... 28
9.2.3 Suspected unexpected serious adverse reactions (SUSARs) ... 28
9.3 Annual safety report ... 28

Copy

9.4 Follow-up of adverse events 29
9.5 [Data Safety Monitoring Board (DSMB) / Safety Committee] 29
10. STATISTICAL ANALYSIS 30
10.1 Primary study parameter(s) 30
10.2 Secondary study parameter(s) 30
10.3 Supplementary subgroup analysis 30
10.4 Missing data 30
10.5 Other study parameters 31
10.6 Interim analysis 31
11. ETHICAL CONSIDERATIONS 32
11.1 Regulation statement 32
11.2 Recruitment and consent 32
11.3 Objection by minors or incapacitated subjects 33
11.4 Benefits and risks assessment, group relatedness 33
11.5 Compensation for injury 34
11.6 Incentives 34
12. ADMINISTRATIVE ASPECTS, MONITORING AND PUBLICATION 35
12.1 Handling and storage of data and documents 35
12.2 Monitoring and Quality Assurance 35
12.3 Amendments 35
12.4 Annual progress report 35
12.5 Temporary halt and (prematurely) end of study report 35
12.6 Public disclosure and publication policy 36
13. STRUCTURED RISK ANALYSIS 37
13.1 Potential issues of concern 37
13.2 Synthesis 38
14. REFERENCES 39

Copy

LIST OF ABBREVIATIONS AND RELEVANT DEFINITIONS

ABR	General Assessment and Registration form (ABR form), the application form that is required for submission to the accredited Ethics Committee; in Dutch: Algemeen Beoordelings- en Registratieformulier (ABR-formulier)
AE	Adverse Event
AR	Adverse Reaction
AKI	Acute Kidney Injury
CA	Competent Authority
CCMO	Central Committee on Research Involving Human Subjects; in Dutch: Centrale Commissie Mensgebonden Onderzoek
CO	Cardiac Output
CRT	Capillary Refill Time
CV	Curriculum Vitae
DC	Deferred Consent
DSMB	Data Safety Monitoring Board
ECG	Electrocardiography
ECMO	Extra Corporale Membraan Oxygenatie
ED	Emergency Department
EU	European Union
EudraCT	European drug regulatory affairs Clinical Trials
GCP	Good Clinical Practice
GDPR	General Data Protection Regulation; in Dutch: Algemene Verordening Gegevensbescherming (AVG)
HDS	Hemodynamic Stability
HF	Heart Failure
HRV	Heart Rate Variability
IB	Investigator’s Brochure
IC	Informed Consent
ICU	Intensive Care Unit
IFU	Instructions for Use
IMP	Investigational Medicinal Product
IMPD	Investigational Medicinal Product Dossier
IV	Intravenous
IVC	Inferior Vena Cava
KDIGO	Kidney Disease Improving Global Outcomes

Copy

LVAD	Left Ventricular Assist Device
MAP	Mean Arterial Pressure
METC	Medical research ethics committee (MREC); in Dutch: medisch-ethische toetsingscommissie (METC)
NEWS	National Early Warning Score
NYHA	New York Heart Association
PoCUS	Point-of-Care Ultrasound
PPG	Photoplethysmography
RRT	Renal Replacement Therapy
(S)AE	(Serious) Adverse Event
SBP	Systolic Blood Pressure
SIRS	Systemic Inflammatory Response Syndrome
qSOFA	(quick) Sequential Organ Failure Assessment
SPC	Summary of Product Characteristics; in Dutch: officiële productinformatie IB1-tekst
SI	Shock Index
Sponsor	The sponsor is the party that commissions the organisation or performance of the research, for example a pharmaceutical company, academic hospital, scientific organisation or investigator. A party that provides funding for a study but does not commission it is not regarded as the sponsor, but referred to as a subsidising party.
SUSAR	Suspected Unexpected Serious Adverse Reaction
TPR	Total Peripheral Resistance
UAVG	Dutch Act on Implementation of the General Data Protection Regulation; in Dutch: Uitvoeringswet AVG
UMCG	University Medical Centre Groningen
WMO	Medical Research Involving Human Subjects Act; in Dutch: Wet Medisch-wetenschappelijk Onderzoek met Mensen

Copy

SUMMARY

Rationale: Sepsis is a life-threatening condition characterized by organ dysfunction resulting from an uncontrolled host response to infection. The pathophysiological changes in sepsis affect both micro- and macrovascular components, necessitating early hemodynamic resuscitation to prevent organ failure and death. However, the current one-size-fits-all approach may lead to prolonged shock or fluid overload with its complications. The Baxter Starling SV monitor, which provides real-time cardiac output and total peripheral resistance measurements, has the potential to guide personalized resuscitation for sepsis patients. The objective of this study is to assess the Starling SV monitor to guide hemodynamic resuscitation for sepsis patients within the first three hours of their emergency department (ED) admission, in comparison to the existing treatment protocol.

Objective: The primary objective of the current project is to assess the Starling SV monitor to guide hemodynamic resuscitation for sepsis patients within the first three hours of their emergency department (ED) admission, in comparison to the existing treatment protocol. By focusing on patients admitted to the emergency department, the study has the potential to optimize the management of sepsis, improve outcomes, and enhance patient care in this life-threatening condition.

Study design: A randomized open clinical trial conducted in the Emergency Department (ED) of the University Medical Centre Groningen (UMCG). Adult patients with suspected infection at the ED will be recruited and randomly assigned to either personalized hemodynamic resuscitation using the Starling SV monitor (intervention group) or standard care (control group). We will include in total 188 patients in the study, during an estimated duration of 12 months.

Study population: The study population includes adult patients referred to the emergency department (ED) presenting with confirmed or suspected infection. Patients requiring hemodynamic resuscitation and meeting specific inclusion criteria will be eligible for participation.

Intervention: In the experimental arm, personalized hemodynamic resuscitation will be guided by the Starling SV monitor, with fluid responsiveness assessed during fluid boluses administered based on the increase in stroke volume index as measured by the Starling SV monitor. The control arm will receive standard care determined by the treating physician. All fluids administered will be balanced fluids.

Main study parameters/endpoints: The primary end point is the volume (in liters) of IV fluid resuscitation within three hours since ED admission.

Nature and extent of the burden and risks associated with participation, benefit and group relatedness: In this intervention study, the utilization of the Starling SV monitor provides significant benefits for sepsis patients in the ED. By tailoring treatment plans to individual hemodynamic changes, patients receive precise fluid administration and timely initiation of vasopressor therapy, reducing the risks of prolonged hypotension and fluid overload. This personalized approach has the potential to optimize sepsis management and improve outcomes. Although there are minimal risks associated with the monitor, such as minimal adverse events and the potential for device malfunction or misinterpretation of readings, proper training, adherence to protocols, and regular evaluation can mitigate these risks and ensure patient safety. Overall, the benefits of the Starling SV monitor outweigh its risks, making it a valuable tool in improving patient outcomes.

Copy

1. INTRODUCTION AND RATIONALE

Sepsis is the leading cause of death worldwide from infection (1) with an in-hospital mortality rate of 10%. (1,2) In 2017, sepsis-related deaths were estimated to account for 19.7% (*one out of five*) of all global deaths. (3) Sepsis is a life-threatening condition characterized by organ dysfunction that arises from an uncontrolled host response to an infection. (1,2,4) In the early stages of sepsis, both the micro- and macrovascular components are affected by pathophysiological changes. (4,5) Endothelial dysfunction due to mitochondrial dysfunction and inflammation is observed on a microvascular level, while vasodilation, reduced total peripheral resistance, and cardiomyopathy contribute to macrovascular failure, which can lead to shock in sepsis. (4,5) Septic shock has an in-hospital mortality rate of at least 40%. (2,5) **Early treatment of hemodynamic instability in a septic patient is of utmost importance to prevent organ failure and death.**

Current treatment for sepsis consists of hemodynamic resuscitation, which is a combination of fluid resuscitation and vasopressor therapy, and antibiotic treatment. (1) **However, a one-size-fits-all approach, the 30 ml/kg/3h fluid resuscitation protocol, is problematic due to significant inter-individual differences in sepsis. This approach can lead to prolonged shock or fluid overload, both of which can be harmful.** In sepsis patients, cardiovascular changes create a delicate balance between fluid responsiveness and fluid tolerance. (6,7) Whilst in some patients fluid administration results in an increase in cardiac output (*fluid responsive*), a similar amount of fluids administered to others will not and may even result in fluid overloading (*fluid intolerant*). (6–9) A more personalized hemodynamic therapy approach may lead to improved patient outcomes. (10,11) Understanding the pathophysiology of hemodynamic deterioration in sepsis can provide insights into the development of personalized resuscitation plans for hemodynamically unstable septic patients.

The **Starling SV monitor** is a state-of-the-art technology that provides continuous, real-time information on cardiac index, cardiac output, stroke volume index, and total peripheral resistance. (12)This system is capable of detecting cardiovascular changes in sepsis and guiding hemodynamic resuscitation for individual septic patients. (13) Evidence to date, however, is limited to severely ill patients where intensive care unit (ICU) admission was already foreseen (6), where already a relevant amount of fluids was administered. (14) Although feasibility at the emergency department (ED) has been demonstrated, (15) the added value for clinical decision making has not yet been established. (16)

Based on the pathophysiological cardiovascular changes in early sepsis, **we hypothesize that point-of-care measurements of stroke volume (SV), cardiac output (CO) and total peripheral resistance (TPR) by the Starling SV monitor can be used to expand our understanding of interindividual differences in the clinical course of sepsis and develop personalized resuscitation plans for patients with sepsis at the ED.** Therefore, the primary objective of the current project is to assess the effect of the personalized hemodynamic resuscitation protocol guided by the Starling SV monitor on the volume (in liters) of intravenous (IV) fluid resuscitation within the first three hours of their emergency department (ED) admission.

Since most sepsis patients are admitted to the general ward, rather than ICU, we will focus on patients with sepsis that are admitted via the emergency department. With its potential to improve outcomes for sepsis patients, this innovative technology has the potential to optimize the management of this life-threatening condition. We decided to focus on the first three hours of hemodynamic resuscitation, which is the period when - currently - patients receive one-size-fits-all fluid resuscitation per protocol, which may be followed by the start of vasopressors at the ICU. We expect that personalized hemodynamic resuscitation using non-invasive CO and TPR measurements during the initial resuscitation at the ED and the following three hours, will

Copy

reveal to be an efficacious strategy to reduce the time to hemodynamic stability, while limiting the risk of overhydration.

Copy

2. OBJECTIVES

Primary Objective:
The primary objective is to assess the effect of the personalized hemodynamic resuscitation protocol guided by the Starling SV monitor on the volume (in liters) of intravenous (IV) fluid resuscitation within the first three hours of their emergency department (ED) admission.

Secondary Objective(s):

Efficacy

- To assess the effect of the personalized hemodynamic resuscitation protocol guided by the Starling SV monitor on the initiation of vasopressor therapy, measured in time, within the first three hours of their ED admission.
- To evaluate the effect of the personalized hemodynamic resuscitation protocol guided by the Starling SV monitor in guiding hemodynamic resuscitation on organ dysfunction scores within 48 hours.
- To compare fluid balance (in litres) within three hours between the sepsis patient group receiving hemodynamic resuscitation guided by the Starling SV monitor and the group receiving standard care.
- To assess the impact of the use of a personalized hemodynamic resuscitation protocol on the length-of-stay in the hospital for sepsis patients admitted to the ED.
- To evaluate the effect of the use of a personalized hemodynamic resuscitation protocol on the 7-day and 30-day mortality rate of sepsis patients.
- To assess the amount of fluid administered within the first three hours of their ED admission between the fluid responder group and the fluid non-responder group.

Safety

- To examine the occurrence of adverse events related to the use of a personalized hemodynamic resuscitation protocol within 48 hours and 7 days.
- To assess the rate of acute kidney injury associated with the use of a personalized hemodynamic resuscitation protocol within 48 hours and 7 days.

Copy

Health technology:

- To compare the cost-effectiveness when using the personalized hemodynamic protocol guided by Starling SV monitor versus standard protocols at the emergency department (ED).

Exploratory mechanistic endpoints:

- To explore the inter-individual differences in hemodynamic endotypes between sepsis patients at the emergency department (ED).
- To compare the accuracy of the Starling SV monitor with other diagnostic tools such as PoCUS at triage in diagnosing fluid responsiveness in sepsis patients.
- To explore other diagnostic tools in the stratification of hemodynamic endotypes between sepsis patients.

3. STUDY DESIGN

Study design
This is a **randomized open clinical trial**. Patients will be recruited by the FLUIDS-team of researchers in the emergency department (ED) of the University Medical Centre Groningen (UMCG). The ED physician and/or nurse will screen all patients entering the ED for inclusion in the current study (on weekdays, between 9 am - 5 pm). The FLUIDS-team of researchers will subsequently enroll eligible patients in the study protocol. To ensure timely intervention in the acute setting and limit selection bias, we use deferred consent (by proxy).

Strategy/setting
Adult patients with a suspected infection at the ED (*see section 'key inclusion criteria' for definition)* and able to maintain a supine position, will be recruited. In this study, we will compare the Starling SV monitor as guide for hemodynamic resuscitation (intervention) vs standard care. Patients are assigned randomly. Randomization is in a 1:1 allocation of SVI-guided to standard care. All fluids administered will be balanced fluids.

Duration
The clinical trial is expected to span 12 months. The trial will involve a total of 188 patients. We conducted a preliminary assessment of the inclusion rate in our emergency department and identified an average of four patients per week meeting the study's criteria for inclusion.

Groups
<u>Experimental arm: personalized hemodynamic resuscitation</u> (94 patients)
Patients will receive an IV fluid bolus directly after inclusion. Next, measurement of the stroke volume index change in response to the fluid bolus by the Starling SV will be used to determine fluid responsiveness. Vital parameters and fluid responsiveness will be used to guide hemodynamic resuscitation, consisting of IV fluids and/or vasopressors, during the first 3 hours. See chapter 5 and figure 1 for a detailed description of the study procedures.

Copy

<u>Control arm: standard care</u> (94 patients)
Patients will receive an IV fluid bolus directly after inclusion. Vital parameters will be used to guide hemodynamic resuscitation, consisting of IV fluids and/or vasopressors, during the first 3 hours. See chapter 5 for a detailed description of the study procedures.

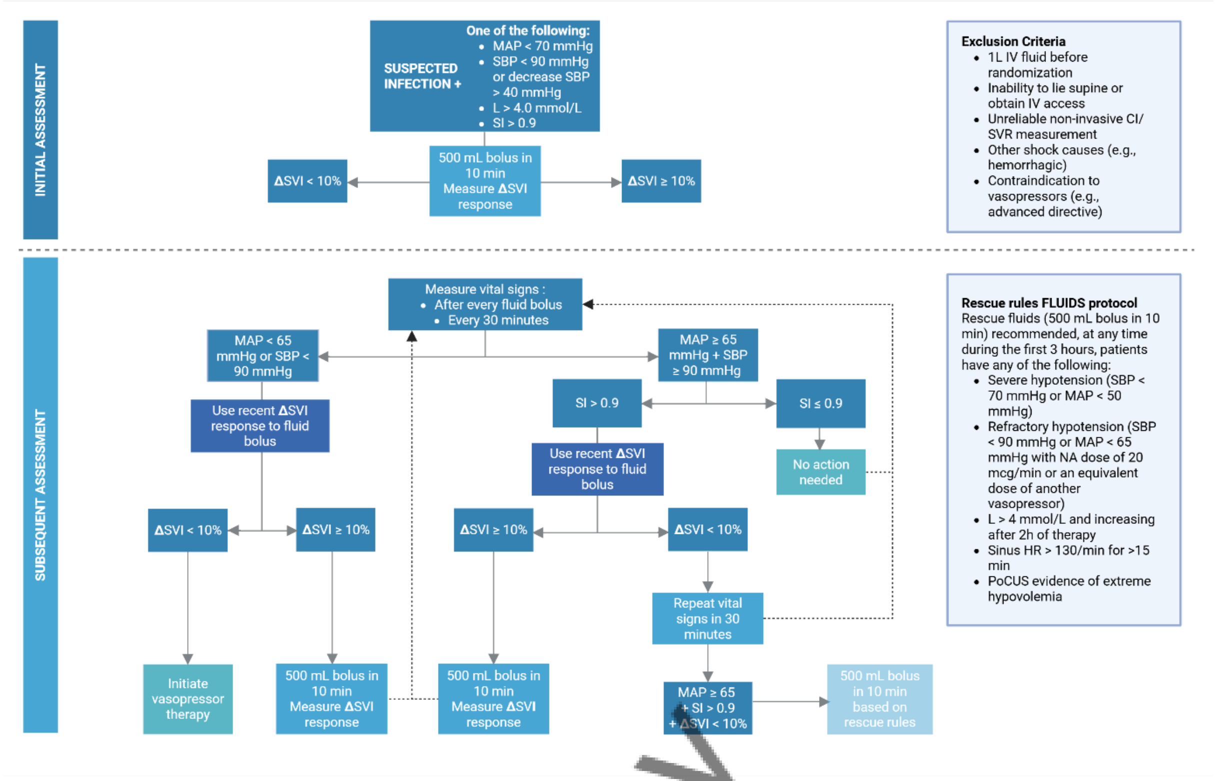

Copy

Figure 1: Flowchart personalized protocol. Adult patients with confirmed or suspected infection with need for hemodynamic resuscitation, will be included into the study. A dynamic assessment of fluid responsiveness using the Starling SV monitor will be performed at every clinical decision point with concordant treatment (volume or vasopressors). MAP: mean arterial pressure; SBP: systolic blood pressure; NE: noradrenaline; PoCUS: Point-of-Care Ultrasound; L: lactate; SI: shock index*; CI: cardiac index; PLR: passive leg raise; IV: intravenous; *shock index is heart rate divided by systolic blood pressure;

4. STUDY POPULATION

4.1 Population (base)

Subjects will be enrolled upon Emergency Department (ED) arrival. Adult patients with confirmed or suspected infection with a need for hemodynamic resuscitation will be considered a candidate for the study. A subject needs to be enrolled within one hour after ED arrival. Once enrolled, subjects will be randomly assigned using the designated randomization procedure. After confirming that the inclusion and exclusion criteria are met, the Starling sensors will be positioned and connected to the Starling SV monitor. To ensure accurate data collection during the study, the readings from the sensors will be validated before initiating the data collection period. To ensure timely intervention in this acute setting, a deferred consent (DC) strategy is employed, facilitated by a proxy if necessary. More information about the DC-procedure is available in *'Chapter 11.2: Recruitment and consent'.*

4.2 Inclusion criteria

In order to be eligible to participate in this study, a subject must meet all of the following criteria:

- Adult patients (≥ 18 years of age);
- Referred to internal medicine, nephrology, geriatric medicine, oncology, hematology, lung medicine, rheumatology, gastrointestinal / liver medicine, urology, or emergency medicine (non-trauma);
- Confirmed or suspected infection according to the physician's judgement upon arrival to the ED, based the presence of an acute phase response not due to an alternative non-infectious cause (i.e., body temperature < 36°C or >38°C, leukocyte count > 12 $x10^9$/L or C-reactive protein > 50 mg/L), and/or on symptoms suggestive for an infection (*e.g.* productive cough, dyspnea, dysuria, pollakisuria, abdominal pain, erythema)
- Need for hemodynamic resuscitation, based on any of the following (*first measurement at ED arrival [triage]*):
    - Mean arterial pressure (MAP) < 70 mmHg
    - Systolic blood pressure (SBP) < 90 mmHg or a SBP decrease >40 mmHg
    - Lactate > 4.0 mmol/L
    - Shock index* > 0.9
- Enrolled in study within one hour after ED arrival

** Shock index is the heart rate divided by the systolic blood pressure, an indicator for the severity of hypovolemic shock.*

Copy

4.3 Exclusion criteria

A potential subject who meets any of the following criteria will be excluded from participation in this study:

- Primary diagnosis of: acute cerebral vascular event, acute coronary syndrome, acute pulmonary edema, status asthmaticus, major cardiac arrhythmia, drug overdose, or injury from burn or trauma, diabetic ketoacidosis, hyper-osmolarity syndrome, pancreatitis
- Known aortic insufficiency, aortic abnormalities, or intraventricular heart defect, such as ventral septal defect or atrial septal defect
- Known advanced heart failure – meaning NYHA IV functional class HF, on waiting list for heart transplant, LVAD recipient or chronic inotrope use.
- Known end-stage kidney disease (dialysis-dependent CKD stage 5 or eGFR <15 mL/min/1.73 m²)
- Decompensated liver cirrhosis at ED admission (e.g., ascites, hepatic encephalopathy, or variceal bleeding)

- Hemodynamic instability due to active bleeding
- Patient has received >1 liter of IV fluid prior to study randomization
- Requires immediate surgery
- Transfer from another hospital after initiation of therapy (a.o. referred by another hospital ICU) or another in-hospital setting
- Pregnant women
- Trauma patients
- Suspected intra-abdominal hypertension, based on the presence of portal hypertension (i.e. presence of ascites due to liver cirrhosis, esophageal varices or as measured by Doppler ultrasound)
- Inability to obtain IV access
- Patient uncouples from treatment algorithm
- Patient should be excluded based on the opinion of the Clinician/Investigator
- Not able to commence treatment protocol within 1 hour after randomization
- Potential ICU-admission unwanted by advanced care directive (e.g., limited life expectancy)

4.4 Sample size calculation

Estimated sample size

Copy

Sample size was calculated using R (version 4.3.1), based on data from a comparable study with similar methodology and endpoint definitions (14). That reference study demonstrated a mean difference of 1.37 L in positive fluid balance at 72 hours between groups, with the intervention group showing 0.65 ± 2.85 L compared to 2.02 ± 3.44 L in the control group (14). The effect size (Cohen's d = 0.45) was calculated from the reported means and standard deviations.

We conducted an a priori power analysis via a Monte Carlo simulation (50,000 replicates) assuming normal distributions for each arm with observed means and standard deviations. To accommodate unequal variances, a two-tailed Welch's t-test ($\alpha = 0.05$) was applied in the simulated dataset. The simulation showed that 170 participants (85 per group) are needed to achieve 80% power to detect a statistically significant difference in the primary endpoint (IV fluid volume administered within the first three hours after study enrollment). Anticipating 10% drop-out due to loss to follow-up, study withdrawal, or protocol deviations, the total planned sample size is 188 participants (94 per group).

5. TREATMENT OF SUBJECTS

5.1 Investigational product/treatment

<u>Intervention:</u>

The investigational product in this study is the Starling SV monitor, which is a medical device manufactured by Baxter Healthcare. The Starling SV monitor is a non-invasive hemodynamic monitor that provides real-time continuous information about stroke volume index, cardiac index, cardiac output, and total peripheral resistance. In this intervention study, the Starling SV monitor is designed to assist in guiding hemodynamic resuscitation for patients with sepsis.

In the experimental group, patients will receive hemodynamic resuscitation guided by the Starling SV monitor. A dynamic assessment of fluid responsiveness using the monitor will be performed at every clinical decision point during the first three hours of study enrolment.

Vital parameters, including blood pressure and heart rate, are measured upon ED arrival (triage), immediately after administration of a fluid bolus and at least twice per hour during the first 3 hours. Lactate will be measured at ED arrival (triage) and will be repeated every two hours as long as lactate is > 4 mmol/L. Fluid responsiveness will be measured by the Starling SV monitor immediately before and after the administration of fluids. Fluid responsiveness is defined as an increase in stroke volume index by at least 10% after a fluid bolus of 500 mL in 10 min.

Copy

First, we will perform the first measurement during the first administration of 500 mL of IV fluids. During both the fluid bolus, we will measure the change in stroke volume index (ΔSVI) using the Starling SV and use the stroke volume index change in response to the fluid bolus to determine fluid responsiveness.

If, at any time during the first 3 hours, patients have: (see figure 1)

- MAP < 65 mmHg or systolic blood pressure < 90 mmHg:
  - Fluid responsive (on last measurement): patients will receive (another) 500 mL of IV fluids if considered fluid responsive (and a new Starling SV measurement will be obtained). Reassess after intervention.
  - Not fluid responsive (on last measurement): patients will receive vasopressor (noradrenaline), according to the standard care protocol.*
- MAP ≥ 65 mmHg or systolic blood pressure ≥ 90 mmHg combined with SI > 0.9:
  - Fluid responsive (on last measurement): patients will receive (another) 500 mL of IV fluids if considered fluid responsive (and a new Starling SV measurement will be obtained). Reassess after intervention.
  - Not fluid responsive (on last measurement): repeat measurement in 30 minutes.
    - If (MAP ≥ 65 mmHg and systolic blood pressure ≥ 90 mmHg) combined with SI > 0.9: optional 500 mL of IV fluids based on rescue rules. Reassess after intervention;
    - If MAP < 65 mmHg or systolic blood pressure < 90 mmHg: see above;
    - If (MAP ≥ 65 mmHg and systolic blood pressure ≥ 90 mmHg) combined with SI ≤ 0.9: see below.
- MAP ≥ 65 mmHg and systolic blood pressure ≥ 90 mmHg, combined with SI ≤ 0.9: no action needed.

Rescue fluids (500 mL of IV fluids) recommended if, at any time during the first 3 hours, patients have any of the following conditions:

- Severe hypotension (systolic blood pressure < 70 mmHg or MAP < 50 mmHg)

- Refractory hypotension (systolic blood pressure < 90 mmHg or MAP < 65 mmHg) with administration of noradrenaline at dose of 20 mcg/min or of an equivalent dose of another vasopressor)
- Lactate level > 4 mmol/liter and increasing after 2 h of therapy
- Sinus heart rate > 130 beats/min for > 15 min
- PoCUS evidence of extreme hypovolemia

All fluids administered will be balanced fluids. In addition to the fluid resuscitation as described above, all patients will receive a standard 1.5 L per day of IV fluids from the moment of ED arrival. The total volume of IV fluids will be limited to a maximum of 60 ml/kg over three hours to prevent hypervolemia.

* Vasopressor therapy initiated according to personalized protocol will be performed in the stepwise approach of standard care. Every 30 minutes vital parameters will be repeated. According to the protocol it can be that vasopressor therapy needs to be increased or that fluids will be administered, as vasopressors can cause fluid responsiveness due to increased venous return.

Control:
Vital parameters, including blood pressure and heart rate, are measured upon ED arrival (triage), immediately after administration of a fluid bolus and at least twice per hour during the first 3 hours. Lactate will be measured at ED arrival (triage) and will be repeated every two hours as long as lactate is > 4 mmol/L. No required therapeutic protocol will be mandated for patient treatment, which will be determined at the discretion of the physician and in accordance with hospital standards.

All fluids administered will be balanced fluids. In addition to the fluid resuscitation as described above, all patients will receive a standard 1.5 L per day of IV fluids from the moment of ED arrival. The total volume of IV fluids will be limited to a maximum of 60 ml/kg over three hours to prevent hypervolemia.

The purpose of the control group is to compare the outcomes of hemodynamic resuscitation guided by the Starling SV monitor in the experimental group with the outcomes of standard care in the control group.

5.2 Use of co-intervention (if applicable)

Not applicable

5.3 Escape medication (if applicable)

Not applicable

Copy

6. INVESTIGATIONAL PRODUCT

6.1 Name and description of investigational product(s)

The Baxter's Noninvasive Starling SV monitor with NIBP and SpO2 is a portable, noninvasive hemodynamic monitoring device that operates based on the principles of impedance. It works by measuring changes in electrical impedance within the chest cavity, which correspond to changes in the amount of blood present in the thorax during the cardiac cycle. The Starling SV monitor uses unique patented Bioreactance® technology to take measures continuously and precisely, and they require only four easy-to-place sensor pads.

The device contains four sensor pads that are placed on the patient's chest. Two of these outer pair of sensors act as current injectors, sending a low-level, high-frequency electrical signal through the chest. The other two inner pair of sensors serve as voltage detectors, measuring the resulting changes in impedance as the electrical signal encounters variations in the thoracic fluid volume during the cardiac cycle.

The flow of blood in the thorax introduces a time delay or phase shift in the signal. The monitor uses this phase shift as a baseline for stroke volume measurements. By continuously measuring these changes, the Starling SV monitor can derive various hemodynamic parameters, including stroke volume, cardiac output, and other indices of cardiac function.

The derived hemodynamic parameters help healthcare providers assess a patient's fluid status, fluid responsiveness, cardiac performance, and vascular resistance. This information can be crucial in guiding fluid resuscitation, optimizing cardiac function, and making timely clinical decisions for patients with sepsis or other conditions that affect hemodynamics.

Copy

The device displays associated hemodynamic parameters based on measurements or calculations of measurements already incorporated into the Starling SV. These parameters are:

- Cardiac Index (CI)
- Stroke Volume (SV)
- Stroke Volume Index (SVI)
- Stroke Volume Variation (SVV)
- Heart Rate (HR)
- Ventricular Ejection Time (VET)
- Total Peripheral Resistance (TPR)
- Total Peripheral Resistance Index (TPRI)
- Cardiac Power (CP)
- Cardiac Power Index (CPI)
- Blood Oxygenation (SPO2)
- Oxygen Delivery Index (DO2I)
- Electrical impendance of the chest cavity (Z0)
- Thoracic Fluid Content (TFC)
- Thoracic Fluid Content change from preset time period (TFCd) and
- Thoracic Fluid Content from baseline (TFCd0)
- Changes in SV, CO (*SVxHR*) and other hemodynamic parameters which are derived by Bioreactance® as a result of posture

The Starling SV monitor is intended for use within hospitals and other healthcare facilities that provide patient care.

More information is available in the "Baxter's Noninvasive Starling SV monitor with NIBP and SpO2" documentation. The 'Declaration of Conformity Starling April 2021', 'Starling

Brochure', 'Starling ED Brochure', Starling System Specifications', 'Baxter Starling Monitor User Guide' are added in the attachments.

Notification: The medical device: Baxter's Noninvasive Starling SV monitor with NIBP and SpO2 is approved by the Medical Device Committee (MMC – UMCG) on august 26, 2023. (Case number - 142815)

6.2 Summary of findings from non-clinical studies

Not applicable. No medicinal product.

6.3 Summary of findings from clinical studies

The clinical studies conducted on the Starling SV monitor have yielded a substantial amount of evidence supporting its effectiveness and safety in various clinical settings. The device offers a dynamic assessment of fluid responsiveness and has been validated through multiple clinical settings. For instance, the phase shift changes observed in the Starling SV monitor have been correlated with thermodilution cardiac output in a sample of 65,000 patients. (17,18) Additionally, independent validation studies have demonstrated its reliability compared to pulmonary artery catheter. (17)

One notable advantage of the Starling SV monitor is its accuracy, which remains unaffected by vasopressors or shock states (Waldron et al., 2014). Furthermore, it has proven to be effective not only in mechanically ventilated patients but also in spontaneously breathing patients at the emergency department (ED). (8,18,19)

In a retrospective study involving nearly 200 patients, the implementation of SV-guided resuscitation was associated with improved patient outcomes. This included a reduced length of stay in the intensive care unit (ICU), lower risk of mechanical ventilation, and decreased initiation of renal replacement therapy (RRT). (20) The study also estimated that implementing SV-guided resuscitation could lead to significant cost savings, amounting to $14.5K per treated patient (20).

The FRESH study focused on sepsis patients admitted to the ICU and aimed to guide hemodynamic resuscitation using dynamic assessments of fluid responsiveness with the Starling SV monitor. The primary clinical outcome assessed was positive fluid balance at 72 hours or ICU discharge. The results indicated that physiologically informed fluid and vasopressor resuscitation, guided by the Starling SV monitor, was safe and associated with lower net fluid balance. This approach also resulted in reductions in the risks of renal and respiratory failure, suggesting that dynamic assessments for fluid administration could improve outcomes for septic shock patients compared to standard care. (14)

Copy

6.4 Summary of known and potential risks and benefits

This intervention guided by the Starling SV monitor offers the benefits of providing sepsis patients with a personalized treatment plan based on their unique hemodynamic changes, aiming to reduce the risk of prolonged hypotension and fluid overload. Utilizing the Starling SV monitor allows for precise fluid administration and timely initiation of vasopressor therapy. This study has the potential to develop personalized medicine and improve treatment strategies for sepsis patients in the emergency department and provides an opportunity to map the hemodynamic endotype of each individual. While the risks associated with the use of the Starling SV monitor are minimal, potential adverse events, device malfunction, misinterpretation of readings, and reliance solely on the monitor without considering other factors may pose risks. Proper training, adherence to protocols, and regular evaluation can help minimize these risks and ensure patient safety.

6.5 Description and justification of route of administration and dosage

Not applicable. No medicinal product.

6.6 Dosages, dosage modifications and method of administration

Not applicable. No medicinal product.

6.7 Preparation and labelling of Non-Investigational Medicinal Product

Not applicable, no medicinal product.

6.8 Drug accountability

Not applicable, no medicinal product.

Copy

7. NON-INVESTIGATIONAL PRODUCT
Not applicable.

Copy

8. METHODS

8.1 Study parameters/endpoints

8.1.1 Main study parameter/endpoint

The primary end point is the volume (in liters) of IV fluid resuscitation within three hours since ED admission (excluding fluids that are administered pre-hospital)

8.1.2 Secondary study parameters/endpoints

Efficacy

Hospital admission	• Length-of-stay in hospital<br>• ICU admission within 7 days (excluding subjects receiving protocolized vasopressor therapy)
New onset organ failure	Any of the following within 48 hours:<br>• Rise in SOFA score of at least 2<br>• Organ support (i.e. vasopressor use [non-]invasive mechanical ventilation, acute dialysis, ECMO)
Fluid resuscitation	• Volume of IV fluid resuscitation within three hours (including prehospital fluids administered)<br>• Volume of IV fluid resuscitation within 7 days<br>• Fluid balance within three hours (i.e difference between volume of intravenous plus oral fluids and<br>the volume of fluid output (i.e. urine output and other fluid losses))
Hemodynamic resuscitation	• Time to recovery of normal perfusion/hemodynamic stability (HDS)*<br>• Proportion of patients reaching normal perfusion/hemodynamic stability* within three hours

* Hemodynamic stability (HSD) is defined as MAP ≥ 70 mmHg. SBP ≥ 90 mmHg, lactate ≤ 4 mmol/L and SI ≤ 0.9.

Safety

Congestive heart failure	• Pre-tibial edema rise of ≥ 2 (grade 1-4), measured at triage, after 3h, 24h and 48h<br>• PoCUS congestion assessment, after 3h, and 24h (e.g. IVC >2 cm and collapse <50%; wet lung ≥ 3 B lines in any given region)<br>• Loop diuretic use within 7 days, determined daily<br>• Decompensated heart failure within 7 days, based on the physicians' judgement
Respiratory insufficiency and acute respiratory distress syndrome (ARDS)	Any of the following within 7 days:<br>• PaO2/FiO2* < 53 kPA<br>• (Non-)invasive mechanical ventilation or ECMO

Copy

Acute kidney injury	• AKI based on KDIGO criteria within 48 hours<br>• AKI based on KDIGO criteria within 7 days<br>• Change in Serum Creatinine Levels from Baseline to 48 hours<br>• Change in Serum Creatinine Levels from Baseline to day 7 (or hospital discharge, whichever came first)<br>• Requirement for renal replacement therapy (RRT) within 7 days<br>• Requirement for renal replacement therapy (RRT) within 30 days
Major Adverse Cardiac Event (MACE)	Any of the following within 30 days:<br>• Cardiovascular death<br>• Nonfatal myocardial infarction<br>• Nonfatal stroke
Vasopressor therapy	• Vasopressor therapy within three hours<br>• Vasopressor therapy within 7 days<br>• Time to vasopressor therapy
All-cause mortality	• Mortality within 48 hours<br>• In-hospital mortality<br>• Mortality within 30 days

Health technology

Health economy	• Cost-effectiveness (*i.e.,* based on length of stay in hospital, advanced care requirement, costs of measurements)

Copy

Exploratory mechanistic endpoints

Hemodynamic endotype	• Starling SV monitor parameters (e.g. CI, TPRI, SVI) up to three hours after ED arrival<br>• Hemodynamic parameters (e.g. systolic blood pressure, diastolic blood pressure, MAP, heart rate, lactate, CRT) up to three hours after ED arrival<br>• Photoplethysmography waveform characteristics (e.g. systolic and diastolic peak amplitude) during ED stay<br>• Electrocardiography waveform characteristics (e.g. pulse-transit-time and heart rate variability (HRV)-features) during ED stay

8.1.3 Other study parameters (if applicable)

Not applicable.

8.2 Randomisation, blinding and treatment allocation

Subjects will be randomized to either the treatment or control arm of the study, ideally at the moment the treating physician decides to admit the patient to the ED. This randomization process begins as soon as the patient is registered for the ED, even before their arrival.

The randomization process at ED arrival will be facilitated by the REDCap randomization module within REDCap electronic data capture tools hosted at the UMCG. Initially, a researcher will identify potential study participants who meet the inclusion criteria based on information provided by the treating physician. Each prospective participant will be assigned a pseudo-anonymized screening ID generated within the REDCap application. This screening ID will encompass both the deferred consent process and the randomization procedure. The randomization will occur through the REDcap module during the screening, determining the assigned treatment arm.

Once the patient arrives and is included in the study, the assigned treatment arm will be communicated to both the research and treatment teams. Any patients that are enrolled but are found to be ineligible based on exclusion criteria will be exited from the study at that point.

Subjects will be randomized in a 1:1 allocation ratio (treatment to control). It is important to note that the randomization process is not blinded in this study. Both the investigator and the physician are aware of the treatment strategy assigned to each subject.

Copy

8.3 Study procedures

Screening and enrollment
Patients will be recruited by the FLUIDS research team. First, ED physician and/or nurse will screen all patients entering the ED for potential inclusion in the study, specifically between 9 am and 5 pm during all weekdays. If a patient is eligible for participating in the study, the ED physician/nurse will promptly notify a FLUIDS-researcher present at the ED. Subsequently, this researcher will approach and enrol the eligible patient, being actively involved in the initial assessment and providing recommendations to the attending physician regarding hemodynamic resuscitation.

The FLUIDS research team will consist of fully trained individuals with a medical background and research affinity. Prior to the study start, the research team will undergo comprehensive training on utilizing the Starling SV monitor, interpretating results and conducting PoCUS measurements. Additionally, a follow-up procedure will be established, where the research team is responsible for implementing the Starling SV monitor protocol during subsequent assessments.

Starling SV monitor procedure
Once the subject has been enrolled, either the investigator or researcher will proceed to place the Starling SV monitor on the subject. The Starling SV monitor will be connected to the subject by positioning four sensors from the system on the subject's chest: specifically, on the left and right upper and lower thorax. The device provides real-time data that can be observed on the monitor screen and is also stored internally. Fluid assessment can be instantly performed by examining the visible read-out on the monitor screen.

Prior to commencing the study, the device will be assessed to ensure optimal and expected hemodynamic readings can be obtained, following the guidelines outlined in the Starling SV

Instructions for Use (IFU). This step is crucial to ensure that the data collected from the monitoring devices will be valid and usable. It is recommended to allow approximately one minute for the device to auto-calibrate and establish a stable baseline before starting any measurements for the study data collection.

In the event that the Starling SV monitor does not provide the expected hemodynamic recordings, adjustments will be made. If necessary, the device will be replaced until the expected recordings are obtained. However, if it is determined that the device cannot provide the expected recordings, the subject will cross-over to the control arm and will be treated according to the standard care protocol.

Fluid responsiveness assessment procedure
A dynamic assessment of fluid responsiveness using the Starling SV monitor will be performed at every clinical decision point for the first three hours of study enrollment. (*see figure 1 'flowchart personalized protocol').* Fluid responsiveness will be assessed in response to an IV fluid bolus of 500 mL in 10 min using the Starling SV hemodynamic monitor. To guide hemodynamic resuscitation, fluid responsiveness will be defined based on an increase in stroke volume in response to the fluid bolus.

Data collection procedure
The information recorded by the Starling SV will be collected and stored within the device, registered under the FLUIDS ID of the subject (coded). Once the data collection period ends, the data will be downloaded and stored for analysis. Other data collected by the researcher will be entered directly in REDCap. A full description and timeline of data collection is presented in **Table 1.**

Copy

Study periods	Screening*	ED visit*	General ward/ICU		Follow up	
		Baseline				
**Hours/days**	**Triage**	**0h**	**24h**	**48h**	**7d**	**30d**
Deferred consent						x
Review of in-/exclusion criteria	x					
Diagnosis of infection/sepsis (adjudication by an expert panel)	x					x
The presence of compression stocks	x					
Demographic data (i.e. age, sex, ethnicity, length, weight, BMI)		x				
Medication use (i.e. cardiovascular, immunosuppressiva, diuretics)		x	x	x		
Co-morbidity (pre-existent, developing)	x		x	x	x	x
Mortality (in-hospital, out-of-hospital)			x	x	x	x
Hospital admission (i.e. general ward, ICU)		x	x	x	x	x
Disease severity scores (i.e. SIRS, [q]SOFA, NEWS)	x	x	x	x	x**	
Vital parameters (i.e. SBP, DBP, heart rate, respiratory rate, SpO2, temperature, GCS, CRT)	x	x	x	x		
Lab values (i.e. leukocytes (including machine differential), thrombocytes, creatinin, bilirubin, CRP, albumin, ureum)	x		x	x	x	
Extended lab values (i.e. pH, lactate, NT-proBNP)	x		x			
Organ support information (i.e. hemodynamic support, RRT, [non-]invasive mechanical ventilation, ECMO)	x	x	x	x	x	x
Volume of IV fluids (time, amount, type)	x	x	x	x		
Vasopressor therapy (time, amount, type)		x	x	x		
(Elektro)physiological waveform data: (e.g.. PPG, ECG)		x				
PoCUS data (e.g. IVC diameter, collapsibility index, lung US)		x	x			
Pre-tibial edema score		x	x	x		
Cost-effectiveness data (i.e. costs and wins)		x				x

Copy

Table 1: description and timeline of data collection * Screening and ED visit occur on the same day. ** Follow up of severity scores is up to 7 days (or hospital ward discharge, whichever came first); hospital ward discharge comprises admission to the ICU or discharge home.

8.4 Withdrawal of individual subjects

Subjects can leave the study at any time for any reason if they wish to do so without any consequences. The investigator can decide to withdraw a subject from the study for urgent medical reasons.

8.4.1 Specific criteria for withdrawal (if applicable)

Not applicable.

8.5 Replacement of individual subjects after withdrawal

Subjects withdrawn from the study will be replaced by a subsequent patient that presents in the ED matching the inclusion criteria.

8.6 Follow-up of subjects withdrawn from treatment

After withdrawal of an individual subject from the study, all data, blood samples collected up to that point will be stored and kept in the database. Upon withdrawal, the subject from the intervention group will receive treatment according to the standard care UMCG-protocol, while the control group will continue with their existing treatment regimen. It is important to note that the measurements for the study will no longer be conducted for the withdrawn subject.
In the event that the participant does not provide consent or withdraws consent, it is up to the participant to indicate whether the data collected up to that point may be retained or must be destroyed.

8.7 Premature termination of the study

Not applicable.

Copy

SAFETY REPORTING

8.8 Temporary halt for reasons of subject safety

In accordance to section 10, subsection 4, of the WMO, the sponsor will suspend the study if there is sufficient ground that continuation of the study will jeopardise subject health or safety. The sponsor will notify the accredited METC without undue delay of a temporary halt including the reason for such an action. The study will be suspended pending a further positive decision by the accredited METC. The investigator will take care that all subjects are kept informed.

8.9 AEs, SAEs and SUSARs

8.9.1 Adverse events (AEs)

Adverse events are defined as any undesirable experience occurring to a subject during the study, whether or not considered related to the intervention procedure. All adverse events reported spontaneously by the subject or observed by the investigator or his staff will be recorded.

8.9.2 Serious adverse events (SAEs)

A serious adverse event is any untoward medical occurrence or effect that
- results in death;
- is life threatening (at the time of the event);
- requires hospitalisation or prolongation of existing inpatients' hospitalisation;
- results in persistent or significant disability or incapacity;
- is a congenital anomaly or birth defect; or
- any other important medical event that did not result in any of the outcomes listed above due to medical or surgical intervention but could have been based upon appropriate judgement by the investigator.

Copy

An elective hospital admission will not be considered as a serious adverse event.

All SAEs will be evaluated and recorded if relationship is unknown or if SAE is related to study procedures. The sponsor will report the SAEs through the web portal *ToetsingOnline* to the accredited METC that approved the protocol, within 7 days of first knowledge for SAEs that result in death or are life threatening followed by a period of maximum of 8 days to complete the initial preliminary report. All other SAEs will be reported within a period of maximum 15 days after the sponsor has first knowledge of the serious adverse events.

8.9.3 Suspected unexpected serious adverse reactions (SUSARs)

Not applicable.

8.10 Annual safety report

Not applicable.

8.11 Follow-up of adverse events

All AEs will be followed until they have abated, or until a stable situation has been reached. Depending on the event, follow up may require additional tests or medical procedures as indicated, and/or referral to the general physician or a medical specialist.
SAEs need to be reported till end of study within the Netherlands, as defined in the protocol.

8.12 Data Safety Monitoring Board (DSMB) / Safety Committee

Not applicable.

Copy

9. STATISTICAL ANALYSIS

All collected data will be stored in the REDcap, and a complete dataset will be uploaded into R version 1.4.1106 using RStudio (*2015, RStudio Team, Boston, MA, United States*) for further descriptive and statistical analysis. The main analysis will be conducted based on the intention-to-treat (ITT) principle. Descriptive statistics, such as mean, median, standard deviation, and confidence intervals, will be reported for continuous variables. Categorical variables will be summarized using frequencies and percentages.

The ITT analysis will include all subjects who have signed the deferred consent (DC), met the study eligibility criteria, and were randomized to either the treatment or control arm of the study. The ITT population will be the primary and secondary analysis population for this study.

Depending on the changes in treatment arms and protocol violations, we will conduct both a per protocol (PP) sensitivity analysis and a modified intention-to-treat (mITT) analysis. These analyses will be performed to assess the robustness of the study's findings and account for variations in treatment adherence and protocol deviations.

The per protocol analysis will include only the subjects who strictly adherent to the study protocol, completed the assigned treatment as planned, and did not have any major protocol violations. The mITT analysis will include subjects who met the eligibility criteria, were randomized, and received at least one part of the allocated treatment, such as one fluid bolus based on a fluid responsiveness measurement. Unlike in the ITT analysis, participants who were randomized but did not receive any treatment will be excluded from the mITT analysis.

9.1 Primary study parameter(s)

The primary endpoint is an assessment of the difference between the two treatment groups in the volume (in liters) of IV fluid resuscitation at three hours (excluding prehospital fluids).. Statistical analysis will be conducted to compare baseline characteristics and outcomes between the study arms. Non-normally distributed data will be analyzed using the Mann-Whitney U test, while normally distributed data will be analyzed using an independent samples Student's t-test. A p-value of <0.05 will be considered significant.

Copy

9.2 Secondary study parameter(s)

Secondary endpoints are included in statistical analysis regardless of the outcome of the primary endpoint . Similar to the primary analysis, differences between the study groups will be analyzed using the Mann-Whitney U test for non-normally distributed data and an independent samples Student's t-test for normally distributed data. A p-value of <0.05 will be considered significant.
For binary and time-to-event analyses concerning secondary study parameters, a detailed analysis plan will be developed and finalized in a timely manner.

9.3 Supplementary subgroup analysis

A supplementary subgroup analysis of the intervention has been scheduled to further investigate the difference between the fluid responders and non-fluid responders. Specifically, the difference in intravenous (IV) fluid resuscitation volume (in liters) at three hours will be examined between the fluid responders and non-fluid responders in the intervention group.

9.4 Missing data

Subjects participating in this study will be monitored for a relatively brief duration, and it is anticipated that missing data will occur at a minimal rate. Measures will be implemented to mitigate the occurrence of missing data. These measures encompass comprehensive training

and consistent monitoring protocols, among other strategies, aimed at minimizing the likelihood of missing data.

9.5 Other study parameters

Not applicable.

9.6 Interim analysis

Not applicable.

Copy

10. ETHICAL CONSIDERATIONS

10.1 Regulation statement

The study will be conducted according to the principles of the Declaration of Helsinki (version, date, see for the most recent version: www.wma.net) and in accordance with the Medical Research Involving Human Subjects Act (WMO) and other guidelines, regulations and Acts.

10.2 Recruitment and consent

Eligible patients are recruited upon arrival at the Emergency Department (ED). To ensure timely intervention in this acute setting, a deferred consent (DC) strategy is employed, facilitated by a proxy if necessary. For DC rationale, see "Rationale for Deferred Consent (DC) Procedure" below.

11.2.1. Recruitment and consent method

1. Upon ED admission, one of the researchers identifies potential study participants meeting the inclusion criteria by information provided by the treating physician. If possible, the patient and/or his/her designated proxy is informed about the study by the researcher, and their consent is sought. If a patient and/or his/her designated proxy does not orally refuse to participate, the patient will be recruited for the study.
2. Once recruited, the patient is randomized according to the study protocol and informed about the randomization results. Following randomization, treatment is promptly initiated as per protocol.
3. The patient themselves and/or his/her designated proxy will be asked to provide deferred consent (DC) within 30 days of the study start. Information about the study will be provided as soon as possible after study entry, which may be at the ED, ICU, or general ward in hospital. If the subject regains mental competence and is capable, their deferred consent for the continuation of their participation in the study must be obtained without undue delay. In cases where the patient is discharged from the hospital and no consent have been obtained, they may be contacted up to three times, and a letter is mailed to obtain deferred consent. Data collection is carried out for a maximum of 30 days from the time of ED arrival. All contact moments will be registered in REDcap' screening field of 'deferred consent'.
4. If the subject does not provide deferred consent, they must be actively informed of the deletion of data previously collected in the study. Throughout the study, participants who signed deferred consent retain the right to withdraw at any time. However, by signing deferred consent, they grant permission to use their collected data for research purposes up to the point of withdrawal.
5. In accordance with the CCMO guidelines on 'deferred consent' in emergency research situations, if a patient passes away before deferred consent is obtained within the 30-day limit, consent for data use is assumed and not sought from legal representatives due to the following reasons:
    a. Relatives do not have an independent right to review or assess therapeutic or research data as they are no longer legal representatives.
    b. The patient's wishes cannot be formally established post-mortem, and relatives no longer have a formal role as representatives in the research context.
    c. The research under 'deferred consent' has been approved by an ethics committee, prioritizing patient benefits over risks.
    d. Using data from deceased participants can enhance the reliability of research outcomes, benefiting society and future patients.
    e. Ethical considerations and transparency require informing relatives about data usage, but their objections may not necessarily lead to data exclusion, depending on the circumstances.

Copy

   f. If, however, the deceased patient objects against research through a registration in the UMCG Objection Registry, than further data collected will be halted immediately and data that is collected thus far will be destroyed.
6. The CCMO guideline emphasizes that, from a transparency and ethical standpoint, it is reasonable to inform the patients' family about their study participation in the event of their passing. This communication is important for both providing closure for the family and the completion of the study. Families will be notified through a letter that includes a brief summary of the study and their family member's participation. Contact information is included in the letter.

11.2.2 Rationale for deferred consent (DC) procedure

Deferred consent (DC) is crucial in an acute setting where immediate treatment initiation is imperative within one hour of arrival at the Emergency Department (ED). In such critical situations, patients often require urgent medical interventions, and any delay in treatment could have serious consequences for their health and well-being. In emergency medical research, obtaining traditional informed consent upfront may not be feasible due to the time-sensitive nature of the conditions and the need for rapid intervention. By applying a deferred consent (DC) strategy, researchers can prioritize the prompt delivery of essential treatments while ensuring that participants' rights and welfare are still protected.
Our study's approach strikes a balance between the urgent medical needs of the patients and the ethical principles of respecting participants' autonomy and welfare in research practices. Moreover, our study constitutes low-risk emergency research with a broad therapeutic window and harboring the potential for patients to benefit from their study inclusion. (21) Article 6, paragraph 4 of the Medical Research Involving Human Subjects Act (WMO) states that, under strict conditions, subjects may participate in medical research in emergency situations without their (or their legal representative's) prior written consent. Accordingly, our study's consent procedure meets the stringent criteria outlined in the deferred consent procedural guide of the Centrale Commissie Mensgebonden Onderzoek (CCMO). (22) With these considerations in place, we can ethically and effectively implement a deferred consent strategy.

Copy

10.3 Objection by minors or incapacitated subjects

Minors will not be enrolled in the trial. Incapacitated patients will be enrolled in the trial, including those with severe sepsis or septic shock, as these conditions can cause the patient to have decreased consciousness. Legal representatives will be informed of the patient's participation in the study and will be asked to provide deferred consent either during treatment or afterward. Additionally, patients who recover and regain capacity can provide consent up to 30 days after receiving treatment at the emergency department.

10.4 Benefits and risks assessment, group relatedness

Benefits
This intervention study provides patients with a distinctive and personalized treatment plan tailored to their individual hemodynamic changes resulting from infection. By utilizing the Starling SV monitor to assess fluid responsiveness, patients receive a precise administration of IV fluids or prompt initiation of vasopressor therapy based on their specific needs. This approach aims to reduce the risk of prolonged hypotension and fluid overload. Furthermore, this opportunity has the potential to develop personalized medicine and improve treatment strategies for sepsis patients in the emergency department. Additionally, considering the diverse and varied presentation of sepsis as a disease, we have the opportunity to map the hemodynamic endotype of each individual sepsis patient by collecting comprehensive hemodynamic parameters from the database of this study.

Risks
The risks associated with using the Starling SV monitor are minimal. There is a slight chance of experiencing minor adverse events, such as skin irritation from the adhesive on the chest wall, though this is generally not expected to be severe. Additionally, there is the potential for the device to malfunction or provide inaccurate readings, as with any medical device. Improper use or misinterpretation of the device's readings by healthcare professionals could lead to incorrect treatment decisions or delays. Moreover, relying solely on the monitor's readings without considering other clinical factors and patient assessments may result in suboptimal patient care or adverse events. To mitigate these risks, it is crucial for healthcare providers to receive proper training in using the device, follow established protocols, and regularly evaluate its performance. This ensures the safety and effectiveness of the device in clinical practice.

Furthermore, incorrect guidance from the Starling SV monitor may lead to insufficient fluid administration, causing hypotension, or excessive fluid administration, resulting in fluid overload. To address these concerns, all patients will receive a standard 1.5 L per day of intravenous fluids after the ED visit, with variations based on factors such as cardiovascular comorbidities. Intravenous fluid administration will be limited to a maximum of 60 ml/kg over three hours to prevent hypervolemia. Additionally, fluid resuscitation effects, i.e. fluid status and tolerance, will be assessed using Point-of-Care Ultrasound (PoCUS) at different time points during the three-hour and 48-hour follow-up period.
By combining the use of the Starling SV monitor with these precautions, we aim to achieve a more precise estimate of the fluids needed and reduce the risks associated with both hypovolemia and hypervolemia.

Discussion
In this intervention study, the utilization of the Starling SV monitor provides significant benefits for sepsis patients in the emergency department. By tailoring treatment plans to individual hemodynamic changes, patients receive precise fluid administration and timely initiation of vasopressor therapy, reducing the risks of prolonged hypotension and fluid overload. This personalized approach has the potential to optimize sepsis management and improve outcomes. Although there are minimal risks associated with the monitor, such as minimal adverse events and the potential for device malfunction or misinterpretation of readings, proper training, adherence to protocols, threshold determination and regular evaluation of treatment effect can mitigate these risks and ensure patient safety. Overall, the benefits of the Starling SV monitor outweigh its minimal risks, making it a valuable tool in improving patient outcomes.

10.5 Compensation for injury

The sponsor/investigator has a liability insurance which is in accordance with article 7 of the WMO.

The sponsor (also) has an insurance which is in accordance with the legal requirements in the Netherlands (Article 7 WMO). This insurance provides cover for damage to research subjects through injury or death caused by the study.
The insurance applies to the damage that becomes apparent during the study or within 4 years after the end of the study.

10.6 Incentives

Not applicable.

Copy

11. ADMINISTRATIVE ASPECTS, MONITORING AND PUBLICATION

11.1 Handling and storage of data and documents

Data is handled confidentially by treating physicians and research nurses. A subject identification code list is used to link the data to the subject. The key to the code is safeguarded by the investigator(s). The code is a random number and will not contain any data of the patient, like date of birth or initials, which would enable direct identification of the patient from the code. The handling of personal data will comply with the EU General Data Protection Regulation and the Dutch Act on Implementation of the General Data Protection Regulation. (in Dutch: Uitvoeringswet AVG, UAVG). The computerized files are stored in the FLUIDS study' research drive in the UMCG network. The files are encrypted and password protected. De-identified data may be copied to other computers within the UMCG-network for analysis but will remain password protected. Research data will be stored for 15 years starting from the end of the study.

11.2 Monitoring and Quality Assurance

Since the current protocol is a low-risk study, monitoring will take place once per year. Therefore, a monitor plan will be drawn up before the start of the study and a monitor (internal UMCG pool) will be invited to monitor the study.

11.3 Amendments

Copy

Amendments are changes made to the research after a favourable opinion by the accredited METC has been given. All amendments will be notified to the METC that gave a favourable opinion.

A 'substantial amendment' is defined as an amendment to the terms of the METC application, or to the protocol or any other supporting documentation, that is likely to affect to a significant degree:

- the safety or physical or mental integrity of the subjects of the trial;
- the scientific value of the trial;
- the conduct or management of the trial; or
- the quality or safety of any intervention used in the trial.

All substantial amendments will be notified to the METC and to the competent authority.

Non-substantial amendments will not be notified to the accredited METC and the competent authority but will be recorded and filed by the sponsor.

11.4 Annual progress report

The sponsor/investigator will submit a summary of the progress of the trial to the accredited METC once a year. Information will be provided on the date of inclusion of the first subject, numbers of subjects included and numbers of subjects that have completed the trial, serious adverse events/ serious adverse reactions, other problems, and amendments.

11.5 Temporary halt and (prematurely) end of study report

The investigator/sponsor will notify the accredited METC of the end of the study within a period of 8 weeks. The end of the study is defined as the last patient's last visit.

The sponsor will notify the METC immediately of a temporary halt of the study, including the reason of such an action.

In case the study is ended prematurely, the sponsor will notify the accredited METC within 15 days, including the reasons for the premature termination.

Within one year after the end of the study, the investigator/sponsor will submit a final study report with the results of the study, including any publications/abstracts of the study, to the accredited METC.

11.6 Public disclosure and publication policy

Results of this study will be disclosed unreservedly.

Copy

12. STRUCTURED RISK ANALYSIS

12.1 Potential issues of concern

a. Level of knowledge about mechanism of action

The Starling SV monitor is a noninvasive hemodynamic monitoring device that operates based on the principles of impedance. It works by measuring changes in electrical impedance within the chest cavity, which correspond to changes in the amount of blood present in the thorax during the cardiac cycle.

The device consists of four adhesive electrodes that are placed on the patient's chest. Two of these electrodes act as current injectors, sending a low-level, high-frequency electrical signal through the chest. The other two electrodes serve as voltage detectors, measuring the resulting changes in impedance as the electrical signal encounters variations in the thoracic fluid volume during the cardiac cycle.

As the heart pumps blood, there are fluctuations in thoracic fluid volume that correspond to different phases of the cardiac cycle, such as ventricular ejection and filling. By continuously measuring these impedance changes, the Starling SV monitor can derive various hemodynamic parameters, including stroke volume, cardiac output, and cardiac indices.

The derived hemodynamic parameters help healthcare providers assess a patient's fluid status, fluid responsiveness, cardiac performance, and vascular resistance. This information can be crucial in guiding fluid resuscitation, optimizing cardiac function, and making timely clinical decisions for patients with sepsis or other conditions that affect hemodynamics.

Copy

b. Previous exposure of human beings with the test product(s) and/or products with a similar biological mechanism

In the FRESH study, participants underwent hemodynamic resuscitation guided by the Starling SV monitor. The study focused on sepsis patients admitted to the Intensive Care Unit (ICU) with the objective of directing hemodynamic resuscitation through dynamic assessments of fluid responsiveness using the Starling SV monitor. The primary clinical endpoint evaluated was the presence of positive fluid balance at 72 hours or upon discharge from the ICU.

The findings indicated that employing physiologically informed fluid and vasopressor resuscitation, guided by the Starling SV monitor, was both safe and associated with reduced net fluid balance. This approach also led to a decrease in the risks of renal and respiratory failure. These results suggest that implementing dynamic assessments for fluid administration could yield improved outcomes for septic shock patients compared to standard care practices.(14)

c. Can the primary or secondary mechanism be induced in animals and/or in *ex-vivo* human cell material?

Not applicable, no medicinal product.

d. Selectivity of the mechanism to target tissue in animals and/or human beings

Not applicable, no medicinal product.

e. Analysis of potential effect

Not applicable, no medicinal product.

f. Pharmacokinetic considerations

Not applicable, no medicinal product.

g. Study population

The study participants are not healthy volunteers but patients who present to the ED with suspected or confirmed infection and signs of hemodynamic instability. These patients are enrolled during their admission to the emergency department when their condition, although not stable, is not immediately life-threatening. Immediate hemodynamic resuscitation is implemented in both the intervention and control groups, and this does not pose any additional risk for sepsis treatment. The key distinction between the groups lies in the personalized approach to hemodynamic resuscitation.

h. Interaction with other products

Not applicable, no medicinal product.

i. Predictability of effect

Not applicable, no medicinal product.

j. Can effects be managed?

Not applicable, no medicinal product.

Copy

12.2 Synthesis

The risks associated with the utilization of the Starling SV monitor are minimal. Firstly, there is a slight possibility of experiencing minimal adverse events related to the device. For instance, there might be a chance of skin irritation caused by the adhesive on the chest wall, although this is not expected to be severe. Secondly, as with any medical device, there is a potential for device malfunction or inaccurate readings. Thirdly, if healthcare professionals improperly use or misinterpret the device's readings, it could result in incorrect treatment decisions or delays. Moreover, relying solely on the readings from the Starling SV monitor without considering other clinical factors and patient assessments may lead to suboptimal patient care or adverse events. However, it is essential for healthcare providers to receive proper training in using the device, adhere to established protocols, and regularly evaluate its performance to minimize risks and ensure the safety of patients.

13. REFERENCES

1. Napolitano LM. Sepsis 2018: Definitions and Guideline Changes. Surg Infect (Larchmt). 2018;19(2):117–25.
2. Taeb AM, Hooper MH, Marik PE. Sepsis: Current definition, pathophysiology, diagnosis, and management. Nutr Clin Pract. 2017;32(3):296–308.
3. Rudd KE, Johnson SC, Agesa KM, Shackelford KA, Tsoi D, Kievlan DR, et al. Global, regional, and national sepsis incidence and mortality, 1990–2017: analysis for the Global Burden of Disease Study. Lancet. 2020;395(10219):200–11.
4. Gotts JE, Matthay MA. Sepsis: Pathophysiology and clinical management. BMJ [Internet]. 2016;353. Available from: http://dx.doi.org/doi:10.1136/bmj.i1585
5. Young JD. The heart and circulation in severe sepsis. Br J Anaesth. 2004;93(1):114–20.
6. Gordon D, Spiegel R. Fluid Resuscitation: History, Physiology, and Modern Fluid Resuscitation Strategies. Emerg Med Clin North Am. 2020 Nov;38(4):783–93.
7. Brown RM, Semler MW. Fluid Management in Sepsis. J Intensive Care Med. 2019;34(5):364–73.
8. Bentzer P, Griesdale DE, Boyd J, MacLean K, Sirounis D, Ayas NT. Will this hemodynamically unstable patient respond to a bolus of intravenous fluids? JAMA - J Am Med Assoc. 2016;316(12):1298–309.
9. Kelm DJ, Perrin JT, Cartin-Ceba R, Gajic O, Schenck L, Kennedy CC. Fluid overload in patients with severe sepsis and septic shock treated with early goal-directed therapy is associated with increased acute need for fluid-related medical interventions and hospital death. Shock. 2015;43(1):68–73.
10. Rivers, Emanuel; Nguyen, Bryant; Havstad, Suzanne; Ressler, Julie; Muzzin, Alexandria; Knoblich, Bernhard; Peterson, Edward; Tomlanovich M. Early Goal-Directed Therapy in the Treatment of Severe Sepsis and Septic Shock. N Engl J Med. 2001;345(19):1368–77.
11. Cecconi M, Fasano N, Langiano N, Divella M, Costa MG, Rhodes A, et al. Goal-directed haemodynamic therapy during elective total hip arthroplasty under regional anaesthesia. Crit Care. 2011;15(3):R132.
12. Mehta Y, Kapoor PM, Maheswarappa HM, Saxena G. Noninvasive Bioreactance-Based Fluid Management Monitoring: A Review of Literature. J Card Crit Care TSS. 2021;05(03):222–8.
13. Oord M, Olgers TJ, Doff-Holman M, Harms MPM, Ligtenberg JJM, Ter Maaten JC. Ultrasound and NICOM in the assessment of fluid responsiveness in patients with mild sepsis in the emergency department: A pilot study. BMJ Open. 2017;7(1):1–6.
14. Douglas IS, Alapat PM, Corl KA, Exline MC, Forni LG, Holder AL, et al. Fluid Response Evaluation in Sepsis Hypotension and Shock: A Randomized Clinical Trial. Chest. 2020;158(4):1431–45.
15. Mcgregor D, Sharma S, Gupta S, Ahmad S, Godec T, Harris T. cardiac output study (EDNICO): a feasibility and repeatability study. Scand J Trauma Resusc Emerg Med. 2019;1–9.
16. McGregor D, Sharma S, Gupta S, Ahmed S, Harris T. Emergency department non-invasive cardiac output study (EDNICO): An accuracy study. Scand J Trauma Resusc Emerg Med. 2020;28(1):1–9.
17. Squara P, Denjean D, Estagnasie P, Brusset A, Dib JC, Dubois C. Noninvasive cardiac output monitoring (NICOM): A clinical validation. Intensive Care Med. 2007;33(7):1191–4.
18. Raval NY, Squara P, Cleman M, Yalamanchili K, Winklmaier M, Burkhoff D. Multicenter evaluation of noninvasive cardiac output measurement by bioreactance technique. J Clin Monit Comput. 2008;22(2):113–9.
19. Duus N, Shogilev DJ, Skibsted S, Zijlstra HW, Fish E, Oren-Grinberg A, et al. The reliability and validity of passive leg raise and fluid bolus to assess fluid responsiveness in spontaneously breathing emergency department patients. J Crit Care. 2015;30(1):217.e1-217.e5.
20. Latham HE, Bengtson CD, Satterwhite L, Stites M, Subramaniam DP, Chen GJ, et al. Stroke volume guided resuscitation in severe sepsis and septic shock improves outcomes. J Crit Care. 2017;42(2017):42–6.
21. Fitzpatrick, A., Wood, F. & Shepherd, V. Trials using deferred consent in the emergency setting: a systematic review and narrative synthesis of stakeholders' attitudes. Trials 23, 411 (2022).
22. Ministerie van Volksgezondheid, Welzijn en Sport. (2020, February 25). Stappenplannen voor uitgestelde toestemming ('deferred consent') bij onderzoek in noodsituaties. Nieuwsbericht | Centrale Commissie Mensgebonden Onderzoek. https://www.ccmo.nl/actueel/nieuws/2020/02/25/stappenplannen-voor-uitgestelde-toestemming-deferred-consent-bij-onderzoek-in-noodsituaties

Copy
