# Supplementary material for: FLUid management and InDividualized resuscitation in Sepsis (FLUIDS)—A Study protocol for a single-centre, open-label, randomized clinical trial
Source: PLoS One. 2025 Dec 19;20(12):e0338504. doi: 10.1371/journal.pone.0338504 (PMC12716701; doi:10.1371/journal.pone.0338504)
Supplement: S5 File — FLUid management and InDividualized resuscitation in Sepsis (FLUIDS). This illustration shows the study design of the FLUid management and InDividualized resuscitation in Sepsis (FLUIDS) trial, an open-label randomized controlled trial investigating personalized hemodynamic resuscitation in early septic shock at the emergency department. A total of 188 adult patients with suspected sepsis who require hemodynamic resuscitation are randomly assigned during the initial three-hour resuscitation phase to receive either fluid and vasopressor therapy guided by changes in stroke volume index (ΔSVI) or standard care based on the treating physician’s discretion. The primary outcome is the volume of intravenous fluids administered within the first three hours after study enrollment. Secondary outcomes include the cumulative fluid balance, and the timing and dose of vasopressors, the incidence of organ failure, hospital length of stay, and venous congestion as assessed by point-of-care ultrasound. By continuously and non-invasively measuring fluid responsiveness, the study aims to offer a personalized approach to optimize fluid administration according to individual hemodynamic needs, which can improve organ perfusion and reduce the risk of fluid overload at the same time. Deferred consent will be obtained within 30 days after ED presentation. Created in BioRender. (PDF) [file pone.0338504.s005.pdf]

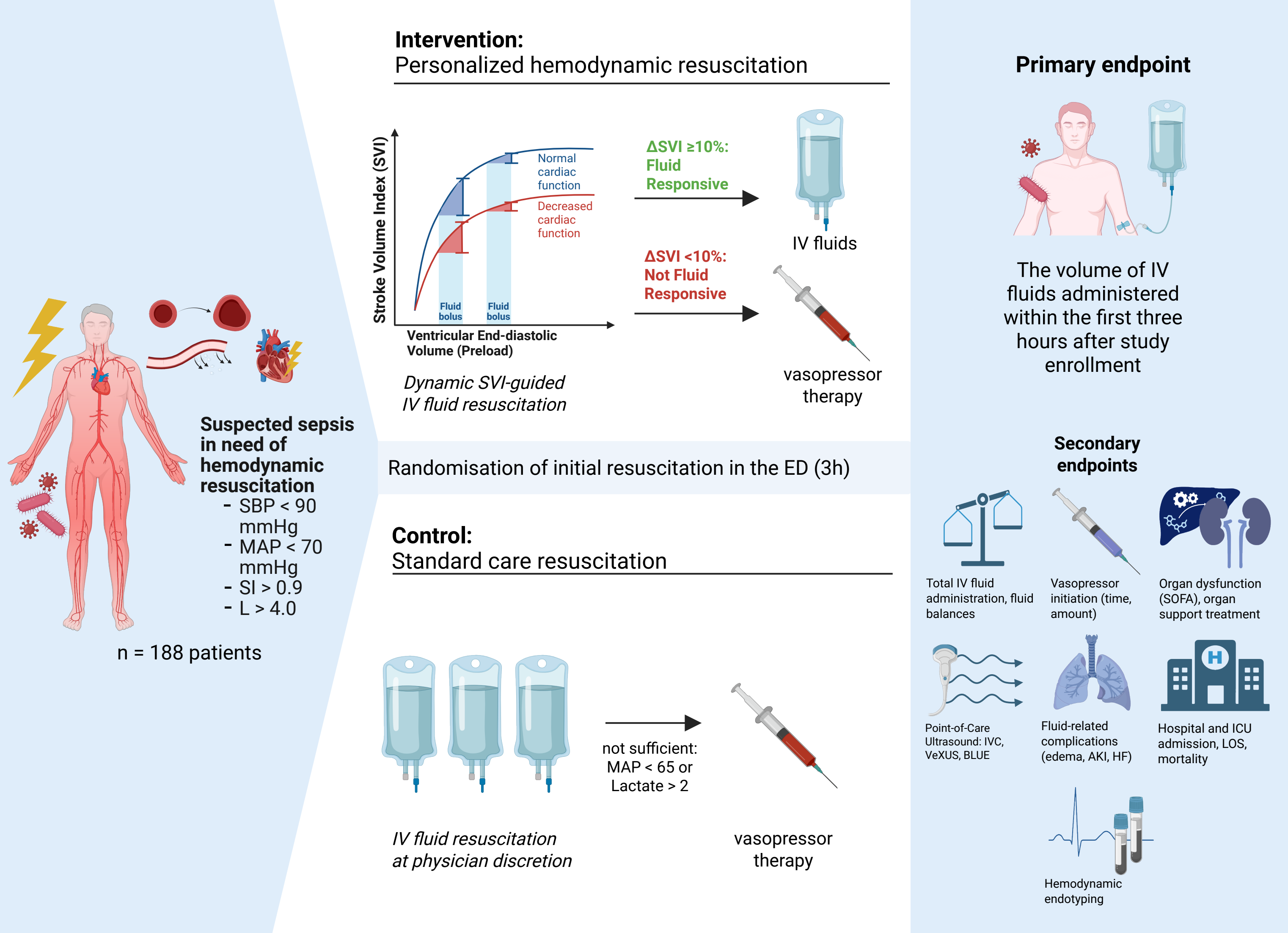
Intervention:
Personalized hemodynamic resuscitation
Stroke Volume Index (SVI)
Normal cardiac function
Decreased cardiac function
Fluid bolus
Fluid bolus
Ventricular End-diastolic Volume (Preload)
Dynamic SVI-guided IV fluid resuscitation
ΔSVI ≥10%: Fluid Responsive
IV fluids
ΔSVI <10%: Not Fluid Responsive
vasopressor therapy
Suspected sepsis in need of hemodynamic resuscitation
- SBP < 90 mmHg
- MAP < 70 mmHg
- SI > 0.9
- L > 4.0
n = 188 patients
Randomisation of initial resuscitation in the ED (3h)
Control:
Standard care resuscitation
not sufficient: MAP < 65 or Lactate > 2
IV fluid resuscitation at physician discretion
vasopressor therapy
Primary endpoint
The volume of IV fluids administered within the first three hours after study enrollment
Secondary endpoints
Total IV fluid administration, fluid balances
Vasopressor initiation (time, amount)
Organ dysfunction (SOFA), organ support treatment
Point-of-Care Ultrasound: IVC, VeXUS, BLUE
Fluid-related complications (edema, AKI, HF)
Hospital and ICU admission, LOS, mortality
Hemodynamic endotyping
