# Supplementary material for: FLUid management and InDividualized resuscitation in Sepsis (FLUIDS)—A Study protocol for a single-centre, open-label, randomized clinical trial
Source: PLoS One. 2025 Dec 19;20(12):e0338504. doi: 10.1371/journal.pone.0338504 (PMC12716701; doi:10.1371/journal.pone.0338504)
Supplement: Attachment — Submitted filename: Response to reviewers.docx [file pone.0338504.s006.docx]

**Reviewer #1:**

**Thank you for the opportunity to review your manuscript which is a protocol for a randomized trial on fluid management and individualized resuscitation in sepsis. This trial will begin enrolling in the next couple weeks.**

1. **Introduction**
    **- Well written; you could refer to the ESICM guideline aon fluids which suggested an individualized approach to fluid resuscitation**

We have added the recent ESICM guideline on fluids, which recommends an individualized approach to fluid resuscitation, as suggested.

2. **Methods**
    **- TPRI used in Exclusion criteria but spelt out laterTPRI (minor revision)**

We have adjusted the text appropriately.

**- SAEs should be more prescriptive as opposed to what people spontaneously report**

We have revised the manuscript to clarify that serious adverse events (SAEs) are defined according to institutional criteria and are only reported if potentially related to the study intervention or if the relationship is uncertain. Events clearly attributable to the underlying disease (e.g., death from septic shock) are not considered SAEs. This provides a more prescriptive definition in line with regulatory standards while distinguishing SAEs from spontaneously reported adverse events.

Revised text:

*“Adverse events are any undesirable experiences occurring during the study. All AEs reported spontaneously by the subject or observed by the investigator or his staff, will be recorded. Serious adverse events (SAEs) are defined as events potentially related to the study intervention and meet institutional criteria for seriousness (e.g., death, life-threatening event, hospitalization, persistent disability, or any other medically event). Events clearly attributable to the underlying disease, such as death from septic shock, are not considered SAEs. Reported SAEs will be documented in accordance with institutional and regulatory requirements to ensure participant safety throughout the study.”*

**Overall, as you can see from the above, I only have minor revisions. This is a well written and thought out protocol.**

3. **Some things to consider for a larger version of this study (if you wish; just my opinion for your consideration as I understand that this study is starting soon):**
    **- It may be advantageous to have a primary outcome that's a composite (i.e. mortality, AKI, need for RRT, fluid balance [dichotomized] etc)**
    **- A TEE arm or including it as part of your protocol if good windows aren't available with TTE**
    **- Consider measuring L sided pressures with TDI in protocol**
    **- Should cirrhotic patients be excluded or be part of a subgroup? What about CKD/HD patients?**

Thank you for your suggestions. For a future (multi)center studies, it would indeed be interesting to include clinical effect endpoints. In the current proof-of-principle study, however, our primary focus is on the direct effects of personalized hemodynamic guidance on fluid administration, aiming to demonstrate that this approach can reduce total fluid volume and allow earlier initiation of vasopressors. Key secondary endpoints do include organ dysfunction, mortality, need for renal replacement therapy, and fluid balances.

Regarding TEE, in the emergency department setting we aim to use non-invasive tools, as TEE is relatively invasive for spontaneously breathing or non-sedated patients. TEE could be considered in ICU research, but it is not feasible for our current ED study. In future ED-ICU studies, its use could be considered. While measuring left-sided pressures with Tissue Doppler Imaging (TDI) can provide valuable hemodynamic information, it is challenging to implement in the emergency department setting due to the need for high-quality echocardiographic windows and trained operators, particularly in spontaneously breathing or unstable patients. TDI could be considered in future ED-ICU studies or as part of an optional sub-study.

As per your suggestion, we have added CKD/HD patients and patients with decompensated liver cirrhosis at arrival to the exclusion criteria. This update will be incorporated in a forthcoming amendment of our protocol.

Revised text:
*“Patients will be excluded when they received >1 L IV fluid prior to randomization, when IV access cannot be gained, when non-invasive SVI and total peripheral resistance index (TPRI) measurements cannot be obtained reliably (e.g., due to intra-abdominal hypertension, ascites), or if the patient cannot be enrolled within 1 hour after ED presentation. Moreover, patients with a primary diagnosis of acute cerebral vascular event, acute coronary syndrome, acute pulmonary edema, status asthmaticus, major cardiac arrhythmia, drug overdose, burn or trauma-related injury, diabetic ketoacidosis, hyperosmolarity syndrome, or pancreatitis are also excluded. Other exclusion criteria include known aortic insufficiency, aortic abnormalities, or intraventricular heart defects (e.g., ventricular or atrial septal defects); advanced heart failure (NYHA class IV, on a waiting list for heart transplant, LVAD recipients, or chronic inotrope use), end-stage kidney disease (dialysis-dependent CKD stage 5 or eGFR <15 mL/min/1.73 m²), and decompensated liver cirrhosis at ED admission (e.g., ascites, hepatic encephalopathy, or variceal bleeding). Additional exclusion include pregnancy, other causes of shock (e.g., hemorrhagic shock), anticipated surgery within 3 hours of presentation, or contraindications to vasopressor therapy (e.g., advanced care directive). Finally, patients transferred from another hospital after initiation of therapy or from another in-hospital setting will be excluded.”*

**Reviewer #2:**

**This is a protocol with ethics approval for a single site RCT comparing a non-invasive measure of cardiac output and fluid responsiveness to guide fluid management with routine clinician judgment in patients with a working diagniosis of sepsis**
 **Recruitment will start soon (or has started).**

**Overall this is fairly well written, although I have a few comments which may help frame the paper/RCT a bit better.**

**There are a few key issues with the design and methodology.**

**1. Primary outcome is difference in fluid given in the first 3 hrs. This is really a feasibility and proof of concept outcome. If your intervention dictates a different volume given, then this will have an effect on your outcome. In other words – your intervention correlates with the outcome.**

The primary aim of our study is to demonstrate a proof-of-principle, assessing the direct impact of a personalized hemodynamic treatment approach. We acknowledge that the primary outcome (fluid volume administered in the first 3 hours) is influenced by the intervention itself. This is intentional: our intervention guides therapy based on real-time changes in stroke volume index rather than clinician judgment alone, allowing us to evaluate whether a personalized approach can more precisely tailor fluid therapy and avoid under- or over-resuscitation.

In this context, the correlation between intervention and outcome is not a limitation but a feature: reflecting the measurable effect of the intervention on clinical decision-making. Patient-centered endpoints such as organ failure, mortality, and hypervolemia are included as secondary outcomes, providing additional insight and informing sample size calculations for a larger multicenter follow-up trial.

Revised text:

*“Using IV fluid administered as the primary endpoint means the study is potentially not powered to detect differences in clinical outcomes, such as sepsis-associated organ dysfunction and mortality. However, this endpoint directly reflects the immediate effect of the intervention and is appropriate for a proof-of-principle study. If the results are promising, this single-center study will provide a foundation for future (international) multicenter studies.”*

**2. Furthermore, it would be good to speculate what a relevant difference is. The sample size was based on a study that showed a volume difference at 72 hours, not 3 hours. This means that even if your initial indivualised approach shows a effect, there may well be a catch up effect at 24 and 72 hours. Please explore and explain this. I suspect that you are heavily underpowered. In an ideal word I would think that the minimum possibly clinically important difference in volume in the first 3 hours would be 750-1000ml (based on Andrews et al** [**https://pubmed.ncbi.nlm.nih.gov/28973227/**](https://pubmed.ncbi.nlm.nih.gov/28973227/) **)**

Both Andrews et al. and Douglas et al. reported a mean difference in fluid administration of approximately 1.3–1.5 L, which helps define a clinically relevant effect. We chose Douglas et al. as the primary reference for our design because their SVI-guided methodology closely matches our intervention, even though their study assessed a later phase of resuscitation in the ICU after patients had already received ~2 L of fluids. Our study focuses on the early resuscitation phase in the emergency department, where the majority of fluids are typically administered within the first 3 hours. We recognize that a catch-up effect in fluid volumes may occur at later time points. Focusing on this early window allows us to evaluate whether individualized, stroke-volume–guided therapy meaningfully influences initial fluid administration.

Based on our updated calculations, accounting for unequal variance, 85 patients per arm (170 total), plus 10% dropout (188 total), is sufficient to assess the early impact of the intervention. By demonstrating feasibility and proof-of-principle, this study will provide important data to inform future studies that assess downstream clinical outcomes.

Revised text:
*“Sample size was calculated using R (version 4.3.1), based on data from a comparable study with similar methodology and endpoint definitions (21). That reference study demonstrated a mean difference of 1.37 L in positive fluid balance at 72 hours between groups, with the intervention group showing 0.65 ± 2.85 L compared to 2.02 ± 3.44 L in the control group (21). The effect size (Cohen's d = 0.45) was calculated from the reported means and standard deviations.*

*We conducted an a priori power analysis via a Monte Carlo simulation (50,000 replicates) assuming normal distributions for each arm with observed means and standard deviations. To accommodate unequal variances, a two-tailed Welch’s t-test (α = 0.05) was applied in the simulated dataset. The simulation showed that 170 participants (85 per group) are needed to achieve 80% power to detect a statistically significant difference in the primary endpoint (IV fluid volume administered within the first three hours after study enrollment). Anticipating 10% drop-out due to loss to follow-up, study withdrawal, or protocol deviations, the total planned sample size is 188 participants (94 per group).”*

 **3. Cliniciians will be interested in duration of vaspossors, need for renal replacement, duration of ventilation and mortalit**

Vasopressor therapy, need for renal replacement therapy, invasive ventilation (both incidence and duration), and mortality are included as key secondary endpoints assessing efficacy and safety of the personalized resuscitation protocol compared to standard care.

**4. The complexity and accuracy of the intervention needs to be better spelled out. The paper states it is accurate compared to Swan Ganz, but I am not aware of any ICUs still using Swan Ganz cathers. All other papers do not seem to be in patients with sepsis.**

We have updated the information on the accuracy of the Starling device and included relevant references.

Revised text:

“*The accuracy of this non-invasive technique has been evaluated in septic and critically ill patients. While absolute agreement with thermodilution is limited (percentage errors often 48–67%) (Galarza 2018, Shih 2016), bioreactance reliably detects relative changes in stroke volume. In elderly septic shock patients, a PLR-induced change in cardiac output measured by bioreactance predicted fluid responsiveness with 86% sensitivity and 79% specificity (Hu et al. 2017). In mixed ICU cohorts, bioreactance showed concordance rates of ~75–80% for tracking stroke volume changes compared with transpulmonary thermodilution (Zhu 2020, Galarza 2018), and accuracy improves when shorter averaging/refresh times are applied (Galarza 2018, Gavelli 2021). Thus, although absolute values are not interchangeable with thermodilution, the monitor provides clinically useful information on directional changes in stroke volume in septic patients.*”

**5. Further, the accuracy (sens/spec vs which gold standard) of the POCUS measures (such as VEXUS and BLUE) need better justification. In my reading these protocols are still to be interpreted cautiously, especially with interrater reliability and absence of true gold standard. Fluid congestion is again a proxy outcome - possibly helpful to inform a future study.**

We agree with the reviewer that PoCUS protocols such as VeXUS and BLUE should be interpreted with caution given the absence of a golden standard and interrater variability. In our study, these measures are not used as a golden standard for validation of the SVI-guided resuscitation. Rather, they are applied to monitor signs of venous congestion and thereby assess the safety of the intervention. Importantly, congestion may occur even in fluid-responsive patients (Muñoz et al. 2024), underlining that fluid responsiveness and fluid tolerance are distinct. Our aim is to identify early signs of fluid overload during initial resuscitation, before organ dysfunction develops, and to clarify the relationship between responsiveness and tolerance to inform future personalized resuscitation strategies.

Added section to manuscript:

***2.6 Point-of-Care Ultrasound***

*“Point-of-care ultrasound (POCUS) is a rapid, noninvasive bedside tool increasingly used to assess hemodynamic status and guide fluid management in sepsis. Protocols including inferior vena cava (IVC) assessment, bedside lung ultrasound in emergency (BLUE), and the venous excess ultrasound (VeXUS) score provide complementary information on intravascular volume, pulmonary congestion, and venous congestion (Argaiz, 2021; Arvig 2022; Ahn 2025; Klompmaker 2025). POCUS offers real-time qualitative and quantitative data, potentially detecting early physiologic changes not apparent on standard clinical examination. Importantly, fluid congestion can occur even in fluid-responsive patients (Munoz, 2024), highlighting that fluid responsiveness and fluid tolerance are overlapping but distinct concepts.*

*To ensure consistent measurements, we implemented structured training, standardized acquisition protocols, image archiving, and blinded expert review (Blanco 2016, Assavapokee 2024), as detailed in section 2.7 and 2.11. Using this approach, we aim to monitor early signs of venous congestion and assess the safety of SVI-guided resuscitation, explore the hemodynamic interplay between fluid responsiveness and tolerance, and generate quantitative physiologic insights a to inform personalized resuscitation strategies and future studies.”*

**The corollary of the above is that this really should be framed as a feasibility or pilot RCT to show that fluid volume separation can be achieved with this approach (defined as a volume not effect size), with the secondary clinical outcomes being measured which can inform a future multicentre RCT.**

We have revised the discussion to emphasize the proof-of-principle nature of our study. We acknowledge the limitation of using fluid volume administered as the primary endpoint, as the study is likely not powered to detect differences in all sepsis-associated organ dysfunctions or mortality. Nevertheless, this proof-of-principle study is designed to generate valid evidence on early, individualized fluid management, demonstrating that separation in fluid volumes can be achieved. The data obtained will inform the design of future multicenter RCTs, ultimately aiming to improve personalized resuscitation and fluid management in sepsis.

Revised text:

*“The FLUIDS study aims to evaluate the benefit of a personalized hemodynamic resuscitation strategy, providing tailored interventions based on real-time fluid responsiveness measurements that reflect the dynamic state of the individual patient with sepsis in the ED. This approach, supported by advanced monitoring technologies, enables more accurate decision-making during the critical early resuscitation phase. By focusing on this early phase, when patients have received few or no therapies, the study captures the optimal window to assess hemodynamic status and guide resuscitation, unlike ICU studies, where patients have often already received substantial fluids. It also includes a broader ED population, from less severely ill patients to those developing refractory shock, capturing a diverse patient population in which optimal fluid strategies are often uncertain, particularly in those with comorbidities such as heart failure. Comprehensive data collection, including vital signs, capillary refill time, organ dysfunction, fluid balance, PoCUS assessments, physiological waveform data, and biomarker measurements, provides a multidimensional understanding of patient hemodynamics. The FLUIDS research team, part of Acutelines data-biobank, is well-trained, with expertise in both the Starling SV monitor and PoCUS, ensuring high-quality data collection and adherence to the study protocol. Moreover, the use of Acutelines ICT infrastructure supports the efficient and automated collection of data from the bed-side monitor. At last, the structured, personalized protocol in the intervention arm including systematic monitoring of fluid responsiveness and vital signs, along with predefined rescue rules, ensures consistent and reproducible treatment decisions, with the goal of providing optimal, individualized care for patients with sepsis in the ED.*

*The study has several limitations. First, the lack of blinding may introduce bias, as both the research and treatment teams are aware of treatment allocation, potentially influencing patient management and outcome assessments. Blinding the study protocol is not feasible, as both the researcher and treating physician must follow the protocol to ensure proper adherence. Moreover, awareness of fluid-related risks and benefits through trial information may lead physicians in the control (standard care) arm to adopt a more restrictive IV fluid strategy. Individual physician decision-making could further introduce variability in the control arm, which may affect the amount of IV fluids administered and should be considered when interpreting results. Second, conducting the study at a single center may limit the generalizability of the findings to other emergency departments with different patient populations or resources. Using IV fluid administered as the primary endpoint means the study is potentially not powered to detect differences in clinical outcomes, such as sepsis-associated organ dysfunction and mortality. However, this endpoint directly reflects the immediate effect of the intervention and is appropriate for a proof-of-principle study. If the results are promising, this single-center study will provide a foundation for future (international) multicenter studies.*

*In conclusion, the FLUIDS study integrates a comprehensive design, a well-trained, experienced team, and structured personalized protocols using real-time fluid responsiveness monitoring in the emergency department. It aims to generate robust evidence on early, individualized fluid management, informing the design of future trials and ultimately improving FLUid management and InDividualized resuscitation in Sepsis.”*

**Further minor comments:**

**5. Abstract – The statement that clinicians use a fixed amount is not justified. It is quite clear from the literature that clinicians have large practice variation in hemodynamic support (**[**https://pubmed.ncbi.nlm.nih.gov/32043315/**](https://eur03.safelinks.protection.outlook.com/?url=https%3A%2F%2Fpubmed.ncbi.nlm.nih.gov%2F32043315%2F&data=05%7C02%7Cs.ter.horst%40umcg.nl%7Cf1493327f035451fbba708dde94998eb%7C335122f9d4f44d67a2fccd6dc20dde70%7C0%7C0%7C638923224528741344%7CUnknown%7CTWFpbGZsb3d8eyJFbXB0eU1hcGkiOnRydWUsIlYiOiIwLjAuMDAwMCIsIlAiOiJXaW4zMiIsIkFOIjoiTWFpbCIsIldUIjoyfQ%3D%3D%7C0%7C%7C%7C&sdata=MGXDJJOUgaq0IekWWLL4z3ZrgeGh7FsdKAjdxagK24A%3D&reserved=0) **. This is probably based on clinician experience, setting, type of patient (respiratory sepsis may be treated more judiciously) and an already indvidualised approach (patient CCF vs renal failure will be approached differently anyway) e. These variable that may influence fluids administration should be considered as predefined subgroups (ED vs ICU, junior vs senior, source of sepsis, existing comorbidities)**

We agree that there is substantial variability in initial fluid administration in the emergency department, influenced by clinician experience, patient comorbidities, and the source of sepsis.

Revised text:

*“****Introduction*** *Early septic shock results from complex hemodynamic disturbances, including vasodilation, capillary leakage, and hypovolemia, necessitating timely and targeted resuscitation to prevent organ failure. In the emergency department (ED), initial fluid administration varies widely, influenced by clinician experience, patient comorbidities, and the source of infection. Despite this variability, fluid therapy is often not tailored to each patient's individual fluid responsiveness and tolerance, highlighting the need for more personalized resuscitation strategies.*

***Objective*** *This study evaluates the effectiveness of a personalized hemodynamic resuscitation strategy during the initial management of sepsis in the ED, using continuous non-invasive stroke volume index (SVI) measurements to guide intravenous fluid therapy and support early vasopressor initiation.”*

**6. Methods – are surgical causes of sepsis excluded?**

Yes, for the current study we focus on non-surgical (non-trauma) causes of sepsis, as the acute care infrastructure in our ED (Acutelines) is built around these specialties. For future research, we plan to expand the study to include surgical causes of sepsis once the initial methodology and workflow are established.

**7. SIRS criteria are mentioned but I wonder if it would be better to leave it at clinical judgment. SIRS was replaced for a reason (poor specificty)**

To clarify, inclusion in the study is based on the physician’s clinical judgment upon arrival at the ED. We mention criteria such as body temperature, leukocyte count, C-reactive protein, and symptoms suggestive of infection merely as examples to guide clinical assessment, not as strict SIRS-based criteria. This approach aligns with current practice, recognizing that SIRS criteria have limited specificity and are no longer the standard for defining sepsis.

**8. I am also surprised to see the shock index as a measure. My understanding is that it performs poorly. At what time does the SI have to be >0.9. At any time?**

In our study, the shock index (SI) is measured at the first available vital signs upon ED arrival. We chose a threshold of SI >0.9 because it allows us to capture patients in the “grey zone” of early septic shock. Using heart rate alone is too nonspecific, while isolated hypotension (<90 mmHg) already identifies more advanced shock. The SI combines heart rate and systolic blood pressure, thereby identifying patients with relative hypotension (e.g., SBP around 100 mmHg with tachycardia), who are at increased risk of hemodynamic deterioration but may otherwise be missed. This population is of particular interest, as they often require early resuscitation, and decisions about the amount and timing of fluids and vasopressors are most challenging.

Recent evidence supports this threshold. Several systematic reviews (Middleton 2019, Diaztagle Fernandez 2024) have examined the shock index, showing that values >0.9 (range 0.7–1.0) are associated with a high risk of deterioration, including ICU admission and mortality. For example, Al Aseri et al. (2020) evaluated the shock index to determine a cutoff that identifies patients at risk of hemodynamic collapse requiring vasopressors. They found that an SI >0.87 predicted fluid non-responsiveness and the subsequent need for vasopressors, supporting its role as an early risk stratification tool for hemodynamic collapse and septic shock in the emergency department.

**9. 2.3 Randomisation I am surprised that patients are randomised at triage. I assume you mean after meeting inclusion criteria? This could be at 20 min after triage, but patients may also develop sepsis 3 hrs into their ED stay, so only become eligible to be randomised then. Please clarify**

Patients are randomized at triage to specifically capture the initial resuscitation phase. While this approach may result in some patients who develop sepsis later during their ED stay being missed, the majority of patients in our ED are referred via the primary care pathway and present with fully developed sepsis at the time of ED admission. This strategy ensures that the intervention targets the earliest possible phase of resuscitation, which is the focus of our study.

**10. More detail on accuracy (sens/spec vs which gold standard ) of the SV monitor needs to be in the body of the text. The references are now includings studies in dogs (Beagles) and patients with pulmonary hypertension, but no reference discusses sepsis. I think the reader needs to be convinced that this monitor is actually doing what it is supposed to and not a random number generator**

We have revised the manuscript to provide more detail on validation studies in septic and critically ill patients. Absolute agreement with thermodilution is limited, with percentage errors of 48–67%, but the monitor reliably detects dynamic changes in stroke volume. For example, in elderly septic shock patients a PLR-induced change in cardiac output predicted fluid responsiveness with 86% sensitivity and 79% specificity (Hu et al. 2017). Other ICU studies report concordance rates of ~75–80% for tracking changes compared with transpulmonary thermodilution (Zhu 2020, Galarza 2018), particularly when shorter averaging times are applied (Galarza 2018, Gavelli 2021). We believe this additional detail clarifies that the device is not a substitute for thermodilution in absolute terms, but does provide reliable information on directional changes, which is the clinically relevant endpoint in septic resuscitation.

Revised text:

“*The accuracy of this non-invasive technique has been evaluated in septic and critically ill patients. While absolute agreement with thermodilution is limited (percentage errors often 48–67%) (Galarza 2018, Shih 2016), bioreactance reliably detects relative changes in stroke volume. In elderly septic shock patients, a PLR-induced change in cardiac output measured by bioreactance predicted fluid responsiveness with 86% sensitivity and 79% specificity (Hu et al. 2017). In mixed ICU cohorts, bioreactance showed concordance rates of ~75–80% for tracking stroke volume changes compared with transpulmonary thermodilution (Zhu 2020, Galarza 2018), and accuracy improves when shorter averaging/refresh times are applied (Galarza 2018, Gavelli 2021). Thus, although absolute values are not interchangeable with thermodilution, the monitor provides clinically useful information on directional changes in stroke volume in septic patients*”

**Reviewer #3:**

**The manuscript is generally well written; however, it can be further improved based on the comments below.**

**1. Line 24-32 Abstract: In the objectives section, the write-up from the introduction should be separated from the actual objective.**

We have revised the abstract to clearly separate the background/introduction from the specific study objective.

**2. Line 33-47: A separate subtitle, ‘Method,’ should be included, with all relevant sections organized under this heading.**

To address this comment, we revised the abstract format. Instead of separate subsections (design, setting, participants, interventions, and study endpoints), we combined these into a single comprehensive Methods section. The abstract now consists of the following sections: Introduction, Objective, Methods, and Expected Results.

Revised text:

***“Methods*** *This single-centre, open-label, randomized clinical trial at the Emergency Department of the University Medical Centre Groningen, the Netherlands will enroll 188 adults with suspected sepsis requiring hemodynamic resuscitation, defined by a mean arterial pressure (MAP) <70 mmHg, systolic blood pressure (SBP) <90 mmHg, shock index >0.9, or lactate concentration >4.0 mmol/L. Patients will be randomized to the intervention group, in which fluids and vasopressors will be guided by changes in SVI (ΔSVI) after IV fluid administration, or to the control group, where fluids and vasopressors will be administered at the discretion of the treating physician during the initial 3-hour resuscitation phase. The primary endpoint is the volume of IV fluids administered within the first three hours after enrollment. Secondary endpoints include cumulative fluid balance, timing and dose of vasopressor initiation, incidence of organ failure, hospital length of stay, and adverse events, including venous congestion assessed by point-of-care ultrasound.”*

**3. Line 242: Table 1: The symbol ** is missing in the table.**

Upon review, the symbol ** is not missing in Table 1; it is only applied to the relevant variables as indicated. Disease severity scores and laboratory values do not require this symbol, and the table reflects this correctly.`

**4. Line 298: Focusing only on hospital stay duration, advanced care needs, and intervention costs in health economic assessments is insufficient. It would be more comprehensive to also include parameters such as quality of life, health outcomes, and indirect costs.**

Thank you for this suggestion. We have expanded the health-economic section to include clinical outcomes (readmissions and in-hospital/30-day mortality), which are feasible within the current protocol. We did not add quality-of-life or indirect-cost measures because these require additional follow-up, resources and participant burden beyond what was approved; we acknowledge their value and plan to address them in future research.

Revised text:
*“Health economic assessments will focus on cost-effectiveness based on length of hospital stay, advanced care needs, intervention costs, and clinical health outcomes including readmissions and in-hospital/30-day mortality.”*

**5. Line 340: The multiple imputation (MI) method is applicable for most cases of missing data, especially if the missing data is missing completely at random (MCAR) or missing at random (MAR) and missingness is moderate (e.g., less than 30%). If the missing data is missing not at random (MNAR), MI may not fully correct the bias.**

We have revised the manuscript to explicitly state the assumptions underlying multiple imputation (MCAR/MAR) and its limitations if data are missing not at random (MNAR). To address this, we will perform sensitivity analyses in case MNAR patterns are suspected, to ensure the robustness of our findings.

Revised text:
“*As the amount of data to be collected for this study is substantial, we have implemented specific measures to mitigate the risk of missing data. Most importantly, we make use of dedicated research staff for this project, who can focus entirely on obtaining the measurement and follow-up data. Further, we use the ICT infrastructure developed by Acutelines that facilitates automated collection of data from the bed-side monitors (26). Missing data will be reported transparently and managed in accordance with CONSORT guidelines for randomized controlled trials. For analyses, we will primarily apply data imputation techniques, such as multiple imputation, to minimize bias and maximize statistical power (43,44). We acknowledge that multiple imputation assumes data are missing completely at random (MCAR) or missing at random (MAR), and recognize that if data are missing not at random (MNAR), this approach may not fully eliminate bias; in such cases, we will perform sensitivity analyses to assess the robustness of results. In instances where crucial variables, such as primary endpoints, are missing and cannot be reliably imputed, affected participants will be excluded from the corresponding analyses, with reasons and extent of exclusions clearly documented.*”`

**6. Line 358: In the G*Power sample size calculation, more details need to be specified, such as the test family, statistical test, type of power analysis, and whether a one- or two-tailed test was applied.**

In light of the reviewer’s concern, we revisited our power analysis. Because variance between groups is unequal, we adjusted our a priori statistical test accordingly. We now specify the exact power analysis in the Methods. The calculation was performed in R (version 4.3.1). We conducted an a priori power analysis via a Monte Carlo simulation (50,000 replicates) assuming normal distributions for each arm with observed means and standard deviations. To accommodate unequal variances, a two-tailed Welch’s t-test (α = 0.05) was applied in the simulated dataset. The simulation showed that 170 participants (85 per group) are needed to achieve 80% power to detect a statistically significant difference in the primary endpoint (IV fluid volume administered within the first three hours after study enrollment). Anticipating 10% drop-out due to loss to follow-up, study withdrawal, or protocol deviations, the total planned sample size is 188 participants (94 per group).

Revised text:
*“Sample size was calculated using R (version 4.3.1), based on data from a comparable study with similar methodology and endpoint definitions (21). That reference study demonstrated a mean difference of 1.37 L in positive fluid balance at 72 hours between groups, with the intervention group showing 0.65 ± 2.85 L compared to 2.02 ± 3.44 L in the control group (21). The effect size (Cohen's d = 0.45) was calculated from the reported means and standard deviations.*

*We conducted an a priori power analysis via a Monte Carlo simulation (50,000 replicates) assuming normal distributions for each arm with observed means and standard deviations. To accommodate unequal variances, a two-tailed Welch’s t-test (α = 0.05) was applied in the simulated dataset. The simulation showed that 170 participants (85 per group) are needed to achieve 80% power to detect a statistically significant difference in the primary endpoint (IV fluid volume administered within the first three hours after study enrollment). Anticipating 10% drop-out due to loss to follow-up, study withdrawal, or protocol deviations, the total planned sample size is 188 participants (94 per group).”*

**7. Line 366: In addition to ITT, conducting a per-protocol (PP) analysis as a sensitivity or secondary analysis is recommended to demonstrate the robustness of the results.**

Please, see next comment.

**8. Line 370: Even if protocol violations are rare (<5%), it is still recommended to report both ITT and PP analyses.**

We have revised the statistical analysis plan to explicitly state that, in addition to the primary ITT analysis, a per-protocol (PP) analysis (and, if applicable, a modified ITT analysis) will be conducted as sensitivity analyses to demonstrate robustness of the findings, regardless of the rate of protocol violations.

Revised text:
*“In addition to the ITT analysis, we will conduct a per protocol (PP) sensitivity analysis and/or a modified intention-to-treat (mITT) analysis to assess the robustness of the primary results, regardless of the rate of protocol violations.”*

**9. Line 373: The definition of what constitutes major protocol deviations is to be stated.**
We have clarified the definition of major protocol violations to explicitly include failure to follow the assigned treatment protocol when clinically indicated (e.g., administering a fluid bolus when not indicated, or not initiating vasopressors when indicated).

Revised text:

*“Protocol violations are classified as minor (e.g., early study termination due to ICU transfer or discharge) or major. Major protocol violations are defined as failure to follow the assigned treatment protocol when clinically indicated, such as giving a fluid bolus when not indicated or not starting vasopressors when indicated.”*

**10. Line 374: The sentence requires improvement e.g. Per-protocol (PP) analysis will include only subjects who strictly adhered to the study protocol and had no major protocol violations. Minor deviations will be permitted if the deviations do not impact the primary outcome/endpoint.**

We have improved the sentence in the manuscript to clarify that the per-protocol analysis will include only participants without major protocol violations, while minor deviations that do not affect the primary outcome will be permitted.

Revised text:
*“The per protocol (PP) analysis will include only participants who were strictly adherent to the study protocol without any major protocol violations. Minor protocol violations will be allowed if they do not impact the primary outcome.”*

**11. Line 389: The type of logistic regression should be specified (e.g., Standard Logistic Regression for a single endpoint at a fixed time, Repeated Measures Logistic Regression, or Mixed-Effects Logistic Regression). In addition, the time point(s) of analysis should be clearly stated.**
We have clarified that logistic regression will be standard logistic regression applied at pre-specified time points (e.g., in-hospital or 30-day outcomes). This ensures that the type of regression and timing of the analyses are explicitly defined.

Revised text:

*“For binary outcomes, associations between treatment arms and clinical outcomes (e.g., fluid-related complications, new onset organ dysfunction, mortality) will be assessed using standard logistic regression analysis at pre-specified time points (e.g. in-hospital, 30-day outcomes).”*

**12. Line 409: The statistical software used, including the publisher and version, is to be stated.**

We have added the statistical software used: R (version 4.3.1) in the third paragraph describing the primary steps of the statistical analysis.

Revised text:

*“Statistical analysis will be conducted to compare outcomes between the treatment arms. The primary endpoint (volume of IV fluids administered within the first three hours) will be tested for normality using the Shapiro-Wilk test and analyzed using the appropriate statistical test. Continuous variables with a normal distribution will be analysed using independent samples Student’s t-tests, while non-normally distributed continuous variables will be analysed using the Mann–Whitney U test. Categorical variables will be compared using the Chi-square test. A two-sided p-value of <0.05 will be considered statistically significant. Statistical analyses will be performed using R (version 4.3.1).”*

**13. Line 436-445: The paragraph repeats certain words unnecessarily and could be refined. E.g, ‘In conclusion’, ‘Despite limitations such as lack of blinding and a single-center setting’.**

We have revised the paragraph to reduce repetition and improve flow, while retaining the focus on the study’s proof-of-principle nature and its aim to generate evidence on early, individualized fluid management.

Revised text:

*“The FLUIDS study combines a comprehensive design, a well-trained, experienced team, and structurized personalized protocols using real-time fluid responsiveness monitoring in the emergency department. As a proof-of-principle study, it aims to generate valid evidence on early, individualized fluid management, laying foundation for future trials and ultimately improving FLUid management and InDividualized resuscitation in Sepsis.”*

**Figure 4: The assessment time point is to be highlighted.**

Time points are indicated in bold (emphasized) and point-of-care ultrasound assessment time points are starred, in Figure 4.

**The format of references in the list is to follow the journal format.**

**Reviewer #4:**

**1. Intro**
**Gives good background on the topic and clearly states the aim of the study**

**2. Methods**
**I would advise explaining where the 174 enrollees number came from. I see this is discussed in detail later in the paper, but I would suggest just stating this is from power calculation and refer to the appropriate section. Otherwise this number stands out awkwardly at this point in the paper.**

We have revised the text to clarify the origin of the sample size. Specifically, we now mention that the number of 188 patients is based on a (improved) sample size calculation, which is introduced in Section 2.1 (Study design and setting) and described in detail in Section 3.1 (Sample size calculation).

Revised text:
*“Eligible patients will be enrolled using deferred consent (by proxy) to ensure timely intervention and minimize selection bias in the acute setting. The clinical trial will span 12 months and will include 188 patients, as determined by the sample size calculation (Section 3.1).”*

**3. I would advise defining what is meant by rescue fluids and whether that is a protocoled amount or based on clinical gestalt.**

We have clarified that rescue fluids are protocolized, specifying a 500 mL IV bolus over 10 minutes, administered within the first 3 hours if patients meet predefined criteria: severe or refractory hypotension, rising lactate, sustained tachycardia, or extreme hypovolemia.

Revised text:
*“Rescue fluids can be given at any time and are defined as a protocolized 500 mL IV bolus over 10 minutes, administered if patients meet predefined criteria, including severe or refractory hypotension, elevated lactate, sustained tachycardia, or extreme hypovolemia”*

**4. Questions— is POCUS only going to be used in the SVI-guided resuscitation group or in both the intervention and control groups? Also, what is the purpose of collecting POCUS data—is it purely data gathering or is there an intent to correlate results to NICOM data and associated interventions?**

PoCUS will be performed in both the intervention (SVI-guided resuscitation) and control groups. The primary objective is to assess fluid and congestion status after initial resuscitation. Specifically, we aim to determine whether patients in the intervention group exhibit fewer congestion signals, as measured by the VExUS (Venous Excess Ultrasound) score, compared to those in the control group.

The data collected via PoCUS will be used to assess the safety of the SVI-guided resuscitation strategy by monitoring for signs of venous congestion. In addition, PoCUS will allow us to explore the relationship between fluid responsiveness and venous congestion. Previous studies have demonstrated that these two phenomena can coexist in critically ill patients, highlighting the importance of comprehensive monitoring strategies (Muñoz, 2024). Finally, these data may inform the development of future resuscitation protocols by providing insights into how fluid responsiveness and congestion assessments can be integrated to optimize patient outcomes.

Added section 2.6:

***2.6 Point of Care Ultrasound***

*“Point-of-care ultrasound (POCUS) is a rapid, noninvasive bedside tool increasingly used to assess hemodynamic status and guide fluid management in sepsis. Protocols including inferior vena cava (IVC) assessment, bedside lung ultrasound in emergency (BLUE), and the venous excess ultrasound (VeXUS) score provide complementary information on intravascular volume, pulmonary congestion, and venous congestion (Argaiz, 2021; Arvig 2022; Ahn 2025; Klompmaker 2025). POCUS offers real-time qualitative and quantitative data, potentially detecting early physiologic changes not apparent on standard clinical examination. Importantly, fluid congestion can occur even in fluid-responsive patients (Munoz, 2024), highlighting that fluid responsiveness and fluid tolerance are overlapping but distinct concepts.*

*To ensure consistent measurements, we implemented structured training, standardized acquisition protocols, image archiving, and blinded expert review (Blanco 2016, Assavapokee 2024), as detailed in section 2.7 and 2.11. Using this approach, we aim to monitor early signs of venous congestion and assess the safety of SVI-guided resuscitation, explore the hemodynamic interplay between fluid responsiveness and tolerance, and generate quantitative physiologic insights a to inform personalized resuscitation strategies and future studies.”*

**5. Note: there are two sections labeled 2.9—may want to correct the numbering or sub label one (i.e. 2.9a).**

Corrected as suggested.

**6. This Section otherwise seems complete and well thought out.**

**7. Statistical Methods**
**These seem appropriate.**

**8. Strengths/Limitations**
**The discussion on the importance of the study and acknowledging its limitations are appropriate and well-considered.**

**Reviewer #5:**

**The FLUIDS protocol is a randomized clinical trial that evaluates the effectiveness of individualized fluid resuscitation in patients with sepsis. The trial uses continuous, noninvasive stroke volume index (SVI) monitoring in the emergency department. Given ongoing debates on optimal fluid management in sepsis, the research question is highly relevant. The manuscript adheres to SPIRIT recommendations and presents a clear rationale, making it generally well prepared.**
 **I would like to congratulate the authors on their excellent work.**

**1. Informed consent**
 **The deferred consent procedure is appropriate for the emergency context, but further detail is needed in the manuscript text (not only in the supplement) on how patients or legal representatives will be approached, and how data will be handled in cases of refusal.**

While the model consent form and participant information sheet are provided in the supplementary material (S2 File), the full procedure for contacting patients and obtaining deferred consent (including approach to patients and/or legally authorized representatives, timing of consent, handling of discharged patients, and management of refusal or withdrawal) is fully described in the main manuscript (see Methods, Informed Consent section).

Full section “informed consent”:
*“Eligible patients will be enrolled upon arrival at the Emergency Department (ED). Due to the urgent nature of emergency care and the need for immediate treatment decisions, the Medical Ethics Committee has approved a deferred consent procedure (38,39). The model consent form and participant information sheet (in Dutch) are provided as supplementary material (see S2 File).*

*Patients are initially enrolled in the study without prior consent due to the emergency setting. Whenever possible, the patient and/or their legally authorized representative (LAR) is informed about the study by a member of the research team. If neither the patient (or the LAR) verbally refuses participation, the patient is included in the study. Written informed consent will be sought from patients or their LAR within 30 days of ED admission. Study information will be provided as soon as possible after enrollment, either in the ED, ICU, or general ward. If the patient regains mental competence, their own written consent for continuation of participation must be obtained without undue delay. If the patient is discharged before consent can be obtained, they may be contacted up to three times by phone and/or mail. All contact moments and consent outcomes are documented in the REDCap screening log.*

*If a patient (or their representative) refuses consent when approached, they will be withdrawn from the study and all previously collected data will be deleted, except where retention is required by regulatory authorities for safety reporting. Participants who provide informed consent retain the right to withdraw from the study at any time. Data collected up to the point of withdrawal will be retained for research purposes.”*

**2. End-points**
 **The list of secondary outcomes is extensive and includes efficacy, safety, and exploratory endpoints (organ failure, congestion, biomarkers, mortality). Consider prioritising or categorizing secondary outcomes (key vs exploratory), to avoid issues with multiplicity and to focus the trial’s interpretation.**

In the manuscript, we have emphasized the key secondary endpoints, which include efficacy, safety, and cost-effectiveness measures: “The key secondary outcomes of this study aim to assess the efficacy, safety, and cost-effectiveness of SVI-guided resuscitation compared with standard care.” We have also clearly separated the exploratory mechanistic endpoints, explicitly stating that “Exploratory mechanistic endpoints focus on understanding the underlying physiological processes and are intended to generate hypotheses for future research.” This categorization highlights the prioritization of key outcomes while clearly distinguishing exploratory measures, thereby addressing concerns regarding multiplicity and helping to focus the interpretation of trial results.

Revised text:
“*The key secondary outcomes of this study aim to assess the efficacy, safety, and cost-effectiveness effects of SVI-guided resuscitation compared with standard care. Efficacy endpoints include the cumulative amount of IV fluids and vasopressors administered in three hours, and the timing of vasopressors initiation. Additional efficacy endpoints include new-onset organ failure within 48 hours, defined by an increase in SOFA score of at least two points or the initiation of organ support. Hospital admission, length of hospital stay, ICU admission within seven days, and cumulative fluid resuscitation over seven days, assessed by total IV fluid volume and net fluid balance, are also included. Additionally, secondary measures focus on the time to achieve predefined hemodynamic resuscitation targets (MAP ≥ 70 mmHg, SBP ≥ 90 mmHg, lactate ≤ 4 mmol/L, ≥30% lactate decrease in those with baseline lactate > 2.5 mmol/L, and SI ≤ 0.9) and the proportion of patients meeting these endpoints within three hours.*

*Safety endpoints include the incidence of complications arising from either treatment strategy, such as fluid overload, which will be assessed for signs of congestion by point-of-care ultrasound at 3 hours and 24 hours using protocols such as IVC, VeXUS, and BLUE, and by pre-tibial edema. Additional safety endpoints include the use of loop diuretics within seven days, the occurrence of congestive heart failure, respiratory insufficiency, or acute kidney injury, all monitored up to seven days, and the need for renal replacement therapy within 30 days. Major adverse cardiac events will be assessed up to 30 days, and mortality will be evaluated at 48 hours, during hospitalization, and at 30 days. At last, ealth economic assessments will focus on cost-effectiveness based on length of hospital stay, advanced care needs, intervention costs, and clinical health outcomes including readmissions and in-hospital/30-day mortality.*

*Exploratory mechanistic endpoints focus on understanding the underlying physiological processes and are intended to generate hypotheses for future research. These include (derived) hemodynamic parameters from the starling device (such as cardiac output, total peripheral resistance), lactate, measured within the first three hours after ED arrival, as well as photoplethysmography (PPG) and electrocardiography (ECG) waveform characteristics, which may offer insights into changes in cardiovascular function during the ED stay (40,41). In addition, a set of blood samples will be collected and stored via the Acutelines data biobank infrastructure to enable future exploratory analyses of biomarkers associated with fluid responsiveness, venous congestion, and hemodynamic endotypes (26). All endpoints are described in detail in the study protocol, which can be accessed on ClinicalTrials.gov for further reference*.”

**3. Statistical plan**
**The protocol includes ITT, per-protocol, and modified ITT analyses, which is appropriate. However, further clarification is needed on:**
**1) Adjustments for multiple comparisons given the large number of secondary outcomes.**
**2) The exact covariates included in adjusted regression models (age, sex, comorbidities are mentioned, but disease severity scores could also be considered).**

**Further clarification of adjustments of multiple testing is added, moreover the exact covariates of disease severiy is also added.**

Further clarification regarding adjustments for multiple testing has been added, specifying that the false discovery rate (FDR) will be applied where appropriate for analyses involving multiple secondary outcomes. Additionally, the exact covariates included in adjusted regression models, including measures of disease severity, have been added to the Statistical Analysis section.

Revised text:

*“A two-sided p-value of <0.05 will be considered statistically significant. To account for multiple comparisons among secondary outcomes, the false discovery rate (FDR) method will be applied where appropriate.”*

*“All regression analyses will be adjusted for potential confounders, including age, sex, relevant comorbidities (e.g., heart failure, chronic obstructive pulmonary disease, chronic kidney diseases, and liver disease), and disease severity scores (e.g. NEWS, qSOFA).”*

**Discussion and limitations**
 **The discussion rightly notes the single-centre design and lack of blinding. It should also mention:**
 **1) Possible operator-dependence of PoCUS and Starling measurements.**
 **2) Restricted recruitment hours (9–17 on weekdays) which may introduce selection bias.**
 **3) The challenge of generalisability to other ED settings with different patient populations and resources.**

We have included the limitation regarding the challenge of generalisability to other ED settings with different patient populations and resources in the discussion, and we have updated the “Strengths and limitations” section accordingly.

With regard to points 1 and 2: we do not expect a relevant selection bias due to recruitment hours, as the large majority of eligible patients present between 9:00–17:00, and their characteristics are not dependent on time of presentation. Therefore, we do not anticipate systematic differences between patients included during these hours and those presenting outside this timeframe. Concerning operator-dependence, both PoCUS and Starling measurements were performed by a small, consistently trained study team, which minimizes inter-operator variability. We agree that this may hamper generalizability to settings where a trained team is not available, beyond the study; if applicable, we will mention this as a limitation in the manuscript describing the results. For these reasons, we have not added these aspects as limitations in the manuscript.

Revised text:
*“The study has several limitations. First, the lack of blinding may introduce bias, as both the research and treatment teams are aware of treatment allocation, potentially influencing patient management and outcome assessments. Blinding the study protocol is not feasible, as both the researcher and treating physician must follow the protocol to ensure proper adherence. Moreover, awareness of fluid-related risks and benefits through trial information may lead physicians in the control (standard care) arm to adopt a more restrictive IV fluid strategy. Individual physician decision-making could further introduce variability in the control arm, which may affect the amount of IV fluids administered and should be considered when interpreting results. Second, conducting the study at a single center may limit the generalizability of the findings to other emergency departments with different patient populations or resources. Using IV fluid administered as the primary endpoint means the study is potentially not powered to detect differences in clinical outcomes, such as sepsis-associated organ dysfunction and mortality. However, this endpoint directly reflects the immediate effect of the intervention and is appropriate for a proof-of-principle study. If the results are promising, this single-center study will provide a foundation for future (international) multicenter studies.”*
